# Cationic proteins from eosinophils bind bone morphogenetic protein receptors promoting vascular calcification and atherogenesis

**DOI:** 10.1093/eurheartj/ehad262

**Published:** 2023-06-03

**Authors:** Zhaojie Meng, Shuya Zhang, Wei Li, Yunzhe Wang, Minjie Wang, Xin Liu, Cong-Lin Liu, Sha Liao, Tianxiao Liu, Chongzhe Yang, Jes S Lindholt, Lars M Rasmussen, Lasse M Obel, Jane Stubbe, Axel C Diederichsen, Yong Sun, Yabing Chen, Paul B Yu, Peter Libby, Guo-Ping Shi, Junli Guo

**Affiliations:** Department of Medicine, Brigham and Women’s Hospital and Harvard Medical School, 77 Avenue Louis Pasteur, NRB-7, Boston, MA 02115, USA; Department of Medicine, Brigham and Women’s Hospital and Harvard Medical School, 77 Avenue Louis Pasteur, NRB-7, Boston, MA 02115, USA; Hainan Provincial Key Laboratory for Tropical Cardiovascular Diseases Research & Key Laboratory of Emergency and Trauma of Ministry of Education, Institute of Cardiovascular Research of the First Affiliated Hospital, Hainan Medical University, Haikou 571199, Hainan, China; College of Chinese Medicinal Materials, Jilin Agricultural University, Changchun 130118, Jilin, China; Department of Medicine, Brigham and Women’s Hospital and Harvard Medical School, 77 Avenue Louis Pasteur, NRB-7, Boston, MA 02115, USA; Department of Cardiology, the First Affiliated Hospital of Zhengzhou University, Zhengzhou, Henan, China; Department of Medicine, Brigham and Women’s Hospital and Harvard Medical School, 77 Avenue Louis Pasteur, NRB-7, Boston, MA 02115, USA; Department of Medicine, Brigham and Women’s Hospital and Harvard Medical School, 77 Avenue Louis Pasteur, NRB-7, Boston, MA 02115, USA; Department of Medicine, Brigham and Women’s Hospital and Harvard Medical School, 77 Avenue Louis Pasteur, NRB-7, Boston, MA 02115, USA; Department of Cardiology, the First Affiliated Hospital of Zhengzhou University, Zhengzhou, Henan, China; Department of Medicine, Brigham and Women’s Hospital and Harvard Medical School, 77 Avenue Louis Pasteur, NRB-7, Boston, MA 02115, USA; Department of Medicine, Brigham and Women’s Hospital and Harvard Medical School, 77 Avenue Louis Pasteur, NRB-7, Boston, MA 02115, USA; Department of Medicine, Brigham and Women’s Hospital and Harvard Medical School, 77 Avenue Louis Pasteur, NRB-7, Boston, MA 02115, USA; Department of Geriatrics, National Key Clinical Specialty, Guangzhou First People's Hospital, Guangzhou Medical University, Guangzhou 510000, Guangdong, China; Department of Cardiothoracic and Vascular Surgery, Odense University Hospital, Odense, Denmark; Elite Research Centre of Individualized Treatment for Arterial Disease, University Hospital, Odense, Denmark; Elite Research Centre of Individualized Treatment for Arterial Disease, University Hospital, Odense, Denmark; Department of Clinical Biochemistry, Odense University Hospital, Odense, Denmark; Elite Research Centre of Individualized Treatment for Arterial Disease, University Hospital, Odense, Denmark; Department of Clinical Biochemistry, Odense University Hospital, Odense, Denmark; Cardiovascular and Renal Research unit, Institute for Molecular Medicine, University of Southern Denmark, Odense, Denmark; Elite Research Centre of Individualized Treatment for Arterial Disease, University Hospital, Odense, Denmark; Department of Cardiology, Odense University Hospital, Odense, Denmark; Department of Pathology, The University of Alabama at Birmingham, Birmingham, AL 35294, USA; Birmingham VA Medical Center, Research Department, Birmingham, AL 35294, USA; Department of Pathology, The University of Alabama at Birmingham, Birmingham, AL 35294, USA; Birmingham VA Medical Center, Research Department, Birmingham, AL 35294, USA; Department of Medicine, Brigham and Women’s Hospital and Harvard Medical School, 77 Avenue Louis Pasteur, NRB-7, Boston, MA 02115, USA; Department of Medicine, Brigham and Women’s Hospital and Harvard Medical School, 77 Avenue Louis Pasteur, NRB-7, Boston, MA 02115, USA; Department of Medicine, Brigham and Women’s Hospital and Harvard Medical School, 77 Avenue Louis Pasteur, NRB-7, Boston, MA 02115, USA; Hainan Provincial Key Laboratory for Tropical Cardiovascular Diseases Research & Key Laboratory of Emergency and Trauma of Ministry of Education, Institute of Cardiovascular Research of the First Affiliated Hospital, Hainan Medical University, Haikou 571199, Hainan, China

**Keywords:** Eosinophil, Eosinophil cationic protein, Bone morphogenetic protein receptors, Smooth muscle cell, Calcification

## Abstract

**Aims:**

Blood eosinophil count and eosinophil cationic protein (ECP) concentration are risk factors of cardiovascular diseases. This study tested whether and how eosinophils and ECP contribute to vascular calcification and atherogenesis.

**Methods and results:**

Immunostaining revealed eosinophil accumulation in human and mouse atherosclerotic lesions. Eosinophil deficiency in *ΔdblGATA* mice slowed atherogenesis with increased lesion smooth muscle cell (SMC) content and reduced calcification. This protection in *ΔdblGATA* mice was muted when mice received donor eosinophils from wild-type (WT), *Il4^−/−^*, and *Il13^−/−^* mice or mouse eosinophil-associated-ribonuclease-1 (mEar1), a murine homologue of ECP. Eosinophils or mEar1 but not interleukin (IL) 4 or IL13 increased the calcification of SMC from WT mice but not those from Runt-related transcription factor-2 (Runx2) knockout mice. Immunoblot analyses showed that eosinophils and mEar1 activated Smad-1/5/8 but did not affect Smad-2/3 activation or expression of bone morphogenetic protein receptors (BMPR-1A/1B/2) or transforming growth factor (TGF)-β receptors (TGFBR1/2) in SMC from WT and Runx2 knockout mice. Immunoprecipitation showed that mEar1 formed immune complexes with BMPR-1A/1B but not TGFBR1/2. Immunofluorescence double-staining, ligand binding, and Scatchard plot analysis demonstrated that mEar1 bound to BMPR-1A and BMPR-1B with similar affinity. Likewise, human ECP and eosinophil-derived neurotoxin (EDN) also bound to BMPR-1A/1B on human vascular SMC and promoted SMC osteogenic differentiation. In a cohort of 5864 men from the Danish Cardiovascular Screening trial and its subpopulation of 394 participants, blood eosinophil counts and ECP levels correlated with the calcification scores of different arterial segments from coronary arteries to iliac arteries.

**Conclusion:**

Eosinophils release cationic proteins that can promote SMC calcification and atherogenesis using the BMPR-1A/1B-Smad-1/5/8-Runx2 signalling pathway.

## Structured Graphical Abstract

**Structured Graphical Abstract:**
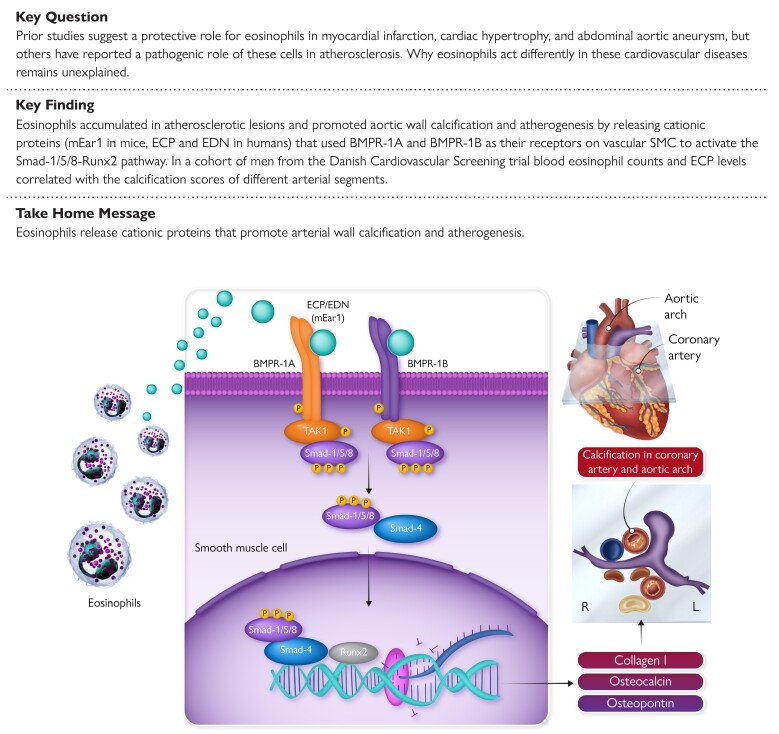
Eosinophils release cationic proteins ECP and EDN in humans (or their orthologue mEar1 in mice) that use BMPR-1A and BMPR-1B as their receptors on vascular SMC. Their ligation of these receptors activates downstream Smad-1/5/8 and Runx2 that induce the expression of osteogenic proteins as a mechanism that promotes arterial wall calcification and atherogenesis. ECP, eosinophilic cationic protein; EDN, eosinophil-derived neurotoxin; mEar1, mouse eosinophil-associated-ribonuclease-1; BMPR, bone morphogenetic protein receptor.

**See the editorial comment for this article ‘Eosinophils promote vascular calcification and atherosclerosis: adding another layer of complexity on the path to clarity?', by N. Gerdes, https://doi.org/10.1093/eurheartj/ehad323.**

**Translational perspective**

Blood eosinophils and their cationic proteins are cardiovascular disease risk factors. This study demonstrates a role for **eosinophils** and **their** cationic proteins in atherogenesis by promoting aortic smooth muscle cell osteogenic differentiation. Eosinophil cationic proteins have been recognized as risk factors of several diseases for a half-century. We find here that eosinophil cationic proteins bind to **bone morphogenetic protein receptors** to stimulate smooth muscle cell calcification. In humans, blood **eosinophil** counts and cationic protein levels associate with the calcification scores of various arterial segments. Interruption of eosinophil cationic proteins binding on their receptors may block vascular calcification and attenuate atherogenesis.

## Introduction

Eosinophils develop in the bone-marrow under the control of transcription factor GATA1.^1,2^ They are often considered effectors of allergy that reside in the respiratory tract and lymphoid organs,^3^ circulate in blood, or migrate to the site of inflammation.^4,5^ Like other granulocytes, eosinophil granules contain cytokines such as interleukin-4 (IL4), IL5, IL13, chemokines, growth factors, and a unique set of cationic proteins such as eosinophilic cationic protein (ECP) and eosinophil-derived neurotoxin (EDN) that are released upon activation^6^ and mediate cell-cell interaction.^3,6^ Eosinophil accumulation characterizes eosinophilic myocarditis patients with heart failure.^7,8^ Yet, clinical studies have yielded conflicting results regarding eosinophil involvement in cardiovascular events. In a Turkish study of 1909 ST-segment elevation myocardial infarction (STEMI) patients, the rate of in-hospital mortality and major adverse cardiac events (MACEs) were significantly higher in patients with low blood eosinophil count at admission (the percentage of eosinophil count in the peripheral blood ≤0.60%) than those with high blood eosinophil count at admission (>0.60%). Therefore, low blood eosinophil count is a biomarker for risk of STEMI patient in-hospital mortality and MACEs.^9^ A similar study of 5287 Chinese patients who underwent coronary angiography found significantly lower blood eosinophil counts in patients with non-STEMI and STEMI than in those without coronary artery diseases (CADs). Multivariable logistic regression analysis indicated a protective role for blood eosinophil counts in severe CAD [odds ratio (OR) = 0.897, *P* = 0.000] or in non-STEMI/STEMI (OR = 0.728, *P* = 0.000).^10^ However, other studies have drawn the opposite conclusion. From 3742 patients undergoing coronary angiography, blood eosinophil counts associated positively with major cardiovascular risk factors and cardiovascular disease (CVD) prevalence.^11^ In the UK Biobank, of 478 259 individuals, 1377 died of CVD and 8987 died of other causes after 7 years of follow-up. Blood eosinophil counts were significantly higher in the CVD mortality group than in those of total death (*n* = 13 482) or alive (*n* = 464 797).^12^ A sensitive marker of eosinophil activation, plasma ECP levels, was also significantly higher in patients with angiographic evidence of CAD such as stable angina and non-STEMI than those in patients with normal coronary arteries.^13^ In patients who received first generation drug-eluting stents, serum ECP levels were significantly higher in those who had MACE than in those without MACE during 18 month follow-up. Pre-intervention ECP levels predict clinical events.^14^ These clinical studies suggest a pathogenic role for eosinophils in human CVD.

In contrast to these clinical studies, several preclinical studies using eosinophil-deficient mice support a reparative role of eosinophils in the myocardium after infarct or hypertrophic injury and in abdominal aortic aneurysms (AAA). Eosinophil deficiency in *ΔdblGATA* mice increased LV dilation and cardiac hypertrophy along with enhanced cardiac dysfunction after myocardial infarction (MI) or hypertrophic injury^15–17^ and enhanced AAA growth.^18^ Yet, use of the same eosinophil-deficient *ΔdblGATA* mice yielded conflicting results regarding the role of eosinophils in atherosclerosis. In apolipoprotein E-deficient (*Apoe^−/−^*) mice, eosinophil deficiency reduced atherogenesis. Eosinophils were recruited to the lesions after aggregating with platelets, thereby enhancing lesion platelet accumulation and arterial thrombosis.^19^ In contrast, transplantation of bone-marrow from *ΔdblGATA* mice did not affect atherogenesis in low-density lipoprotein receptor-deficient (*Ldlr^−/−^*) mice.^20^ Thus, the role of eosinophils in atherosclerosis remains uncertain.

Here, we report a pathogenic role for eosinophils in atherogenesis. Eosinophils released mouse eosinophil-associated-ribonuclease-1 (mEar1), which increased vascular smooth muscle cell (SMC) apoptosis and calcification, while eosinophil-derived IL4 and IL13 did not. We found that mouse mEar1 and human ECP and EDN bound to bone morphogenetic protein receptor-1A (BMPR-1A) and BMPR-1B and activated the Smad-1/5/8-Runt-related transcription factor-2 (Runx2) signalling pathway, as a mechanism that promoted human and mouse vascular SMC calcification. A population-based study of 5864 men and a subpopulation of 394 men from the Danish Cardiovascular Screening (DANCAVAS) trial revealed positive correlations of blood eosinophil counts and ECP levels with the calcification scores in coronary artery, renal artery, iliac artery, aortic valve, mitral valve, abdominal superarenal aorta, ascending and descending aortas, and infrarenal aorta.

## Methods

### Human studies

The human population included men attending the DANCAVAS trial in Odense, Denmark. The DANCAVAS trial, which has been described in detail elsewhere,^21^ is a population-based, randomized trial of screening for subclinical CVD in men aged 65∼74. No exclusion criteria were used. One-third was invited for cardiovascular screening examinations including a CT scan at one of the four locations, among which 62.4% men attended. The screening included a low-dose, non-contrast CT scan to detect coronary artery calcification and aortic and iliac aneurysms; brachial and ankle blood pressure index to detect peripheral arterial disease and hypertension; a telemetric assessment of heart rhythm; and a measurement of blood cholesterol and glucose levels. For this study, leucocyte counts, including eosinophils, were measured by flow cytometry on a Sysmex XN-9000 in the clinic from 5864 consecutive men who gave blood samples from January 2015 to August 2018. Of these 5864 men, a random subpopulation of 3494 men was divided into two groups with the highest and lowest CAC scores. From each group, we selected 200 participants and measured their plasma ECP levels using a high sensitivity human ECP ELISA kit (SK00128-06, Aviscera Bioscience, Santa Clara, CA). The investigators measuring the ECP levels were blinded to the CAC scores. The samples between the two groups were analyzed in a random order on the ELISA kit. Six samples were excluded due to incomplete information, leading to an ECP subpopulation of 394 individuals. The calcification score of the coronary artery, aortic segment, renal artery, and iliac artery was calculated using the Agatston Method^22^ with a SyngoVia (Siemens Healthcare Solutions) workstation and seven trained radiographers, blinded to clinical data. At attendance of screening, each man was first given the informed consent and then a medical history was obtained including previous acute MI, coronary revascularization, chronic obstructive pulmonary disease (COPD), medication history, and symptoms. Up-to-date cardiovascular preventive treatment was recommended in case of subclinical cardiovascular disease. Use of anonymized patient information from the DANCAVAS was approved by the Human Investigation Review Committee at the Brigham and Women’s Hospital, Boston, MA, USA (protocol #2010P001930) with a waiver of informed consent since the study did not involve patient contact or enrolment.

### Mice and atherosclerotic lesion characterization

*ΔdblGATA* (C57BL/6, N12), *Apoe*^−/−^ (C57BL/6, N12), *Il4*^−/−^ (C57BL/6, N12), and *Myh11^CreER(T)^* (C57BL/6, N > 7) mice were obtained from the Jackson laboratory (Bar Harbor, ME). *Il13*^−/−^ and *BMPR-1A^fl/fl^* mice were reported previously.^18,23^*BMPR-1B^fl/fl^* (C57BL/6J, N12) mice were produced from Cyagen Biosciences Inc. (Santa Clara, CA). *ΔdblGATA* and *Apoe*^−/−^ mice were crossbred to generate the *Apoe*^−/−^*ΔdblGATA* and *Apoe*^−/−^ littermates. *BMPR-1A^fl/fl^* and/or *BMPR-1B^fl/fl^* mice were crossbred with *Myh11^CreER(T)^* mice to generate *Myh11^CreER(T)^BMPR-1A^fl/fl^*, *Myh11^CreER(T)^BMPR-1B^fl/fl^*, and *Myh11^CreER(T)^BMPR-1A^fl/fl^/1B^fl/fl^* mice and their littermate *BMPR-1A^fl/fl^*, *BMPR-1B^fl/fl^*, and *BMPR-1A^fl/fl^*/*1B^fl/fl^* mice. To induce atherosclerotic calcification, 6-week-old male *Apoe*^−/−^*ΔdblGAT*A and *Apoe*^−/−^ control mice were fed with an atherogenic diet (D12108c, Research Diet, New Brunswich, NJ) for 12 weeks. To determine the role of eosinophils in mouse atherosclerotic calcification, eosinophil-deficient *Apoe*^−/−^*ΔdblGATA* mice received adoptive transfer of *in vitro* cultured eosinophils from C57BL/6 WT mice, followed by the consumption of an atherogenic diet. Our recent study showed that donor eosinophils remained high for 7 days after adoptive transfer in recipient mouse hearts but lowered after 14 days.^17^ Therefore, each recipient mouse received six doses of 1 × 10^7^ donor eosinophils by intravenous (i.v.) injection starting at day 0 before being fed an atherogenic diet and two weeks thereafter. To test the role of eosinophil-derived IL4 and IL13 and mEar1 in atherosclerosis and aortic calcification, we performed adoptive transfer of eosinophils from *Il4*^−/−^ and *Il13*^−/−^ mice to *Apoe*^−/−^*ΔdblGATA*-recipient mice by six doses of i.v. injection of 1 × 10^7^ donor eosinophils or subcutaneous (s.c.) injection of mEar1 twice per week (5 μg/mouse/time, 00128-03, Aviscera Bioscience) or phosphate-buffered saline (PBS) as controls. A total of 9∼11 mice per experimental group reached to the 12-week time point. All mice were sacrificed with carbon dioxide narcosis, followed by cardiac puncture blood collection.

We embedded the aortic root and aortic arch in optimal cutting temperature (OCT) compound (23730571, Thermo Fisher Scientific, Hampton, NH) after harvesting the heart and aorta. We prepared 10 slides per block, each of which contained three 6-μm frozen serial sections through the aortic sinus with all three valve leaflets visible, aortic arch with all three branches (left subclavian artery, left common carotid artery, and brachiocephalic artery) visible. The lesions in the root of the aorta beneath all three-valve leaflets near the ostia of the coronary arteries were analyzed. The lesions in the aortic arches were analyzed as we reported previously.^24^ To characterize atherosclerotic lesion composition, serial cryostat cross-sections were used for immunostaining to detect SMC (α-actin, 1:750, F3777, Sigma-Aldrich, St. Louis, MO), macrophages (Mac-3, 1:900, 553322, BD Biosciences, Billerica, MA), CD4^+^ T-cells (CD4, 1:90; 553043, BD Biosciences), CD31^+^ mircovessels (CD31, 1:1500, 553370, BD Biosciences), MHC class-II (MHC-II) (antigen-presenting cell marker, 1:150, 556999, clone M5/114.15.2, BD Biosciences), elastin (Modified Verhoeff Van Gieson Elastic Stain Kit, HT25A, Sigma-Aldrich), and collagen (0.1% Sirius Red F3BA; 09400, Polysciences Inc., Warrington, PA). Lesion apoptotic cells were determined with the in-situ apoptosis detection kit, according to the manufacturer’s instructions (S7100, Sigma-Aldrich). Collagen content and elastin fragmentation were graded according to the grading keys described previously.^25^ CD4^+^ T-cells, CD31^+^ microvessels and apoptotic-positive cells were counted blindly and quantified as numbers per aortic section. Serial cryostat cross-sections were used for image analysis using an inverted Nikon Eclipse TE2000-U microscope. The relative SMC, macrophage, and MHC-II-positive cell contents within the aortas were quantified by measuring the immunostaining signal positive area using computer-assisted image analysis software (Image-Pro Plus; Media Cybernetics, Bethesda, MD). Investigators were blinded to the sources of samples, the assay, and quantification.

### Mouse aortic tissue and cell calcification characterization

The deposition of mineralized calcium in mouse aortic tissues and *in vitro* cultured SMC was determined by the Alizarin red S (A5533, Sigma-Aldrich) staining. Cryostat sections of aortic tissues or human and mouse SMC were fixed with 4% paraformaldehyde (PFA) for 5 min, rinsed for 5 min, stained with 1% Alizarin red S solution (pH 4.2) for 5 min, rinsed by three changes of PBS (pH 7.4) to remove non-specific staining, dehydrated with acetone, acetone/xylene (1:1), and xylene, and slides were mounted with Permanent Mounting Medium (H-5000, Vector Laboratories, Burlingame, CA). Images were captured and calcium contents were quantified by measuring the Alizarin red-positive area using Image-Pro Plus.

### Alkaline phosphatase activity detection

The alkaline phosphatase (ALP) activity in mouse aortic roots and arches were determined using Vector® Red Substrate Kit, ALP (SK-5100, Vector Laboratories) according to the manufacturers’ instructions. Briefly, cryostat sections of aortic roots and arches were rinsed with PBS and stained with an appropriate amount of substrate-working solution at room temperature in the dark for 30 min. Slides were then rinsed with PBS to remove non-specific staining, and the stained matrix was photographed. The ALP activity in mouse aortic tissues was quantified by measuring the positive area using Image-Pro Plus.

Intracellular ALP activity was detected using an ALP activity colorimetric assay kit (K412, BioVision Inc., Mountain View, CA). After osteogenic induction and treatments, the original medium was removed. The cells were added with 50 µL of assay buffer to homogenize and centrifuged at 13 000 g for 3 min to remove insoluble materials. Samples were plated to a 96-well plate, added with 50 µL of 5 mM p-nitrophenyl phosphate solution in each well, and incubated at room temperature in the dark for 60 min. The ALP activity was calculated based on the standard curve and normalized to the total cellular protein content: ALP activity (U/g protein) =A (amount of p-nitrophenyl produced by each sample, μmol)/V (volume of the reaction system, mL)/T (time of reaction, min)/lysate protein concentration (g/mL).

### Mouse aortic tissue and cell immunofluorescence staining

To characterize osteogenesis, we performed immunofluorescence triple-staining on acetone-fixed cryosections from mouse atherosclerotic lesions and mouse SMC cultured in eight-chamber culture slide (08-774-26, Thermo Fisher Scientific). Slides were blocked with PBS containing 5% BSA for 1 h at room temperature and then incubated with the following antibodies: mouse α-actin-FITC antibody (1:100, F3777, Sigma-Aldrich), rabbit osteocalcin antibody (1:100, AB10911, Sigma-Aldrich), and rat Runx2 antibody (1:100, 692802, BioLegend, San Diego, CA). The secondary antibodies were Alexa Fluor 555 (1:500, A21432, Thermo Fisher Scientific) or Alexa Fluor 647 (1:300, A21244, Thermo Fisher Scientific). The nuclei were counterstained with DAPI (1:10, R37606, Thermo Fisher Scientific). Slides were mounted using Fluorescence Mounting Medium (S3023, Agilent/Dako, Santa Clara, CA), and images were collected under an Olympus FluoView™ FV1000 confocal microscope. α-Actin, osteocalcin and nuclear Runx2 were quantified by measuring the immunostaining signal-positive or double-stained area using Image-Pro Plus. Ki67 or cleaved caspase-3-positive SMCs were determined by immunofluorescence double-staining with rat monoclonal Ki67 antibody (1:100, 14-5698-82, Thermo Fisher Scientific) or rabbit monoclonal cleaved caspase-3 antibody (Alexa Fluor(R) 594 Conjugate, 1:100, 8172S, Cell Signaling Technology, Danvers, MA), together with mouse monoclonal α-actin-FITC antibody (1:100, F3777, Sigma-Aldrich). Secondary antibody for anti-Ki67 antibody was Alexa Fluor 555 (1:500, A21432, Thermo Fisher Scientific). Ki67 or cleaved caspase-3-positive SMCs in arterial wall were quantified by measuring the double-stained area using Image-Pro Plus. Cleaved caspase-3- and osteopontin-positive SMCs were determined by immunofluorescence triple-staining with rabbit monoclonal cleaved caspase-3 antibody [Alexa Fluor(R) 594 Conjugate, 1:100, 8172S, Cell Signaling Technology], goat polyclonal osteopontin antibody (1:100, MBS421341, MyBioSource, San Diego, CA), together with mouse monoclonal α-actin-FITC antibody (1:100, F3777, Sigma-Aldrich). Secondary antibody for osteopontin antibody was Alexa Fluor 647 (1:500, A21447, Thermo Fisher Scientific). Cleaved caspase-3- and osteopontin-positive SMC in arterial wall were quantified by measuring the triple-stained area using Image-Pro Plus. Cleaved caspase-3-positive macrophages were determined by immunofluorescence double-staining with rabbit monoclonal cleaved caspase-3 antibody [Alexa Fluor(R) 594 Conjugate, 1:100, 8172S, Cell Signaling Technology], together with mouse monoclonal CD68 antibody (1:100, ab955, Abcam, Waltham, MA). Secondary antibody for CD68 antibody was Alexa Fluor 488 (1:500, A11029, Thermo Fisher Scientific). Cleaved caspase-3-positive macrophages in arterial wall were quantified by measuring the double-stained area using Image-Pro Plus. Human atherosclerotic lesions were double stained with rabbit monoclonal tryptase antibody (1:100, 51550S Cell Signaling Technology) or rat monoclonal CD68 antibody (1:100, 137002, BioLegend), together with mouse monoclonal Siglec-8 antibody (1:100, 347102, BioLegend). Secondary antibody for tryptase, CD68, or Siglec-8 antibodies were Alexa Fluor 488 (1:500, A11008), Alexa Fluor 488 (1:500, A11006) or Alexa Fluor 555 (1:500, A21422), all from Thermo Fisher Scientific. Mouse atherosclerotic lesions were double stained with rabbit polyclonal mouse mast cell protease-4 (MMCP4) antibody (1:200)^26^ or mouse monoclonal CD68 antibody (1:100, ab955, Abcam), together with rat monoclonal Siglec-F antibody (1:100, 552125, BD Pharmingen, San Diego, CA). Secondary antibody for MMCP4, CD68, or Siglec-F antibodies were Alexa Fluor 488 (1:500, A11008), Alexa Fluor 488 (1:500, A11029), or Alexa Fluor 555 (1:500, A21434), all from Thermo Fisher Scientific. Aortic root lesions from *Apoe^−/−^ΔdblGATA* mice received adoptive transfer of eosinophils from CD45.1 transgenic mice were double stained with rabbit polyclonal mEar1 antibody (1:100, orb13385, Biorbyt, St Louis, MO) or mouse monoclonal CD45.1 antibody (1:100, 1795-10, SouthernBiotech, Birmingham, AL), together with rat monoclonal Siglec-F antibody (1:100, 552125, BD Pharmingen). Secondary antibody for mEar1, CD45.1 or Siglec-F antibodies were Alexa Fluor 555 (1:500, A21428), Alexa Fluor 488 (1:500, A11029), Alexa Fluor 488 (1:500, A11006), or Alexa Fluor 555 (1:500, A21434), all from Thermo Fisher Scientific.

### Mouse plasma lipoprotein and BMP measurement

Blood samples were collected at harvest from *Apoe^−/−^*, *Apoe^−/−^ΔdblGATA*, and *Apoe^−/−^ΔdblGATA* mice treated with mEar1 or received adoptive transfer of eosinophils from WT, *Il4^−/−^*, and *Il13^−/−^* mice. Plasma samples were stored at −80°C until analysis. Plasma total cholesterol (23-666-200, Pointe Scientific, Canton, MI), triglyceride (23-666-410, Pointe Scientific), and HDL (23-666-301, Pointe Scientific) levels were determined using the enzymatic methods according to the manufacturer. The LDL cholesterol level was determined using the Friedewald formula: Plasma LDL cholesterol concentration (mg/dL) = total cholesterol−HDL cholesterol−(triglycerides/5). Plasma BMP-2 (MBS262739, MyBioSource, San Diego, CA), BMP-4 (MBS260174, MyBioSource), mEar1 (SK00128-03, Aviscera Bioscience), leptin (RD291001200R, Biovendor, Asheville, NC), IL-1β, IL-6, TNF-α, IFN-γ (88-7013, 88-7064, BMS607-3TWO, 88-7314, Thermo Fisher Scientific) levels were determined using ELISA kits according to the manufacturers’ instructions.

### Cell culture

Eosinophils were cultured as previously reported.^17,18^ Briefly, bone-marrow cells were collected from mouse femurs and tibias, centrifuged for 5 min at 300 g, followed by lysing red blood cells (RBCs) by pipetting cell pellet up and down twice in 9 mL sterile H_2_O and then immediately adding 1 mL of 10 × PBS. Once RBCs were lysed, cells were suspended in 10 mL 1 × PBS and counted. Cells with a concentration of 10^6^ cells/mL were plated in an 80 cm^2^ flask and cultured in a base media containing mouse stem cell factor (SCF, 100 ng/mL, 250-03, PeproTech, Rocky Hill, NJ) and Flt-3-ligand (Flt3-L, 100 ng/mL, 250-31L, PeproTech) for 2 days. On day 2, half of the media was replaced with fresh medium containing SCF and Flt3-L. On day 4, culture media was replaced with fresh media containing IL5 (10 ng/mL, 405-ML, R&D Systems, Minneapolis, MN). Half of the culture media were changed every 2 days with fresh media containing IL5 until the 14th days. On day 14, cells were collected for the further study. FACS monitored eosinophil purity and expression of eosinophil surface markers (Siglec-F and CD45.1), as we recently described.^16^

Mouse aortic SMCs were isolated from aortas of WT, Runx2*^f/f^* and Runx2*^f/f^*; SM22α-Cre mice.^24,27^ Briefly, mouse abdominal aortas were removed and separated from fat and adventitia followed by digestion with collagenase type II (2 mg/mL, Worthington Biochemical Corp., Lakewood, NJ) at 37°C for 10 min. The adventitia was completely stripped under microscopic visualization, the aortas were opened, and EC were removed by abrasion. The aortas were then chopped and digested in type II collagenase (1 mg/mL; Worthington Biochemical Corp.) for 40–50 min at 37°C. Digestion was stopped by adding 10 mL of Dulbecco's Modified Eagle Medium (DMEM) containing 10% foetal bovine serum (FBS). Cells were resuspended by gentle pipetting, passed through a 70 μm filter, and spun at 300 g for 10 min at 4°C. Cells were cultured in DMEM (11995-065, Gibco, Waltham, MA) supplemented with 10% (vol/vol) FBS and GlutaMAX (4.5 g/L, 35050061, Thermo Fisher Scientific). SMCs were plated onto Corning 60 mm dish. After 2 passages SMCs were transferred onto six-well plates and allowed to grow until they were ready for treatment. Aortic SMCs were isolated from *Myh11^CreER(T)^BMPR-1A^fl/fl^*, *Myh11^CreER(T)^BMPR-1B^fl/fl^*, and *Myh11^CreER(T)^BMPR-1A^fl/fl^/1B^fl/fl^* mice and *BMPR-1A^fl/fl^*, *BMPR-1B^fl/fl^*, and *BMPR-1A^fl/fl^*/*1B^fl/fl^* control mice. Cells were treated with 0.5 μM of tamoxifen or vehicle (dimethyl sulfoxide, DMSO) for 24 h to induce Cre-mediated recombination.

Human aortic SMCs were isolated and cultured by explant outgrowth from unused portions of non-atherosclerotic carotid arteries from heart transplant donors, as previously described.^28^ Cells were grown in DMEM supplemented with 10% FBS, 100 U/mL penicillin, 100 μg/mL streptomycin, 1.25 μg/mL amphotericin B, and 2 mmol/L l-glutamine. Cells were passaged by brief trypsinization and used through passage 5 for the experiments after being growth-arrested for 2 days in serum-free insulin–transferrin medium consisting of DMEM and Ham’s F12 (1:1, vol/vol; 11320-033, Thermo Fisher Scientific) supplemented with 1 μmol/L insulin and 5 μg/mL transferrin. Fresh insulin–transferrin medium was used for the experiments with or without addition of stimuli.

### SMC osteogenic differentiation induction and treatment

Human and mouse SMC were plated in 24-well plates with 2 × 10^4^ cells in each well until reaching 60%–70% confluence. Cells were exposed to osteogenic media containing 2.5 mM CaCl_2_ and 5 mM β-glycerophosphate by continuous culture in medium for 14 days. Osteogenic media was refreshed every 2 days. Human SMC were treated with 500 ng/mL ECP (00128-01-100, Aviscera Bioscience) or EDN (H00006036-Q01, Abnova, Walnut, CA). Control and Runx2 knockout aortic SMC were exposed to osteogenic media with or without mEar1 (500 ng/mL), murine IL4 (100 ng/mL, 214-14, PeproTech) or IL13 (100 ng/mL, 210-13, PeproTech), eosinophil lysate from WT, *Il4^−/−^* or *Il13^−/−^* mice at 10^6^ eosinophils/mL, and WT eosinophil lysate plus mEar1 antibody (1000 ng/mL, orb13385, Biorbyt). The treatment was refreshed along with the changes of osteogenic media. At the 14th day, the cells were stimulated by the respective treatment for 30 min before collection for further study. To test the effect of calcification on SMC apoptosis, WT SMCs were cultured in control media or osteogenic media and treated with or without 20 μM of PDTC.

### Real-time PCR

Total RNA from human and mouse SMC was prepared using the TRIzol™ reagent (15596018, Thermo Fisher Scientific), and cDNA was reverse transcribed using the high-capacity cDNA reverse transcription kit (4368813, Thermo Fisher Scientific). The relative mRNA levels of target genes were quantified using the iTaq UniverSYBR Green SMX 5000 (1725125, Bio-Rad, Hercules, CA) with an ABI PRISM 7900 sequence detector system (Applied Biosystems Co, Foster City, CA). Each reaction was performed in duplicate, and changes in relative gene expression levels were normalized to glyceraldehyde 3-phosphate dehydrogenase (GAPDH) levels using the relative threshold cycle method. Primer sequences are listed in Supplementary data online, *Table S3*.

### Immunoblot analysis

Human and mouse SMCs were harvested and lysed in a lysis buffer containing 50 mM Tris–HCl (pH 7.6), 150 mM NaCl, 1% NP-40, 0.5% sodium deoxycholate, and 0.1% SDS. Equal amounts of protein from each cell-type preparation were separated on a SDS–PAGE, blotted, and detected with rabbit anti-ALP (1:1000, NBP2-67295, Novus Biologicals, Littleton, CO), goat anti-BMPR-1A (1:1000, PA5-18494, Thermo Fisher Scientific), mouse anti-BMPR-1B (1:1000, MAB505-100, R&D Systems), mouse anti-BMPR-2 (1:1000, MA5-15827, Thermo Fisher Scientific), mouse anti-collagen I (1:1000, PA5-29569, Thermo Fisher Scientific), rabbit anti-osteocalcin (1:1000, AB10911, Sigma-Aldrich), mouse anti-osteopontin (1:1000, 691302, BioLegend), rat anti-Runx2 (1:1000, 692802, BioLegend), rat anti-TGFBR-1 (1:1000, MAB5871, R&D Systems), and rat anti-TGFBR2 (1:1000, MAB532, R&D Systems), and GAPDH (1:2000, 2118S, Cell Signaling Technology) antibodies. Western blot signals were assessed by densitometric analysis using the Image J software.

### Nuclear and cytoplasmic extract preparation

The nuclear extraction was prepared using an NE-PER nuclear cytoplasmic extraction reagent kit (78833, Thermo Fisher Scientific). Briefly, human and mouse SMC cultured on a six-well plate were exposed to the above-mentioned treatment and then washed three times with PBS. The cultured cells were lysed in RIPA buffer (50 mM Tris-HCL pH 7.4, 150 mM NaCl, 1% NP-40, 0.5% sodium deoxycholate, and 0.1% SDS) supplemented with complete protease inhibitor cocktail tablets (04906845001, Thermo Fisher Scientific). Cell debris was removed by centrifugation at 12 000 rpm for 20 min. Then cells were suspended in 200 μL of cytoplasmic extraction reagent-I and incubated on ice for 10 min followed by addition of 11 μL of a second cytoplasmic extraction reagent-II. After centrifugation, the supernatant fraction (cytoplasmic extract) was transferred to a pre-chilled tube. The insoluble pellet fraction, which contains crude nuclei, was re-suspended in 100 μL of a nuclear extraction reagent. After centrifugation, the resulting supernatant, constituting the nuclear extract, was used for immunoblotting to detect Runx2 (1:1000, 692802, BioLegend) and histone-H3 (1:1000, 9715S, Cell Signaling Technology).

### Overexpression, transient knockdown and transfection

The mammalian expression vector pcDNA3.0 encoding the BMPR-1A (ID 80873), BMPR-1B (ID 80882), and BMPR-2 (ID 163403) cDNAs were obtained from Addgene (Watertown, MA) to overexpress the BMPRs in 293 T cells. BMPR-1A (ID s281) and BMPR-1B (ID s2042) siRNA was obtained from Invitrogen (Carlsbad, CA) to knockdown the expression of these receptors in human SMC. Transfections of the cDNA and siRNA were performed using Lipofectamine® 2000 Reagent (11668-027, Invitrogen) according to manufacturer’s protocol. The cells were incubated in cDNA/siRNA-lipid complexes for 24 h at 37°C. After 24 h, the media was changed to fresh media and the transfected cells were used for further experiments.

### ECP and mEar1 BMPR binding assay and Scatchard plot analysis

Recombinant mouse mEar1, and human ECP and EDN were conjugated with FITC using the FluoroTag™ FITC Conjugation Kit (FITC1-1KT, Sigma-Aldrich). Briefly, mEar1, ECP, or EDN was diluted at 5.0 mg/mL in 0.1 M carbonate-bicarbonate buffer, pH 9.0, and combined with 50 μL FITC solution with different dilutions (5:1, 10:1, 20:1), according to the manufacturer’s instructions. Reaction was carried at room temperature under dark for 2 h. FITC-conjugated mEar1, ECP, or EDN was purified over a Sephadex G-25 M column. Binding assay was performed according to an established protocol as previously described.^29^ In brief, SMC and 293 T cells were resuspended in a buffer containing 20 mM HEPES and 10 mM EDTA, pH 7.5 and incubated at 4°C for 15 min. Cells were then homogenized and centrifuged at 28 000 rpm at 4°C for 30 min. Cell pellet was resuspended into a storage buffer containing 20 mM HEPES and 1 mM EDTA, pH 7.5, and re-homogenized with polytron PT3100 homogenizer. An equal amount of protein was incubated with FITC-mEar1, FITC-ECP or FITC-EDN in a binding buffer (50 mM Tris-HCL, 5 mM MgCl_2_, 25 μM EDTA, 0.2% BSA) for 90 min at room temperature in an AcroWell™ 96 Filter Plate (Pall Corp., Port Washington, NY). The plate was then washed four times with 50 mM Tris-HCl and 5 mM MgCl_2_. The plate was read at 494 nm excitation and 518 nm emission. Scatchard plot analysis was performed using the Prism 9.0 software (GraphPad Software Inc., La Jolla, CA). To test FITC-mEar1, FITC-ECP or FITC-EDN binding affinity on SMC, the cells post osteogenic differentiation were transfected with BMPR-1A and/or BMPR-1B siRNA and treated with or without excessive BMP2 (1000 ng/mL, 767304, BioLegend). To test FITC-mEar1 binding affinity on BMPRs from 293 T cells, the cells were transfected with BMPR expression vectors and treated with or without excessive mEar1 (5 µg/mL) or BMP2 (1 µg/mL). Empty vector-transfected 293 T cells were used as background controls. Data were presented as the mean of three experiments.

### Immunoprecipitation

Human and mouse SMCs were lysed in an immunoprecipitation lysis buffer (0.025 M Tris, 0.15 M NaCl, 0.001 M EDTA, 1% NP-40, 5% glycerol; pH 7.4) and pre-cleared with control agarose resin for 1 h according to the manufacturer’s instructions (26149, Thermo Fisher Scientific). Cell lysates (500 μg) were subsequently incubated overnight at 4°C with either rabbit anti-human ECP (10 μg, orb156688, Biorbyt), rabbit anti-human EDN (10 μg, NBP3-03635, Novus Biologicals), rabbit anti-mouse mEar1 (10 μg, orb13385, Biorbyt), or mouse IgG isotype (10 μg, 026502, Thermo Fisher Scientific) antibodies. After captured, washed and eluted, the immunoprecipitates were separated on a 10% SDS–PAGE, immunoblotted with goat anti-mouse BMPR1A (1:1000), mouse anti-BMPR-1B (1:1000), mouse anti-BMPR-2 (1:1000), rat anti-TGFBR-1 (1:1000), and rat anti-TGFBR2 (1:1000) antibodies, to detect the immune complexes. Likewise, cell lysates were incubated with goat anti-BMPR-1A (10 μg) or mouse anti-BMPR-1B (10 μg) antibodies. The immunoprecipitates were separated and immunoblotted with rabbit anti-human ECP (1:1000) or rabbit anti-human EDN (1:1000) antibodies. The same blot was also probed with a horseradish peroxidase (HRP)-conjugated goat anti-rabbit IgG (1:3000, A16104, Thermo Fisher Scientific), rabbit anti-goat IgG (1:3000, A16136, Thermo Fisher Scientific), and goat anti-mouse IgG (1:3000, 31430, Thermo Fisher Scientific) to detect immunoprecipitated IgG from each sample.

### Statistical analysis

Blood eosinophil counts and calcification scores were poorly normally distributed. Therefore, non-parametric test was performed for univariate analyses. Median and interquartile range (IQR) were given as descriptive statistics, and baseline characteristics were compared by χ^2^, Wilcoxon rank sum test, or Kruskal–Wallis test. Associations with a *P*-value below 0.100 were considered to be potential confounders. Spearman’s correlation analysis was used to assess the correlation between eosinophil counts and calcification score, and Kruskal–Wallis analysis was used for the quartiles of calcification scores and the eosinophil counts. Finally, multivariate test was performed after log-transformation of blood eosinophil counts and calcification scores. Partial correlation analyses were performed after adjusting for the potential confounders identified above. All mouse data were expressed as mean ± standard error of means (SEM). Shapiro–Wilk test was used to determine data distribution normality. One-way analysis of variance test followed by a *post hoc* Tukey’s test was used for multiple comparisons (≥3 groups) with normally distributed variables. The Kruskal–Wallis test followed by Dunn’s procedure was conducted for multiple comparisons with abnormally distributed variables. SPSS 20.0 and Prism 7 (GraphPad) software were used for statistical analysis. SPSS28 0.1.0 version was used for analysis and *P* < 0.05 was considered statistically significant.

### Study approvals

Discarded and decoded human aortas and patient data were reused according to the protocol #2010P001930 pre-proved by the Human Investigation Review Committee at the Brigham and Women’s Hospital, Boston, MA, USA. No patient informed consent was required. All animal procedures conformed to the Guide for the Care and Use of Laboratory Animals published by the US National Institutes of Health and was approved by the Brigham and Women's Hospital Standing Committee on Animals (protocol #2016N000442).

## Results

### Eosinophil accumulate in human and murine atherosclerotic lesions

Immunostaining using the antibody against human sialic-acid-binding immunoglobulin-like lectin (Siglec-8), an inhibitory receptor selectively expressed on human eosinophils,^30^ detected no eosinophils in normal human aorta, but accumulation of Siglec-8-positive eosinophils in human atherosclerotic lesions (see Supplementary data online, *Figure S1A*). Studies of mouse atherosclerotic lesions yielded similar results. Frozen sections of aortic roots from *Apoe^−/−^* mice that consumed an atherogenic diet or a chow diet (normal) for 12 weeks were used for immunostaining with mouse eosinophil-specific Siglec-F antibody. The normal mouse aortic root showed negligible Siglec-F staining, but Siglec-F-positive eosinophils accumulated in mouse atherosclerotic arteries, mostly in the adventitia (see Supplementary data online, *Figure S1B*). To exclude the possibility that mast cells or macrophages express Siglec-8 or Siglec-F, we performed immunofluorescence double-staining with Siglec-8 antibody and human mast cell tryptase or macrophage CD68 antibodies in human normal aorta and atherosclerotic arteries, and used Siglec-F antibody together with mouse mast cell chymase (MMCP4)^26^ and CD68 antibodies for mouse normal aorta and atherosclerotic aorta. As expected, normal human or mouse aortas did not contain mast cells or macrophages. Tryptase- or chymase-positive mast cells and CD68-positive macrophages accumulated in human and mouse atherosclerotic lesions. These cells did not express Siglec-8 or Siglec-F (see Supplementary data online, *Figure S2A–D*). ECP and mEar1 (mouse eosinophil-associated-ribonuclease-1, a murine homologue of ECP) antibodies were used to confirm human and mouse eosinophil accumulation. All Siglec-8-positive cells in human atherosclerotic lesions and Siglec-F-positive cells in mouse atherosclerotic lesions expressed ECP and mEar1 (see Supplementary data online, *Figure S2E* and *F*).

### Eosinophil deficiency protects mice from atherosclerosis

**Figure 1:**
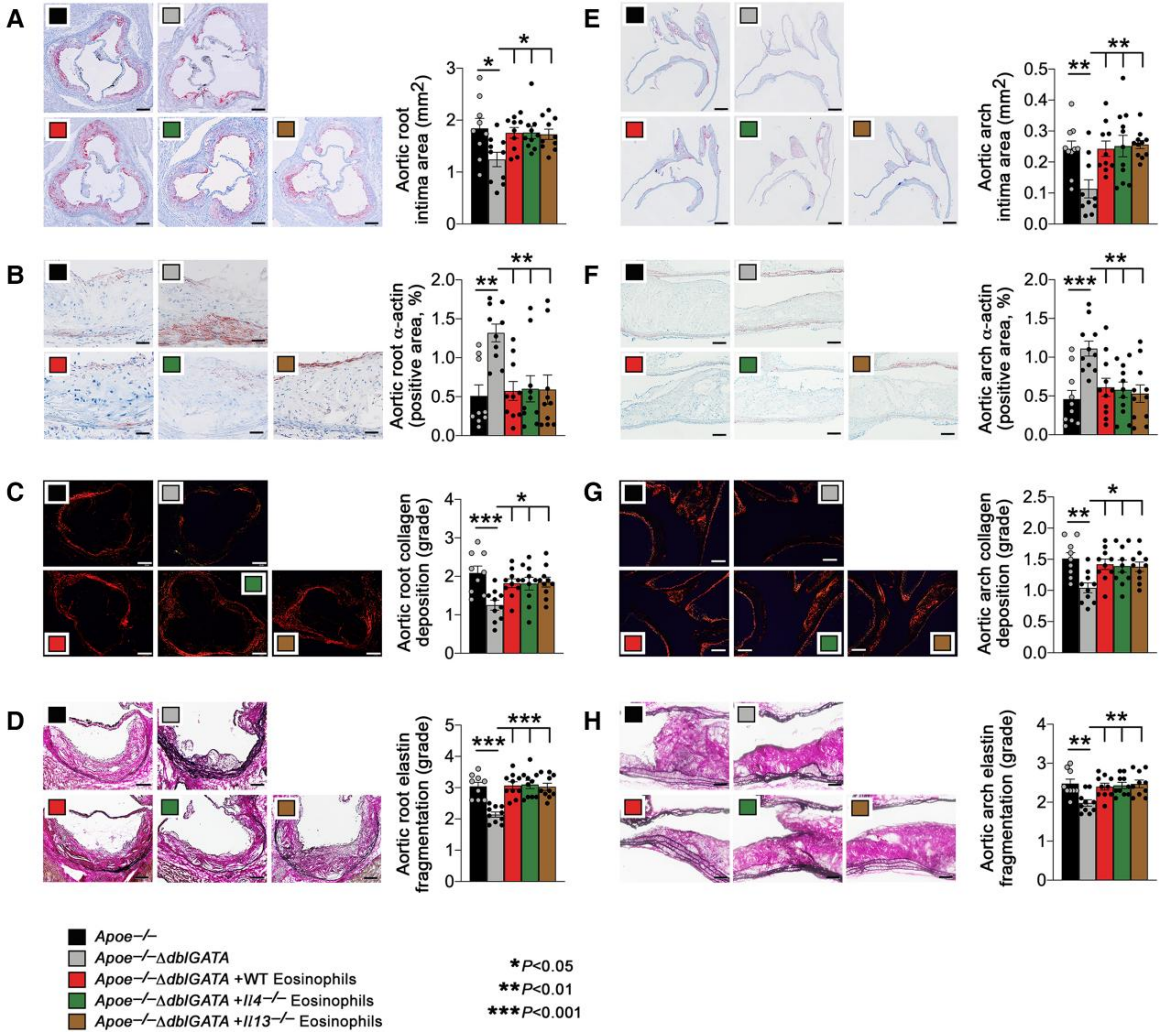
Eosinophils accelerate atherogenesis in *apoe^−/−^* mice. *Apoe^−/−^* and *Apoe^−/−^ΔdblGATA* mice were fed with an atherosclerotic diet for 12 weeks. *Apoe^−/−^ΔdblGATA* mice were also reconstituted with eosinophils from wild-type, *Il4^−/−^*, or *Il13^−/−^* mice every 2 weeks. Aortic root intima area, scale: 500 μm (*A*), α-actin^+^ smooth muscle cell contents in arterial wall, scale: 200 μm (*B*), Sirius red collagen staining in grade, scale: 200 μm (*C*), and elastin fragmentation in grade, scale: 200 μm (*D*). Aortic arch intima area, scale: 500 μm (*E*), α-actin^+^ smooth muscle cell contents in arterial wall, scale: 200 μm (*F*), Sirius red collagen staining in grade, scale: 200 μm (*G*), and elastin fragmentation in grade, scale: 200 μm (*H*). The results are expressed as mean ± standard error of mean of 9–10 mice per group.

**Figure 2:**
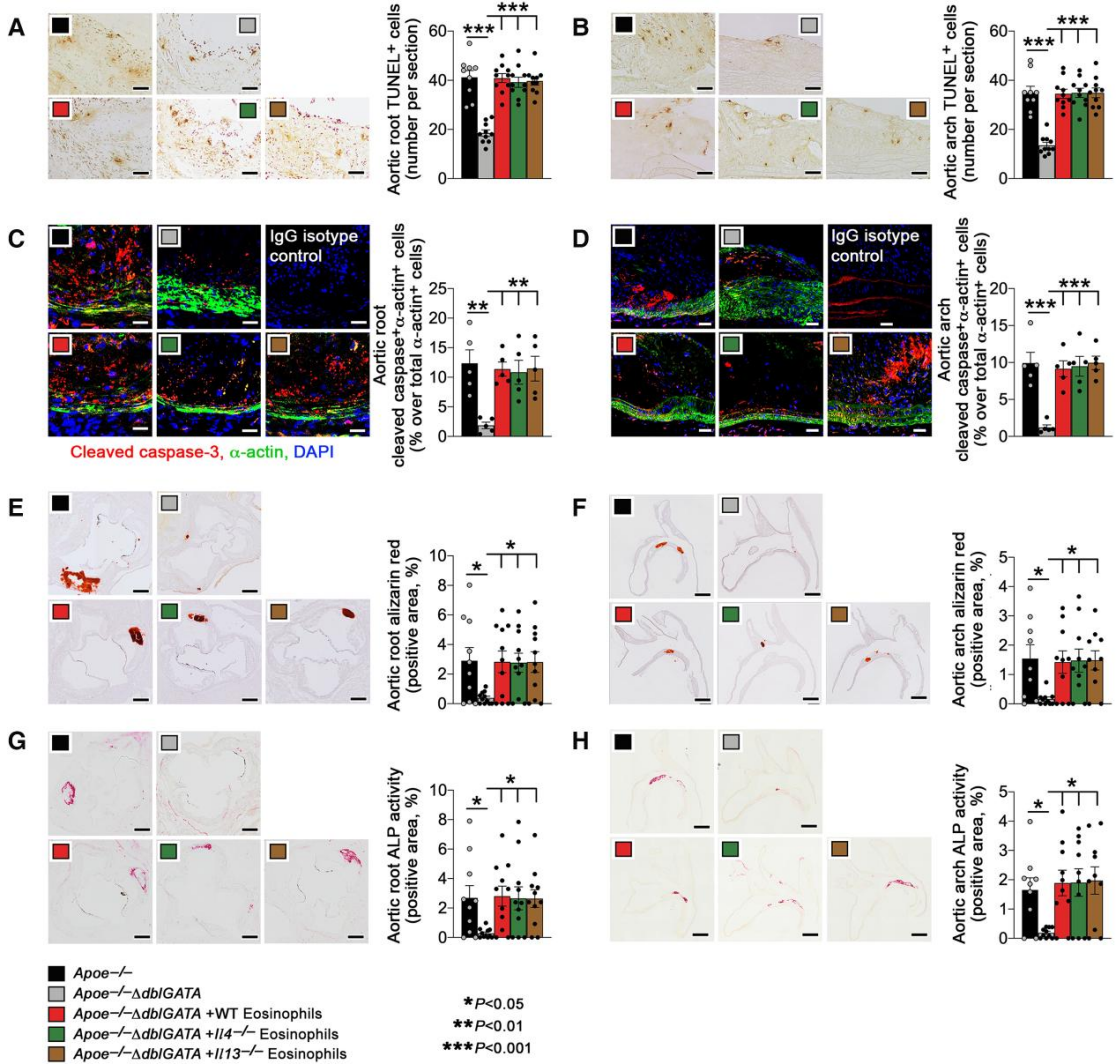
Eosinophils promote arterial calcification and lesion apoptosis in *apoe^−/−^* mice. *Apoe^−/−^* and *Apoe^−/−^ΔdblGATA* mice were fed with an atherosclerotic diet for 12 weeks. *Apoe^−/−^ΔdblGATA* mice were reconstituted with eosinophils from WT, *Il4^−/−^*, or *Il13^−/−^* mice every 2 weeks. (*A*) Aortic root and (*B*) arch TUNEL-positive apoptotic cell contents. Scale: 100 μm. (*C*) Root and (*D*) arch immunofluorescence staining for cleaved caspase-3 ^+^ α-actin^+^ smooth muscle cell. IgG isotypes were used as experimental controls. Scale: 50 μm. (*E*) Root and (*F*) arch Alizarin red-positive calcium deposition. Scale: 500 μm. (*G*) Root and (*H*) arch alkaline phosphatase activity-positive area. Scale: 500 μm. The results are expressed as mean ± standard error of mean of 9–10 mice per group.

To test the participation of eosinophils in atherogenesis, we produced eosinophil-deficient *Apoe^−/−^ΔdblGATA* mice and fed both *Apoe^−/−^* and *Apoe^−/−^ΔdblGATA* mice an atherogenic diet for 12 weeks. Compared with the *Apoe^−/−^* mice, *Apoe^−/−^ΔdblGATA* mice showed significantly reduced atherosclerotic lesion size, increased lesion α-actin-positive SMC content, and reduced lesion collagen accumulation and elastica fragmentation in both the aortic root (*Figure 1A–D*) and aortic arch (*Figure 1E–H*). Our earlier studies demonstrated a cardiovascular reparative role for eosinophil-derived IL4 and IL13 in myocardial infarcted hearts, pressure overload-induced cardiac hypertrophy, and angiotensin-II infusion-induced AAA.^16–18^ Yet, adoptive transfer studies showed that donor eosinophils from wild-type (WT), *Il4^−/−^* or *Il13^−/−^* mice did not differ in reversing the atherosclerotic findings in *Apoe^−/−^ΔdblGATA* recipient mice. Reduced atherosclerosis in *Apoe^−/−^ΔdblGATA* recipient mice was reversed by all these donor eosinophils (*Figure 1A–H*). Immunostaining showed that eosinophil deficiency or reconstitution of eosinophils from the same sets of mice did not affect aortic root and arch lesion macrophage or CD4^+^ T-cell contents, MHC class-II levels, or CD31^+^ microvessel numbers (see Supplementary data online, *Figure S3A–F*). Immunofluorescence double-staining with Ki67 and α-actin antibodies indicated that eosinophil deficiency did not affect lesion SMC proliferation (see Supplementary data online, *Figure S3G*). Nor did eosinophil deficiency or eosinophil reconstitution affect plasma total cholesterol, triglyceride, LDL, or HDL levels (see Supplementary data online, *Figure S4A*). Elevated aortic root and arch SMC content in *Apoe^−/−^ΔdblGATA* mice (*Figure 1B* and F) but no significant difference in lesion SMC proliferation (see Supplementary data online, *Figure S3G*) suggest a role for eosinophils in SMC death. Consistent with this hypothesis, we detected significant reduction of terminal deoxynucleotidyl transferase dUTP nick end labeling (TUNEL)-positive cells in aortic root and arch lesions from *Apoe^−/−^ΔdblGATA* mice (*Figure 2A* and *B*). Immunofluorescence double-staining with the cleaved caspase-3 and α-actin antibodies showed that eosinophil deficiency reduced apoptosis of lesional SMC. Adoptive transfer of eosinophils from WT, *Il4^−/−^* or *Il13^−/−^* mice reversed the lesion total cell apoptosis or lesion SMC apoptosis as well (*Figure 2A–D*). Irrelevant isotype-matched IgGs served as controls (*Figure 2C* and *D*). The assessment of the caspase-3 ^+^ α-actin^+^ apoptotic SMC contents in the intima from the aortic root or arch using the same immunofluorescence double-staining approach yielded similar results (see Supplementary data online, *Figure S4B* and *C*). This study did not detect significant differences in caspase-3 ^+^ CD68^+^ apoptotic macrophage content in either the media or intima from aortic root or arch (see Supplementary data online, *Figure S4D–E*). Unexpectedly, we detected vascular calcification in most of the aortic root and arch lesions from *Apoe^−/−^* mice, but not in lesions from the *Apoe^−/−^ΔdblGATA* mice. To quantify these differences in vascular calcification, we stained the lesion calcium deposition with Alizarin red^27,31^ and measured the vascular calcification marker ALP activity.^32^ Eosinophil deficiency blunted calcium deposition (*Figure 2E* and *F*) and ALP activity (*Figure 2G* and *H*) in lesions from aortic root and arch. Such reductions were all recovered by adoptive transfer of eosinophils from WT, *Il4^−/−^* or *Il13^−/−^* mice (*Figure 2E–H*), suggesting that eosinophils promote atherogenesis and aortic lesion calcification by mechanisms not involving IL4 or IL13. To confirm that donor eosinophils targeted and survived in the aorta, we transferred donor eosinophils from CD45.1 transgenic mice to *Apoe^−/−^ΔdblGATA* mice and then rendered them atherosclerotic and then performed Immunofluorescence double-staining to detect donor eosinophils in the lesions. Siglec-F-positive donor eosinophils in recipient mouse lesions expressed mEar1 (see Supplementary data online, *Figure S5A*) in addition to CD45.1, the marker of donor cells (Figure S5B).

### Eosinophils use cationic protein mEar1 to promote atherogenesis

**Figure 3:**
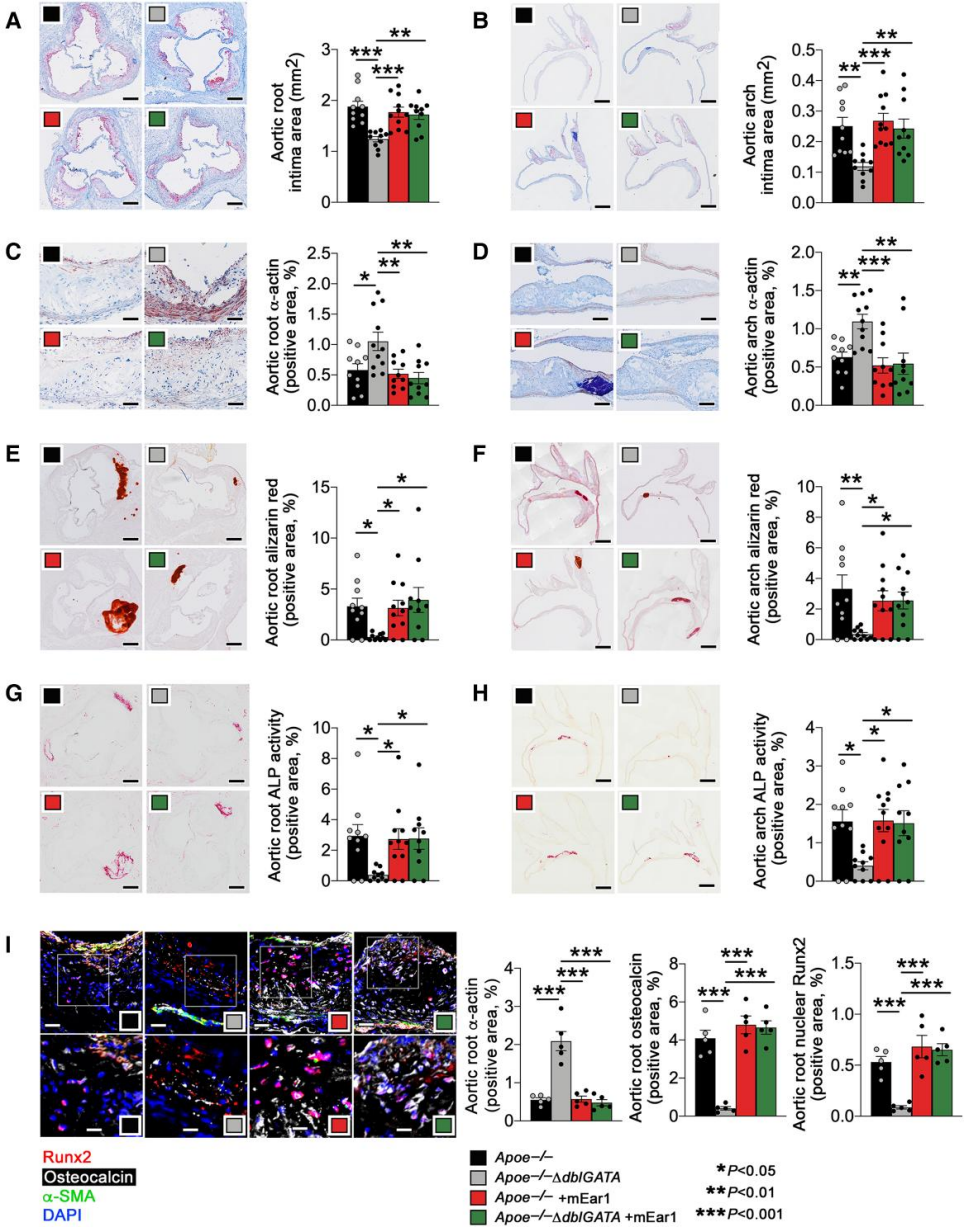
Mouse eosinophil-associated-ribonuclease-1 exacerbates atherogenesis and arterial calcification in *apoe^−/−^* mice. *Apoe^−/−^* and *Apoe^−/−^ ΔdblGATA* mice were fed with an atherosclerotic diet and administrated with recombinant mouse eosinophil-associated-ribonuclease-1 (5 μg/mouse/time) twice per week for 12 weeks. (*A*) Aortic root and (*B*) arch intima area. Scale: 500 μm. (*C*) Root and (*D*) arch α-actin^+^ smooth muscle cell contents. Scale: 200 μm. (*E*) Root and (*F*) arch Alizarin red-positive calcium deposition. Scale: 500 μm. (*G*) Root and (*H*) arch alkaline phosphatase activity-positive area. Scale: 500 μm. (*I*) Aortic root α-actin, osteocalcin, and nuclear Runt-related transcription factor-2 contents by immunofluorescence staining. Scale: 50 μm (top) and 25 μm (bottom). The results are expressed as mean ± standard error of mean of 10–11 (*A–H*) and 5 mice (*I*) per group.

In addition to cytokines (e.g. IL4 and IL13), eosinophils also release cationic proteins such as ECP and EDN^3,8^ that could account for reduced atherosclerosis and lesion calcification in *Apoe^−/−^ΔdblGATA* mice. To test this possibility, we administered recombinant mouse mEar1 to *Apoe^−/−^* and *Apoe^−/−^ΔdblGATA* mice by subcutaneous injection, followed by consumption of an atherogenic diet. This treatment reduced intimal area, increased SMC content, and lowered Alizarin red-positive area and ALP activity in atherosclerotic lesions in both aortic roots and arches in the *Apoe^−/−^ΔdblGATA* mice to the levels observed in *Apoe^−/−^* mice. These parameters did not change in *Apoe^−/−^* mice with or without mEar1 treatment (*Figure 3A–H*), suggesting the requirement of higher doses of mEar1. ELISA results showed the recovery of blunted plasma mEar1 in *Apoe^−/−^ΔdblGATA* mice to the levels in *Apoe^−/−^* mice in either *Apoe^−/−^* or *Apoe^−/−^ΔdblGATA* mice after mEar1 treatment (see Supplementary data online, *Figure S6A*). As in the *Apoe^−/−^ΔdblGATA* mice, mEar1 did not affect plasma lipids measured (see Supplementary data online, *Figure S6B*), the adipokine leptin (see Supplementary data online, *Figure S6C*), or pro-inflammatory cytokines (see Supplementary data online, *Figure S6D*) in *Apoe^−/−^* or *Apoe^−/−^ΔdblGATA* mice. Like in the *Apoe^−/−^ΔdblGATA* mice that received donor eosinophils from WT, *Il4^−/−^*, or *Il13^−/−^* mice (*Figure 2C* and *D*; see Supplementary data online, *Figure S4B–E*), mEar1 treatment also increased aortic root and arch intima caspase-3 ^+^ α-actin^+^ apoptotic SMC content in *Apoe^−/−^ΔdblGATA* mice (see Supplementary data online, *Figure S7A* and *B*). Eosinophil deficiency or mEar1 treatment also did not affect aortic root or aortic arch lesion caspase-3 ^+^ CD68^+^ macrophage content (see Supplementary data online, *Figure S7C and D*).

The transcription factor Runx2 is a master regulator for vascular SMC osteogenic transition and vascular calcification.^27,33,34^ The Runx2-regulated bone marker osteocalcin is the most abundant non-collagenous protein in bone^35^ and associates with vascular calcification.^36^ Eosinophil deficiency increased aortic root lesion α-actin SMC positive area but blunted lesion osteocalcin-positive area and lesion nuclear Runx2-positive area. Subcutaneous mEar1 treatment reversed these changes in *Apoe^−/−^ΔdblGATA* mice, but not in *Apoe^−/−^* mice (*Figure 3I*). Reduced lesion collagen deposition, aortic wall elastica fragmentation, total TUNEL-positive cell, and cleaved caspase-3 ^+^ α-actin^+^ SMC in both aortic roots and arches from *Apoe^−/−^ΔdblGATA* mice were also reversed in mice that received mEar1. The mEar1 treatment did not affect these variables in *Apoe^−/−^* mice (see Supplementary data online, *Figure S8A–H*). Nor did mEar1 affect aortic root or arch lesion macrophage and CD4^+^ T-cell content, MHC class-II-positive area, CD31^+^ microvessel numbers, and Ki67 ^+^ α-actin^+^ SMC proliferation in *Apoe^−/−^* or *Apoe^−/−^ΔdblGATA* mice (see Supplementary data online, *Figure S9A–G*). These observations suggest that eosinophils contributed to atherogenesis and vascular calcification in *Apoe^−/−^* mice via the cationic protein mEar1.

Immunofluorescence triple-staining demonstrated blunted calcified apoptotic SMC (caspase-3 ^+^ osteopontin ^+^ α-actin^+^), apoptotic SMC (caspase-3 ^+^ α-actin^+^), calcified apoptotic cells (caspase-3 ^+^ osteopontin^+^), and cellular calcification (osteopontin^+^) in aortic root atherosclerotic lesions in *Apoe^−/−^ΔdblGATA* mice. All these blunted phenotypes reverted when mice received subcutaneous mEar1 treatment (see Supplementary data online, *Figure S10*). Therefore, osteogenic differentiated SMC may be prone to undergoing apoptosis and *vice versa*. To test this possibility, we cultured mouse aortic SMC under normal and osteogenic media^37^ and induced cell apoptosis with or without pyrrolidine dithiocarbamate (PDTC). The results showed that osteogenic medium enhanced PDTC-induced apoptosis (increased cleaved caspase-3 and reduced Bcl-2), and PDTC also increased SMC calcification (increased expression of ALP and osteopontin, another sensitive marker of coronary artery calcification^38^) (see Supplementary data online, *Figure S11*).

### Eosinophil mEar1 promotes vascular SMC osteogenic differentiation

**Figure 4:**
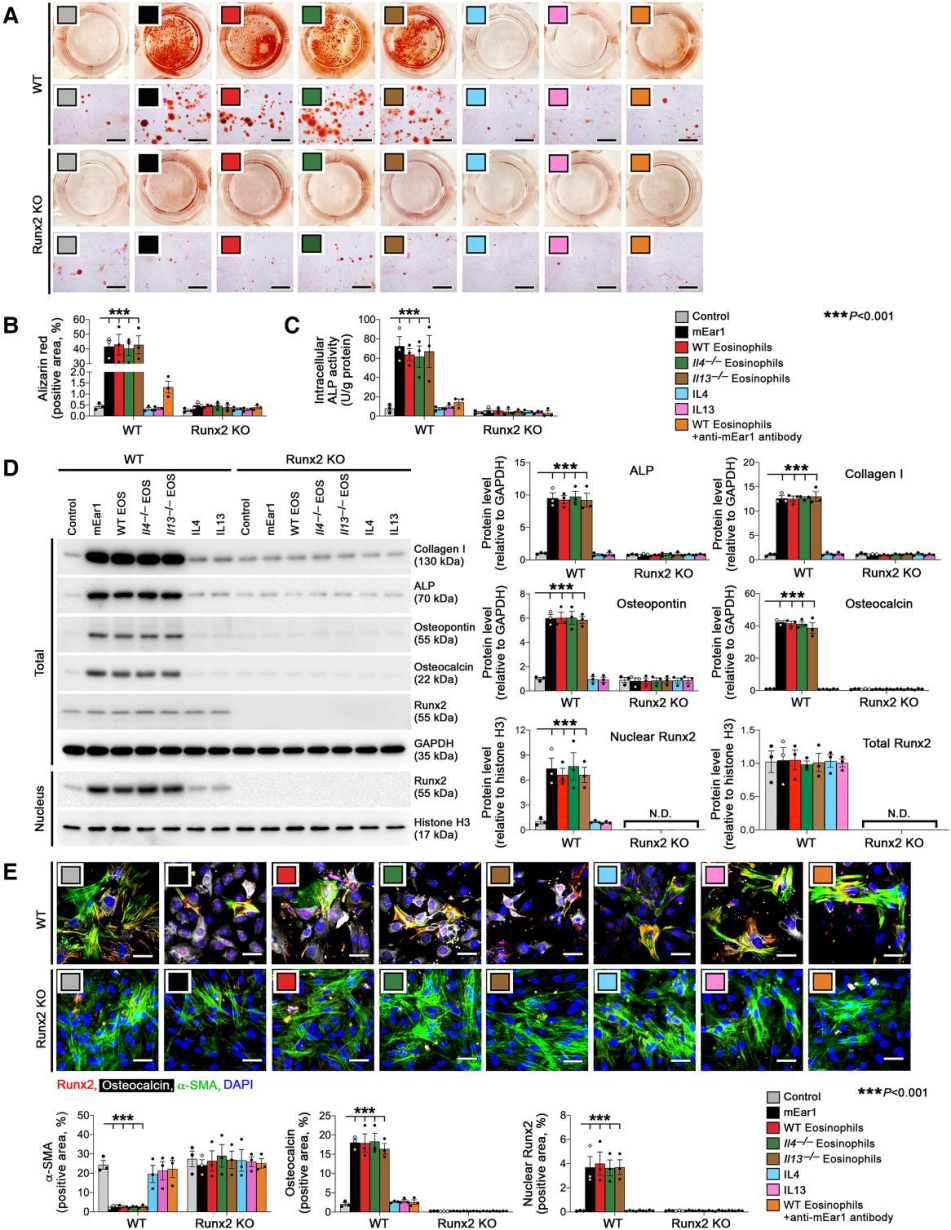
Mouse eosinophil-associated-ribonuclease-1 aggravates vascular smooth muscle cell calcification via Runt-related transcription factor-2. Wild-type and Runt-related transcription factor-2 KO mouse aortic smooth muscle cell were exposed to osteogenic media with or without mouse eosinophil-associated-ribonuclease-1, interleukin-4, or interleukin-13 proteins, eosinophil lysates from wild-type, *Il4^−/−^* or *Il13^−/−^* mice, and wild-type eosinophil lysate plus anti-mouse eosinophil-associated-ribonuclease-1 antibody. (*A*) Representative images of stained dishes (top), photomicrographs (bottom, Scale: 200 μm). Quantification of (*B*) Alizarin red staining for mineralized calcium and (*C*) intracellular alkaline phosphatase activity. (*D*) Immunoblots of collagen I, alkaline phosphatase, osteocalcin, osteopontin, total and nuclear Runt-related transcription factor-2. (*E*) Immunofluorescence staining of α-actin, osteocalcin, and Runt-related transcription factor-2. Scale: 50 μm. The results are expressed as mean ± standard error of mean of three independent experiments.

The results from atherogenic diet-fed *Apoe^−/−^* or *Apoe^−/−^ΔdblGATA* mice suggest that eosinophil mEar1, but not IL4 or IL13, promotes atherogenesis and vascular calcification. We tested a direct effect of eosinophils and mEar1 in SMC osteogenic differentiation using mouse aortic SMC from WT (Runx2*^f/f^*) mice. Recombinant mEar1 and eosinophils from WT, *Il4^−/−^*, or *Il13^−/−^* mice all promoted SMC osteogenic differentiation when cells were cultured in osteogenic media, as determined by Alizarin red staining (*Figure 4A* and *B*) and ALP activity (*Figure 4C*), suggesting that only mEar1 but not IL4 or IL13 directly affected SMC osteogenic differentiation. In support of this conclusion, recombinant IL4 or IL13 did not affect SMC osteogenic differentiation and mEar1 antibody blunted the osteogenic activity of eosinophils from WT mice (*Figure 4A–C*). Runx2 is required for vascular SMC osteogenic differentiation *in vitro* and *in vivo*.^33,34^ Using aortic SMC from the SMC-specific Runx2-depleted (Runx2*^f/f^*; SM22α-Cre) (Runx2 KO) mice,^27^ we demonstrated that treatments with mEar1, eosinophils from WT, *Il4^−/−^* or *Il13^−/−^* mice, eosinophils from WT mice together with mEar1 antibody, or recombinant IL4 and IL13, did not affect SMC osteogenic differentiation (*Figure 4A–C*). Immunoblot analyses yielded the same results. mEar1 and eosinophils from WT, *Il4^−/−^*, or *Il13^−/−^* mice, but not IL4 or IL13 enhanced the expression of collagen I, ALP, osteocalcin, and osteopontin in SMC from Runx2*^f/f^* mice, but not those from Runx2 KO mice (*Figure 4D*). mEar1 and eosinophils from WT, *Il4^−/−^* or *Il13^−/−^* mice, but not IL4 or IL13 also increased Runx2 nuclear translocation, although none of the treatments affected the expression of total cellular Runx2 (*Figure 4D*). Immunofluorescence triple-staining showed that mEar1 and eosinophils from WT, *Il4^−/−^*, or *Il13^−/−^* mice, but not IL4, IL13, or WT eosinophils treated with mEar1 antibody blunted the smooth muscle α-actin (α-SMA) expression, but increased the expression of osteocalcin and nuclear Runx2 in aortic SMC from control mice, indicating SMC osteogenic differentiation. None of these treatments affected the expression of α-SMA or osteocalcin in SMC from Runx2 KO mice (*Figure 4E*). At the mRNA level, mEar1 and eosinophils from WT, *Il4^−/−^* or *Il13^−/−^* mice, but not IL4, IL13, or WT eosinophils treated with mEar1 antibody increased the expression of ALP, collagen I, osteocalcin, and osteopontin in SMC from Runx2*^f/f^* mice but not in those from Runx2 KO mice (see Supplementary data online, *Figure S12A–D*). None of these treatments affected Runx2 expression (see Supplementary data online, *Figure S12E*). Together, these observations suggest that mEar1-mediated SMC osteogenic differentiation is mediated by Runx2 activation but not its total concentration.

### mEar1 binds to the BMP receptors on mouse aortic SMC

**Figure 5:**
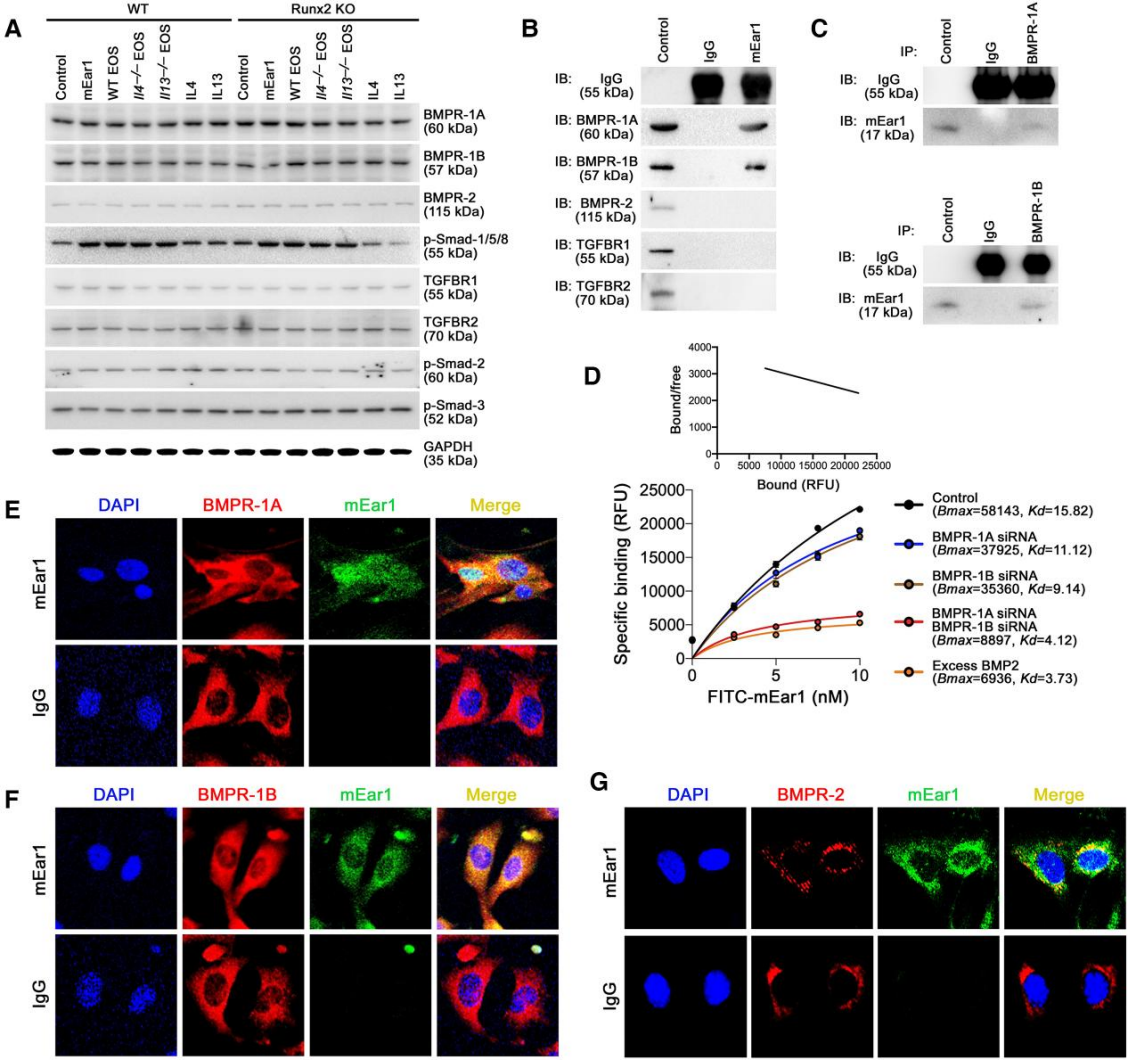
Mouse eosinophil-associated-ribonuclease-1 uses bone morphogenetic protein receptors to activate the smad-1/5/8-Runt-related transcription factor-2 signalling pathway. (*A*) Wild-type and Runt-related transcription factor-2 KO mouse smooth muscle cell were exposed to osteogenic media with or without mouse eosinophil-associated-ribonuclease-1, interleukin-4, interleukin-13, eosinophil lysates from wild-type, *Il4^−/−^* or *Il13^−/−^* mice. Immunoblots of different bone morphogenetic protein receptors, TGFβRs, and phosphorylated Smad-2, Smad-3, and Smad-1/5/8. (*B–G*) Wild-type smooth muscle cell were exposed to osteogenic media for 14 days and stimulated with mouse eosinophil-associated-ribonuclease-1 for 30 min before collection. Immunoprecipitation with anti-mouse eosinophil-associated-ribonuclease-1 (*B*), anti-bone morphogenetic protein receptor-1A (*C*, top) and anti-bone morphogenetic protein receptor-1B (*C*, bottom) antibodies, followed by immunoblotting detection of different bone morphogenetic protein receptors, TGFβRs, or mouse eosinophil-associated-ribonuclease-1. (*D*) FITC-mouse eosinophil-associated-ribonuclease-1 (0∼10.0 nM) binding affinity and Scatchard plot on smooth muscle cell treated with or without bone morphogenetic protein receptor siRNA or excessive BMP2 (1000 ng/mL). Immunofluorescence double-staining of mouse eosinophil-associated-ribonuclease-1 with bone morphogenetic protein receptor-1A (*E*), bone morphogenetic protein receptor-1B (*F*) or bone morphogenetic protein receptor-2 (*G*).

TGF-β-TGF-β receptors (TGFBR1/2)-Smad-2/3 pathway and the bone morphogenetic proteins (BMP2/4)-BMPR-1A/1B/2-Smad-1/5/8 pathway promote Runx2 activation during bone development.^39^ Immunoblot analyses demonstrated that mEar1 and eosinophils from WT, *Il4^−/−^*, or *Il13^−/−^* mice, or IL4 and IL13 did not affect the expression of BMPR-1A, BMPR-1B, BMPR-2, TGFBR1, and TGFBR2, nor the activation (phosphorylation) of Smad-2 and Smad-3 in aortic SMC from WT or Runx2 KO mice. Only mEar1 and eosinophils from control, *Il4^−/−^* or *Il13^−/−^* mice, but not IL4, IL13 activated p-Smad-1/5/8 in aortic SMC from WT and Runx2 KO mice (*Figure 5A*; see Supplementary data online, *Figure S13A–C*). At the mRNA level, none of these treatments affected the expression of BMPR-1A/1B/2 or TGFBR1/2 (see Supplementary data online, *Figure S13D–E*). Therefore, mEar1 may use the BMPR-1A/1B/2 rather than TGFBR1/2 as its receptors. To test this hypothesis, we added mEar1 to cultured mouse aortic SMC followed by immunoprecipitation with mEar1 antibody or its isotype IgG. Immunoprecipitates were examined by immunoblotting with different antibodies. The results showed that mEar1 formed immune complexes with BMPR-1A and BMPR-1B. mEar1 did not interact with BMPR-2 or the TGF-β receptors (TGFBR1/2) (*Figure 5B*). The interaction between mEar1 and BMPR-1A or BMPR-1B was confirmed by immunoprecipitation with BMPR-1A and BMPR-1B antibodies, followed by immunoblotting with mEar1 antibody. The results demonstrated complex formation of mEar1 with BMPR-1A and with BMPR-1B (*Figure 5C*). Cell surface binding assay using FITC-labelled mEar1 and Scatchard plot analysis showed that mEar1 bound to SMC (*Bmax* = 58143, *Kd* = 15.82 nM). Knockdown of BMPR-1A or BMPR-1B with their siRNAs reduced such binding (*Bmax* = 37925, *Kd* = 11.12 nM, or *Bmax* = 35360, *Kd* = 9.14 nM). Knockdown of both BMPR-1A and BMPR-1B or use of excess authentic BMPR ligand BMP2 fully blocked the FITC-mEar1 binding (*Figure 5D*). These observations suggest that mEar1 acted like BMP2 and used BMPR-1A and BMPR-1B as its receptors. Immunofluorescence double-staining with mEar1 and BMPR-1A or BMPR-1B antibodies revealed mEar1 colocalization with BMPR-1A (*Figure 5E*) and BMPR-1B (*Figure 5F*). Interestingly, under the same conditions, we also detected mEar1 localization with BMPR-2, although on many fewer cells (*Figure 5G*). Therefore, the absence of mEar1 interaction with BMPR-2 in our immunoprecipitation experiment (*Figure 5B*) was likely due to the low BMPR-2 expression in mouse aortic SMC (*Figure 5A*). Together, the results from immunoprecipitation, ligand binding, Scatchard plot analysis, and immunofluorescence staining indicated that mEar1 used BMPR-1A and BMPR-1B as its receptors on mouse aortic SMC. BMP2 and BMP4 are both authentic ligands for BMPR-1A and BMPR-1B.^40^ Eosinophil deficiency in *Apoe^−/−^ΔdblGATA* mice or mEar1 treatment in *Apoe^−/−^ΔdblGATA* mice may affect atherogenesis and vascular calcification indirectly by altering the expression of BMP2 or BMP4. Yet, ELISA did not detect any changes in plasma BMP2 or BMP4 levels between *Apoe^−/−^* and *Apoe^−/−^ΔdblGATA* mice or those received mEar1 (see Supplementary data online, *Figure S14*).

To confirm that mEar1 uses BMPR-1A, BMPR-1B, and possibly BMPR-2 as its receptors, we transfected all three receptors in 293T cells that have only basal levels of expression. Immunoblot analyses confirmed the transfection efficiency. Empty vectors served as negative controls (see Supplementary data online, *Figure S15A*). Cell surface binding assay and Scatchard plot analysis showed that FITC-mEar1 bound with high affinity to 293T cells that expressed BMPR-1A (*Bmax* = 54808, *Kd* = 10.58 nM) and BMPR-1B (*Bmax* = 59595, *Kd* = 9.45 nM) (see Supplementary data online, *Figure S15B*). FITC-mEar1 also bound to 293T cells that was transfected with BMPR-2, but at much lower affinity (*Bmax* = 18050, *Kd* = 6.09 nM) (see Supplementary data online, *Figure S15C*). Much higher binding affinity was detected in 293T cells transfected with both BMPR-1A and BMPR-1B (*Bmax* = 91814, *Kd* = 15.49 nM) (see Supplementary data online, *Figure S15D*). Combined expression of BMPR-1A with BMPR-2 or BMPR-1B with BMPR-2 also increased FITC-mEar1 binding affinity. Empty vector-transfected 293T cells (Control) showed no mEar1 binding (see Supplementary data online, *Figure S15D*). Excessive unlabelled mEar1 or BMP2 competed the binding of FITC-mEar1 on BMPR-1A- and BMPR-1B- transfected 293T cells (see Supplementary data online, *Figure S15B*) and also on BMPR-2-transfected 293T cells (see Supplementary data online, *Figure S15C*).

### Deficiency of BMPR-1a or 1B blocks mEar1 activity on SMC osteogenic differentiation

**Figure 6:**
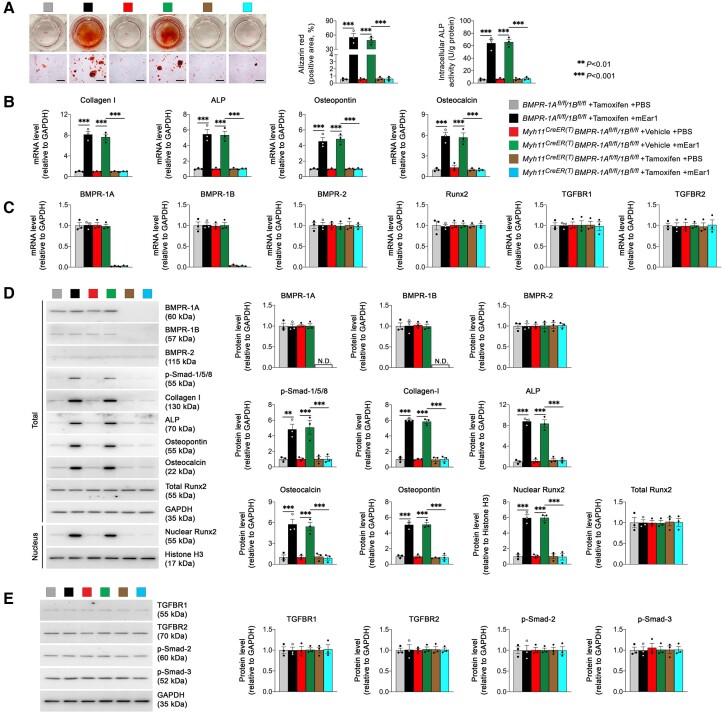
Tamoxifen-induced depletion of bone morphogenetic protein receptor-1A and bone morphogenetic protein receptor-1B in aortic smooth muscle cell blocks mouse eosinophil-associated-ribonuclease-1-induced calcification. Aortic smooth muscle cell were isolated from *BMPR-1A^fl/fl^/1B^fl/fl^* and *Myh11^CreER(T)^BMPR-1A^fl/fl^/1B^fl/fl^* mice and treated with tamoxifen (0.5 µM) or vehicle (DMSO) for 24 h. Cells were then exposed to osteogenic media with or without mouse eosinophil-associated-ribonuclease-1 for 14 days. *(A*) Representative images of Alizarin red-stained dishes (top) and photomicrographs (bottom, scale: 200 um), and quantifications of Alizarin red staining for mineralized calcium and intracellular alkaline phosphatase activity. (*B* and *C*) RT-PCR detected the expression osteogenetic genes (*B*), bone morphogenetic protein receptor, Runt-related transcription factor-2, and TGF-β receptors (*C*). (*D* and *E*) Immunoblot analysis of bone morphogenetic protein receptor signalling molecules and osteogenetic proteins (*D*) and TGF-β receptors and signalling molecules (*E*). lRepresentative images are presented to the left (*A, D, E*). Data are mean ± standard error of mean from three independent experiments.

To test a direct role for BMPR-1A or BMPR-1B on mEar1-mediated SMC osteogenic differentiation, we bred the *BMPR-1A^fl/fl^* and *BMPR-1B^fl/fl^* mice with *Myh11^CreER(T)^* mice and generated *Myh11^CreER(T)^BMPR-1A^fl/fl^, Myh11^CreER(T)^BMPR-1B^fl/fl^*, *BMPR-1A^fl/fl^/1B^fl/fl^*, and *Myh11^CreER(T)^BMPR-1A^fl/fl^/1B^fl/fl^* mice. Aortic SMC from these mice were treated with tamoxifen to induce the depletion of BMPR-1A, BMPR-1B, or both. As expected, mEar1 induced SMC osteogenic differentiation with increased Alizarin red staining, intracellular ALP activity, and expression (mRNA) of collagen I, ALP, osteopontin, and osteocalcin in cells from *BMPR-1A^fl/fl^* mice or *Myh11^CreER(T)^BMPR-1A^fl/fl^* mice after vehicle treatment. Such activities of mEar1 were muted in cells from *Myh11^CreER(T)^BMPR-1A^fl/fl^* mice after tamoxifen-induced BMPR-1A depletion (see Supplementary data online, *Figure S16A* and *B*). BMPR-1A depletion or mEar1 treatment did not affect the expression (mRNA) of BMPR-1B/2, Runx2, and TGFBR1/2 (see Supplementary data online, *Figure S16C*). Immunoblot analyses yielded the same conclusions. BMPR-1A depletion or mEar1 treatment did not affect the expression of BMPR-1B/2, total Runx2, TGFBR1/2, and p-Smad-2/3 signalling. mEar1 lost its activities in activating p-Smad-1/5/8 and in inducing the expression of collagen I, ALP, osteopontin, and osteocalcin, and nuclear Runx2 (see Supplementary data online, *Figure S16D–E*). Aortic SMC from *BMPR-1B^fl/fl^* and *Myh11^CreER(T)^BMPR-1B^fl/fl^* mice (see Supplementary data online, *Figure S17A–E*) or from *BMPR-1A^fl/fl^/1B^fl/fl^* and *Myh11^CreER(T)^BMPR-1A^fl/fl^/1B^fl/fl^* mice (*Figure 6A–E*) yielded the same conclusions as those from *BMPR-1A^fl/fl^* mice or *Myh11^CreER(T)^BMPR-1A^fl/fl^* mice. Therefore, SMC osteogenic differentiation requires BMPR-1A and BMPR-1B to mediate mEar1 activity.

### ECP and EDN bind to the BMP receptors on human aortic SMC

**Figure 7:**
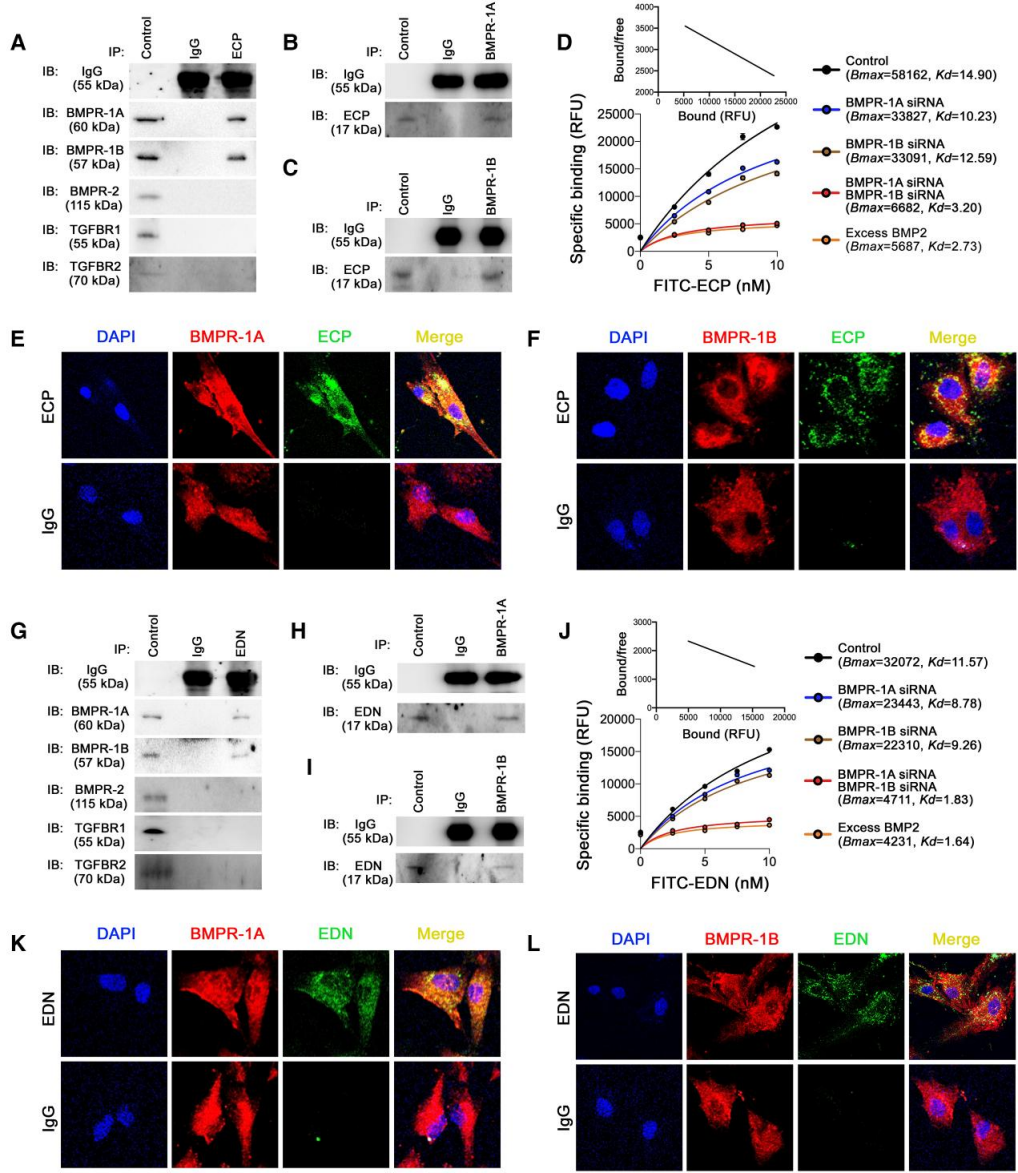
Binding of eosinophil cationic protein and eosinophil-derived neurotoxin on human smooth muscle cell bone morphogenetic protein receptors. Human smooth muscle cell were exposed to osteogenic media for 14 days and stimulated with recombinant eosinophil cationic protein or eosinophil-derived neurotoxin for 30 min before collection. Immunoprecipitation with anti-eosinophil cationic protein (*A*), anti-bone morphogenetic protein receptor-1A (*B*) and anti-bone morphogenetic protein receptor-1B (*C*) antibodies, followed by immunoblotting detection of different bone morphogenetic protein receptors, TGFβRs, and eosinophil cationic protein. (*D*) FITC-eosinophil cationic protein (0∼10.0 nM) binding affinity and Scatchard plot on smooth muscle cell treated with or without bone morphogenetic protein receptor siRNA or excessive BMP2 (1000 ng/mL). Immunofluorescence double-staining of eosinophil cationic protein and bone morphogenetic protein receptor-1A (*E*) or bone morphogenetic protein receptor-1B (*F*). Immunoprecipitation with anti-eosinophil-derived neurotoxin (*G*), anti-bone morphogenetic protein receptor-1A (*H*), and anti-bone morphogenetic protein receptor-1B (*I*) antibodies, followed by immunoblotting detection of different bone morphogenetic protein receptors, TGFBRs, and eosinophil-derived neurotoxin. (*J*) FITC-eosinophil-derived neurotoxin (0∼10.0 nM) binding affinity and Scatchard plot on smooth muscle cell treated with or without with bone morphogenetic protein receptor siRNA or excessive BMP2 (1000 ng/mL). Immunofluorescence double-staining of eosinophil-derived neurotoxin and bone morphogenetic protein receptor-1A (*K*) or bone morphogenetic protein receptor-1B (*L*).

mEar1 is a murine homologue of human ECP.^41^ Human ECP and EDN share similar tertiary sequence structure and surface charges with mEar1.^17^ mEar1 binding on BMPR-1A/1B and less efficiently on BMPR-2 suggests that human ECP and EDN also use BMPR-1A, BMPR-1B, and possibly BMPR-2 as their receptors on vascular SMC. After human vascular SMCs were treated with ECP followed by immunoprecipitation with ECP antibody, we performed immunoblot analyses with various antibodies and found that human ECP formed complexes with BMPR-1A and BMPR-1B, but not BMPR-2 or TGFBR1/2 (*Figure 7A*). Reverse immunoprecipitation with BMPR-1A and BMPR-1B antibodies followed by immunoblotting with ECP antibody confirmed the formation of ECP-BMPR-1A and ECP-BMPR-1B immune complexes (*Figure 7B* and *C*). Ligand binding assay and Scatchard plot analysis showed that FITC-ECP bound to human aortic SMC (*Bmax* = 58162, *Kd* = 14.90). The binding affinity fell after siRNA knockdown of BMPR-1A or BMPR-1B and was abrogated by siRNA knockdown of both BMPR-1A and BMPR-1B or by excess BMP2 (*Figure 7D*). Immunofluorescence double-staining revealed colocalization of ECP with BMPR-1A and BMPR-1B on human aortic SMC (*Figure 7E* and *F*).

EDN acted directionally similar to ECP, but bound to BMPR-1A and BMPR-1B at much lower affinity. Immunoprecipitation with EDN antibody followed by immunoblotting demonstrated complex formation of EDN with BMPR-1A and BMPR-1B, but not with BMPR-2 or TGFBR1/2 (*Figure 7G*). Reverse immunoprecipitation with BMPR-1A and BMPR-1B antibodies followed by immunoblotting with EDN antibody confirmed complex formation of EDN with BMPR-1A and BMPR-1B in human aortic SMC (*Figure 7H* and *I*). Cell binding assay and Scatchard plot analysis showed that EDN also bound to BMPR-1A and BMPR-1B. This binding fell after BMPR-1A or BMPR-1B knockdown with their cognate siRNAs or was abrogated by siRNA knockdown of both BMPR-1A and BMPR-1B or with excess BMP2 (*Figure 7J*). Yet, EDN bound to BMPR-1A and BMPR-1B at much lower affinity (*Bmax* = 32072, *Kd* = 11.57) than ECP did (*Bmax* = 58162, *Kd* = 14.90) (*Figure 7D* and *J*). Immunofluorescence double-staining also revealed co-localization of EDN with BMPR-1A and BMPR-1B on human aortic SMC (*Figure 7K–L*).

### ECP and EDN use BMP receptors to promote human vascular SMC osteogenic transition

**Figure 8:**
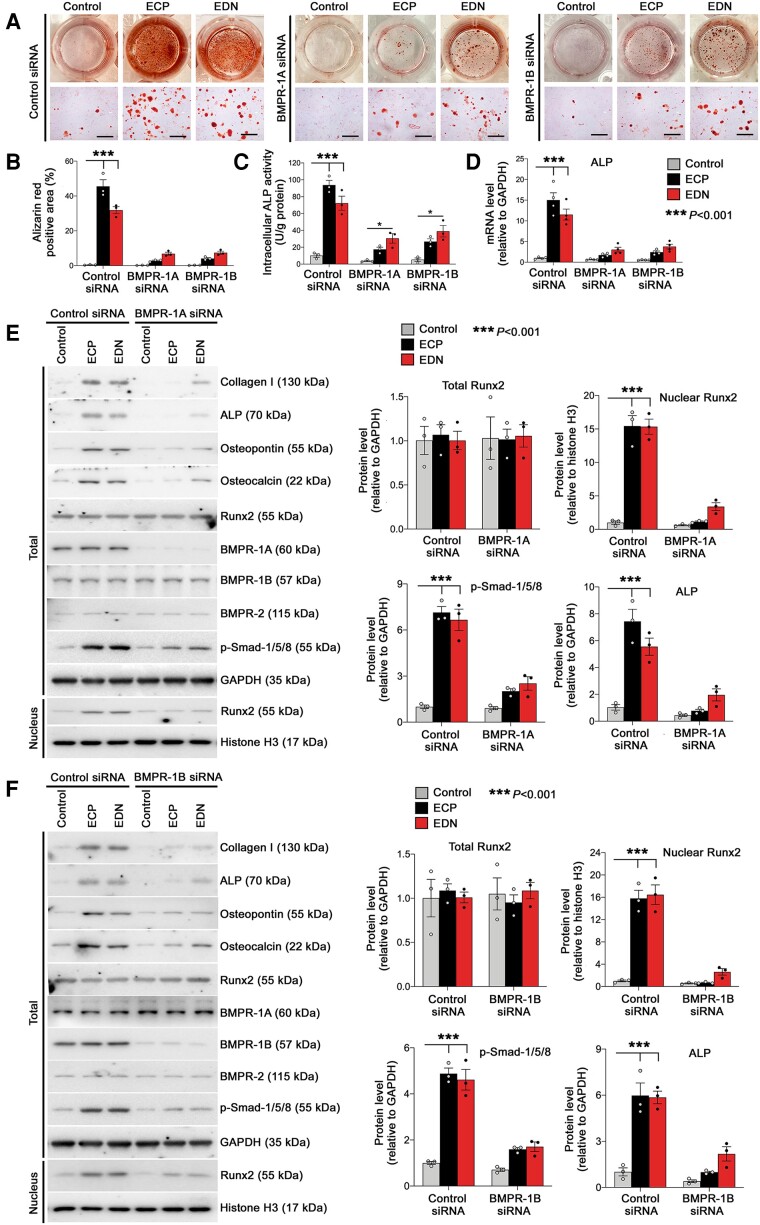
Human bone morphogenetic protein receptor silencing ameliorates eosinophil cationic protein- and eosinophil-derived neurotoxin-induced human smooth muscle cell calcification. Human vascular smooth muscle cell were transfected with bone morphogenetic protein receptor-1A, bone morphogenetic protein receptor-1B, or control siRNA and then exposed to osteogenic media with or without recombinant eosinophil cationic protein or eosinophil-derived neurotoxin for 14 days. (*A*) Representative images of stained dishes (top) and photomicrographs (bottom. Scale: 200 μm.) along with quantification (*B*) of Alizarin red staining for mineralized calcium, (*C*) intracellular alkaline phosphatase activity, and (*D*) alkaline phosphatase gene expression. Immunoblot analysis of different bone morphogenetic protein receptor-Smad-Runt-related transcription factor-2 signalling proteins in human vascular smooth muscle cell treated with or without eosinophil cationic protein or eosinophil-derived neurotoxin after cells were treated with bone morphogenetic protein receptor-1A (*E*), bone morphogenetic protein receptor-1B (*F*), and control siRNAs. The results are expressed as mean ± standard error of mean from three to four independent experiments.

Like mEar1, ECP and EDN also used BMPR-1A and BMPR-1B to promote human aortic SMC osteogenic differentiation. Both ECP and EDN enhanced the Alizarin red-positive area, ALP activity, and ALP mRNA levels in human aortic SMC. BMPR-1A siRNA or BMPR-1B siRNA blunted these activities of ECP and EDN (*Figure 8A–D*). Immunoblot analyses showed that ECP and EDN increased the expression of collagen I, ALP, osteopontin, and osteocalcin, activated p-Smad-1/5/8, and enhanced Runx2 nuclear translocation. Treated with BMPR-1A siRNA(*Figure 8E*; see Supplementary data online, *Figure S18A*) or with BMPR-1B siRNA (*Figure 8F*; see Supplementary data online, *Figure S19A*) muted all of these activities of ECP and EDN in human SMC. However, ECP or EDN did not affect the expression of Runx2, BMPR-1A/1B, BMPR-2, TGFBR1/2, and their downstream *P*-Smad-2/3 (*Figure 8E* and *F*; see Supplementary data online, *Figures S18B* and *C, S19B* and *C*). At the mRNA level, ECP and EDN also increased the expression of collagen I, osteocalcin, and osteopontin in human aortic SMC. These activities of ECP and EDN were muted after human SMC were treated with BMPR-1A siRNA or BMPR-1B siRNA (see Supplementary data online, *Figure S20A*). ECP, EDN, BMPR-1A siRNA, or BMPR-1B siRNA did not affect the expression of BMPR-1A/1B, BMPR-2, TGFBR1/2, or Runx2 (see Supplementary data online, *Figure S20B–E*). Together, these observations suggest that ECP and EDN both use BMPR-1A/1B to promote human vascular SMC osteogenic differentiation.

### Blood eosinophil counts and ECP levels are associated with human aortic artery calcification

Blood eosinophil counts have been associated with coronary artery calcification (CAC) in patients with coronary heart disease (CHD).^42,43^ We took the advantage of our on-going DANCAVAS trial,^21^ a population-based randomized clinically controlled screening trial primarily designed to evaluate the health benefits and cost-effectiveness of using computer tomography (CT) scans to measure CAC and to identify aortic/iliac aneurysms and to measure the ankle brachial blood pressure index as part of a multifocal screening and intervention programme for CVD in men aged 65–74. From January 2015 to August 2018, 5864 men had their blood eosinophil counts measured (see Supplementary data online, *Table S1*). Additionally, a sub-population of 400 participants had measurement of plasma ECP concentrations. After few exclusions from missing information, 394 ECP measurements were included in the analyses (197 with non-existing CAC and 197 with high CAC). In addition to CAC, calcification scores of other arteries were also available, including aortic valve, abdominal suprarenal aorta, mitral valve, ascending aorta, aortic arch, descending aorta, renal artery, infrarenal aorta, and iliac artery. Because both our blood eosinophil counts and calcification scores were heavily left-skewed even after transformation, we performed non-parametric Spearman’s correlation test. The plasma ECP levels were equally left skewed but were close to normally distributed upon logarithmic transformation. Thus, the correlations between unadjusted ECP levels and calcification scores were evaluated using the Spearman’s correlation test, whilst the adjusted correlations between the logarithmic transformed ECP levels and calcification scores were evaluated using the multivariate linear regression analyses. The Spearman’s Rho between blood eosinophil counts and plasma ECP levels was 0.23 (*P* < 0.001) and the Pearson’s r between the log-transformed eosinophil counts and ECP levels was 0.21 (*P* < 0.001). Consistent with earlier studies, we showed that the blood eosinophil counts correlated significantly with CAC (*P* = 0.011). In addition, blood eosinophil counts also correlated with the calcification scores of aortic valve (*P* = 0.023), aortic arch (*P* = 0.001), infrarenal aorta (*P* = 0.014), and iliac artery (*P* = 0.005), but not with calcification scores from the abdominal suprarenal aorta, mitral valve, ascending and descending aortas, and renal artery (*Table 1*; see Supplementary data online, *Figure S21*). Plasma ECP levels also correlated significantly with CAC (*P* < 0.001) and with the calcification scores of abdominal suprarenal aorta (*P* < 0.001), mitral valve (*P* = 0.002), aortic arch (*P <* 0.001), descending aorta (*P* = 0.008), renal artery (*P* = 0.008), infrarenal aorta (*P <* 0.001), and iliac artery (*P* = 0.005), but not with calcification of the aortic valve and ascending aorta (*Table 1*; see Supplementary data online, *Figure S22*). After adjusting for the potential blood eosinophil association confounders (*P* < 0.100) in Supplementary data online, *Table S1*, including smoking, diabetes, COPD, use of anti-platelet, angiotensin-converting enzyme inhibitor, and glucocorticoids, as well as high blood pressure, and obesity, blood eosinophil counts remained correlated significantly with the calcification scores of coronary artery (*P* = 0.017), aortic arch (*P* = 0.010), infrarenal artery (*P* = 0.011), and iliac artery (*P* = 0.009) (see Supplementary data online, *Table S2*). After adjusting for the same potential confounders for the logarithmic transformed ECP levels, a significant linear correlation was observed between ECP levels and calcification scores from CAC (*P* = 0.001), descending aorta (*P <* 0.001), suprarenal aorta (*P* = 0.014), infrarenal aorta (*P <* 0.001), renal artery (*P <* 0.001), and iliac artery (*P* = 0.034), respectively. Calcification scores were also compared between the quartiles of blood eosinophil counts from each artery category using the Kruskal–Wallis test. The results showed that the eosinophil count quartiles correlated significantly with the calcification scores in the coronary artery (*P* = 0.004), ascending aorta (*P* = 0.038), aortic arch (*P* = 0.002), infrarenal artery (*P* = 0.017), and iliac artery (*P* = 0.008) (*Table 2*). This was not tested for plasma ECP levels, as the subpopulation undergoing ECP measurements was already stratified according to level of CAC levels (non-existing CAC vs. high levels of CAC). Together, these clinical data support an association of blood eosinophil counts and plasma ECP levels with the calcification scores of arteries from coronary arteries to the iliac arteries.

**Table 1:** Non-parametric univariate rho correlations between blood EOS counts or ECP levels and calcification scores from various aortic segments, arteries, and heart valves

	Coronary artery	Aortic valve	Abdominal superarenal aorta	Mitral valve	Ascending aorta	Aortic arch	Descending aorta	Renal artery	Infra-renal aorta	Iliac artery
**Blood EOS counts**										
Patient number	5541	5490	5864	5513	5520	5525	4441	4426	4440	4423
Spearman’s correlation coefficient	0.034^a^	0.031^a^	0.015	0.014	−0.001	0.043^b^	0.005	0.017	0.037^a^	0.042^a^
Lower 95% C.I.	0.007	0.003	−0.015	−0.013	−0.028	0.016	−0.026	−0.014	0.007	0.012
Upper 95% C.I.	0.061	0.058	0.045	0.042	0.026	0.070	0.035	0.047	0.067	0.072
*P* value	0.011	0.023	0.323	0.283	0.957	0.001	0.764	0.264	0.014	0.005
**Plasma ECP levels**										
Patient number	394	382	343	390	394	394	344	342	343	340
Spearman’s correlation coefficient	0.188^b^	0.041	0.185^b^	0.158^b^	0.087	0.178^b^	0.144^b^	0.142^b^	0.179^b^	0.154^b^
*P* value	<0.001	0.423	<0.001	0.002	0.084	<0.001	0.008	0.008	<0.001	0.005

Correlation is significant at the 0.05 level (two-tailed).

Correlation is significant at the 0.01 level (two-tailed).

**Table 2:** Comparison of EOS count quartiles and calcification scores in various aortic segments, arteries, and heart valves

Arteries	Quartiles	*n*	Mean	SD	25th	50th	75th	*P*-value
**Coronary artery calcium score**	Lowest	1189	449.5	850.7	8.6	104.0	473.3	0.004
	Second	1562	372.1	698.0	6.7	102.5	405.7	
	Third	1352	427.8	750.6	11.1	119.5	516.2	
	Upper	1438	505.8	870.1	13.0	141.0	573.2	
**Aortic valve calcium score**	Lowest	1177	98.2	320.1	0.0	5.6	69.6	0.160
	Second	1556	96.0	294.7	0.0	6.2	75.0	
	Third	1339	107.5	369.7	0.0	7.1	87.6	
	Upper	1418	126.0	361.0	0.0	6.3	91.5	
**Mitral valve calcium scores**	Lowest	1185	57. 8	401.8	0.0	0.0	1.5	0.081
	Second	1552	61.0	467.2	0.0	0.0	0.0	
	Third	1347	50.8	275.6	0.0	0.0	0.8	
	Upper	1429	80.6	501.6	0.0	0.0	1.6	
**Ascending aorta calcium scores**	Lowest	1183	92.0	426.9	0.0	0.0	8.0	0.038
	Second	1555	59.8	223.8	0.0	0.0	4.0	
	Third	1349	85.5	333.7	0.0	0.0	5.0	
	Upper	1433	106.4	413.3	0.0	0.0	10.5	
**Aortic arch calcium scores**	Lowest	1184	741.5	1621.1	25.0	215.5	728.8	0.002
	Second	1555	796.6	6029.0	20.0	196.0	724.0	
	Third	1351	742.5	1293.6	20.0	226.0	867.0	
	Upper	1435	867.3	1742.8	30.0	277.0	940.0	
**Descending aorta calcium scores**	Lowest	859	919.2	2816.4	8.0	57.0	503.0	0.180
	Second	1253	718.2	2110.9	7.0	48.0	464.0	
	Third	1126	637.8	1586.0	5.0	52.5	463.0	
	Upper	1203	740.3	1797.7	6.0	68.0	608.0	
**Renal artery calcium scores**	Lowest	855	46.7	189.5	0.0	0.0	7.0	0.504
	Second	1253	34.6	119.8	0.0	0.0	4.0	
	Third	1118	36.3	124.8	0.0	0.0	5.0	
	Upper	1200	46.8	158.0	0.0	0.0	9.8	
**Infrarenal artery calcium scores**	Lowest	858	3223.5	4446.0	321.0	1610.0	4514.8	0.017
	Second	1253	2870.3	3736.4	311.0	1488.0	4048.0	
	Third	1125	2996.3	3567.9	402.0	1601.0	4389.5	
	Upper	1204	3480.6	5396.0	427.0	1844.5	4980.3	
**Iliac artery calcium scores**	Lowest	854	2807.3	4946.2	196.0	1034.0	3112.5	0.008
	Second	1249	2347.1	3520.4	160.5	1032.0	3025.5	
	Third	1121	2533.0	3560.7	207.0	1111.0	3375.0	
	Upper	1199	2953.3	4493.6	281.0	1253.0	3876.0	

## Discussion

Earlier studies indicated a reparative role for eosinophils in the myocardium and abdominal aorta by releasing cationic protein mEar1 and cytokines IL4 and IL13 to protect cardiomyocytes, cardiac fibroblasts, SMC, endothelial cells (ECs), macrophages, and monocytes from pathologic activation after cardiac and aortic injury.^16–18,44^ Here, we report a pathogenic role for eosinophils in atherogenesis by releasing cationic proteins mEar1, or ECP and EDN in humans, to promote SMC apoptosis and osteogenic differentiation and vascular calcification. Although eosinophils are rich in cytokines or growth factors such as IL4, IL5, IL6, IL13, and TGF-β,^45^ our results using eosinophils from *Il4^−/−^* and *Il13^−/−^* mice and recombinant IL4 and IL13 did not support a role for these cytokines in SMC osteogenic differentiation, vascular calcification, and atherogenesis. We identified that eosinophil-derived mEar1 directly participated in vascular calcification and atherogenesis in the present study. Marx *et al.* demonstrated that eosinophil-derived major basic proteins participate in platelet activation and thrombus formation.^19^ Thus both studies support a contribution of eosinophils to atherothrombosis. This conclusion differs from the observations of Hofheinz *et al.*, who claimed a negligible role of eosinophils in atherosclerosis after transferring bone-marrow from *ΔdblGATA* mice to atherosclerosis-prone *Ldlr^−/−^* mice,^20^ suggesting that the use of bone-marrow transfer is not specific to eosinophils.

The presence and quantification of CAC define coronary atherosclerosis,^46^ and represent an anatomic measure of coronary plaque burden.^47^ CAC is a risk factor for cardiovascular events^48^ and positively associates with blood eosinophil counts.^42,43^ In a cross-sectional study of 1363 patients with CHD, patients with significant stenosis had higher blood eosinophil counts and positively correlated with the CAC level (Log[CAC + 1]).^42^ In a multi-center longitudinal study of 3094 patients from the coronary artery risk development in young adults, eosinophil counts again associated with CAC in 566 patients after 20 years of follow-up.^43^ These clinical studies support an atherogenic role for eosinophils and their cationic proteins ECP and EDN in promoting aortic wall SMC calcification.

Eosinophil cationic proteins were first reported 50 years ago,^41,49^ and human ECP has been used as a biomarker for many human diseases, including CVD, cancers, pulmonary diseases, infectious diseases, and even recent COVID-19.^13,50–54^ Human ECP is cytotoxic and inhibits T-cell responses to antigens and B lymphocytes, activates mast cells, and basophils and increases epithelial cell expression of insulin-like growth factor-1 and intracellular adhesion molecule-1^55^ that in turn stimulates eosinophil release of ECP and EDN.^56^ ECP also activates fibroblasts to produce proteoglycans.^57^ We recently reported that mEar1 protects mouse cardiomyocytes from apoptosis or hypertrophy, inhibits fibroblast fibrotic protein expression and *P*-Smad-2/3 signalling, blocks leukocyte adhesion on ECs, inactivates the NF-κB signalling pathway in macrophages, EC, and SMC, promotes M2 macrophage and Ly6^lo^ monocyte polarization, and triggers eosinophil IL4 secretion.^16–18^ However, it remains unknown how mEar1/ECP/EDN target these cells, and whether there is a receptor or receptors involved in mediating these mEar1/ECP/EDN activities. Here we discovered that mEar1/ECP/EDN use the BMPR-1A and BMPR-1B on mouse and human aortic SMC to activate the p-Smad-1/5/8-Runx2 signalling pathway and consequent osteogenic differentiation and vascular calcification. Not only SMC but also cardiomyocytes, cardiac fibroblasts, EC, epithelial cells, and immune cells may all use these BMP receptors, a hypothesis that is essential to explore in different diseases where eosinophils participate.

BMP2 and BMP4 are members of the TGF-β family that induce the formation of bone and cartilage.^58^ When TGF-β binds to TGFBR1/2 heterodimers and activates the Smad-2/3 signalling pathway for fibrotic protein expression,^59^ BMP-2/4 bind to BMPR-1A/1B, BMPR-2, or BMPR-1A/1B/2 tetramer and activate the Smad-1/5/8 signalling and downstream Runx2.^60,61^ BMP-2/4 preferentially use BMPR-1A/1B as their receptors, which form a homodimer or heterodimer.^62^ BMP2/4 bind to BMPR-2 with much lower affinity than BMPR-1A/1B, but the ligand binding affinity increases when BMPR-1A/1B and BMPR-2 form a tetrameric complex.^62–65^ Here, we showed that mEar1 acted the same as BMP2/4 with much higher affinity to BMPR-1A/1B than to BMPR-2. We were only able to detect such differences in 293 T cells that were transfected with BMPR-1A, BMPR-1B, or BMPR-2, but not in cultured mouse and human aortic SMC. In mouse aortic SMC, we did not detect immune complex formation of mEar1 with BMPR-2. This does not mean that mEar1 does not use BMPR-2 as its receptor. Indeed, immunofluorescence staining did show mEar1 colocalization with BMPR-2. Failure to detect mEar1 interaction with BMPR-2 might be due to the low expression of BMPR-2 in mouse aortic SMC. Human ECP and EDN yielded the same results. The expression of BMPR-2 was much lower than that of BMPR-1A/1B. Therefore, immunoprecipitation with ECP or EDN followed by immunoblot analyses only detected BMPR-1A and BMPR-1B, but not BMPR-2. These results suggest that eosinophil cationic proteins use BMPR-1A/1B as their receptors. They also use BMPR-2 in cells that express this receptor, a hypothesis that was not thoroughly tested in this study.

Together, this study reports a pathogenic role for eosinophils in atherogenesis by releasing cationic proteins to promote vascular calcification. Most importantly, this study discovers that eosinophil cationic proteins mEar1 in mice and ECP and EDN in humans use the BMP receptors BMPR-1A/1B on SMC to activate the Smad-1/5/8 signalling without the involvement of the TGF-β receptors and Smad-2/3 pathway. Eosinophil cationic proteins also use BMPR-2 as their receptor, although this receptor is negligible on mouse and human vascular SMC. Therefore, our study suggests that human ECP and EDN use BMPRs as their receptors to mediate eosinophil pathogenic or reparative activities, depending on the disease and cell types. Blockade of ECP and EDN binding on these BMPRs and silencing of the Smad-1/5/8 signalling pathway may have therapeutic potential to control eosinophil-associated human diseases, including CVD (*Structured Graphical Abstract*).

### Study limitations

Clinical studies based on blood eosinophil counts yielded different conclusions regarding the possible eosinophil involvement in human CVD. We cannot explain why some studies showed low blood eosinophil counts in patients with MACEs, non-STEMI, or acute STEMI,^9,10^ but others showed high blood eosinophils in patients with established CVD risk factors, record of percutaneous or surgical coronary revascularization, and antihypertensive and antiplatelet therapy, and those with high CVD mortality rate.^11,12^ Findings that support a reparative role of eosinophils in post-MI cardiac injury, cardiac hypertrophy, and AAA formation,^16–18^ but a pathogenic function of eosinophils in atherogenesis, also remain unexplained. SMC calcification may not influence experimental MI, cardiac hypertrophy, or AAA. Atherosclerosis is a chronic disease. Diet-induced experimental atherosclerosis took at least three months while left anterior-descending coronary artery ligation-induced MI,^16^ pressure overload- and β-adrenergic receptor agonist isoproterenol-induced cardiac hypertrophy,^17^ and peri-vascular CaCl_2_ injury or angiotensin-II perfusion-induced AAA^18^ occurred in less than 4 weeks. Such differences in the time course of experimental disease development may contribute to the apparently contradictory roles of eosinophils under different conditions. Our recent unpublished clinical observations support this hypothesis. In patients with acute MI, low blood eosinophil counts at admission strongly predicted short-term all-cause and cardiac deaths after hospital discharge. In contrast, high blood eosinophil counts at 5∼7 days after acute MI onset associate with long-term all-cause mortality.

This study proposed a role for Runx2 in mEar1/ECP/EDN-mediated p-Smad-1/5/8 activation and osteogenic differentiation. Although not tested, Runx2 may not act as the only downstream mechanism of mEar1/ECP/EDN-mediated SMC calcification. Eosinophils or mEar1/ECP/EDN increased SMC expression of ALP, a known enzyme involved in SMC calcification.^66^ The expression or functions of transcription factor osterix and transcription repressor msh homeobox-2 are also downstream targets of p-Smad-1/5/8 activation,^67^ which eosinophils and mEar1/ECP/EDN may regulate.

The DANCAVAS trial is a large on-going cardiovascular screening trial of 45 000 men, although we selected only 5864 men with blood eosinophil counts available. This trial includes detailed calcification scores from different arteries and aortic/iliac aneurysm information, but these patients do not have plasma concentrations of the arterial calcification-associated molecules osteocalcin and osteopontin, nor do they have the eosinophil molecules ECP and EDN.^21^ Here we only measured plasma ECP levels from limited number of subjects (*n* = 394), which may underestimate the correlation between plasma ECP levels and aortic or arterial calcification scores. The reduced aortic wall calcification in eosinophil-deficient *Apoe^−/−^ΔdblGATA* mice may also be secondary from reduced atherogenesis from these mice. Indeed, eosinophils also play other atherogenic roles such as promoting SMC apoptosis, activating platelets, and enhancing atherosclerotic lesion thrombus formation. Nevertheless, the positive associations of blood eosinophil counts or plasma ECP levels with aortic and arterial calcification scores from this study remain consistent with our observations from experimental atherosclerotic mice. Increased atherosclerotic lesion eosinophil contents and plasma mEar1 levels not only correlate with atherosclerotic lesion development, but the present data support their causal role in atherogenesis.

## Supplementary Material

ehad262_Supplementary_Data

## Data Availability

The data underlying this article are available in the article and in its online supplementary material.
