# Supplementary material for: Cationic proteins from eosinophils bind bone morphogenetic protein receptors promoting vascular calcification and atherogenesis
Source: Eur Heart J. 2023 Jun 3;44(29):2763–83. doi: 10.1093/eurheartj/ehad262 (PMC10393071; doi:10.1093/eurheartj/ehad262)
Supplement: ehad262_Supplementary_Data [file ehad262_supplementary_data.pdf]

Supplementary Materials

Cationic proteins from eosinophils bind bone morphogenetic protein receptors promoting vascular calcification and atherogenesis

Zhaojie Meng,[1*] Shuya Zhang,[1,2*] Wei Li,[3*] Yunzhe Wang,[1,4] Minjie Wang,[1] Xin Liu,[1] Cong-Lin Liu,[1,4] Sha Liao,[1] Tianxiao, Liu,[1] Chongzhe Yang,[1,5] Jes S. Lindholt,[6,7] Lars M. Rasmussen,[7,8] Lasse M. Obel,[7,8] Jane Stubbe,[9] Axel C. Diederichsen,[7,10] Yong Sun,[11,12] Yabing Chen,[11,12] Paul B. Yu,[1] Peter Libby,[1] Junli Guo,[2] Guo-Ping Shi[1]

1. Department of Medicine, Brigham and Women's Hospital and Harvard Medical School, Boston, MA 02115, USA
2. Hainan Provincial Key Laboratory for Tropical Cardiovascular Diseases Research & Key Laboratory of Emergency and Trauma of Ministry of Education, Institute of Cardiovascular Research of the First Affiliated Hospital, Hainan Medical University, Haikou 571199, China
3. College of Chinese Medicinal Materials, Jilin Agricultural University, Changchun 130118, Jilin, China
4. Department of Cardiology, the First Affiliated Hospital of Zhengzhou University, Zhengzhou, China
5. Department of Geriatrics, National Key Clinical Specialty, Guangzhou First People's Hospital, Guangzhou Medical University, Guangzhou 510000, China
6. Department of Cardiothoracic and Vascular Surgery, Odense University Hospital, Odense, Denmark
7. Elite Research Centre of Individualized Treatment for Arterial Disease, University Hospital, Odense, Denmark
8. Department of Clinical Biochemistry, Odense University Hospital, Odense, Denmark
9. Cardiovascular and Renal Research unit, Institute for Molecular Medicine, University of Southern Denmark, Odense, Denmark
10. Department of Cardiology, Odense University Hospital, Odense, Denmark
11. Department of Pathology, The University of Alabama at Birmingham, Birmingham, AL 35294, USA
12. Birmingham VA Medical Center, Research Department, Birmingham, AL 35294, USA

*These authors contributed equally to this study.

Corresponding authors:
Guo-Ping Shi, ScD, Cardiovascular Medicine, Brigham and Women's Hospital, 77 Avenue Louis Pasteur, NRB-7, Boston, MA 02115, USA, Tel.: 617-525-4358, Email: gshi@bwh.harvard.edu,
Junli Guo, MD, PhD, Hainan Provincial Key Laboratory for Tropical Cardiovascular Diseases Research, The First Affiliated Hospital of Hainan Medical University, Haikou 571199, China, Tel.: 86-186 8983 5101, Email: guojl0511@126.com.

Table S1. Clinical characteristics and their association with blood eosinophil counts by non-parametric analysis.

**Variable**	**Yes**		**No**		
	**n**	**IQR**	**n**	**IQR**	***P***
Age >=70 years	1658	(0.11) 0.18 (0.27)	3928	(0.11) 0.17 (0.25)	0.283
Current or former smoker	3718	(0.12) 0.18 (0.28)	1801	(0.11) 0.17 (0.25)	<0.001
Hypertension	2560	(0.12) 0.18 (0.27)	3303	(0.11) 0.17 (0.26)	0.115
Diabetes Mellitus	636	(0.13) 0.20 (0.31)	5227	(0.11) 0.17 (0.27)	0.001
Comorbidity					
Previous stroke	413	(0.12) 0.18 (0.27)	5450	(0.11) 0.18 (0.27)	0.233
Previous AMI	348	(0.14) 0.21 (0.32)	5515	(0.11) 0.17 (0.27)	0.003
Previous PAD	139	(0.12) 0.20 (0.27)	5724	(0.11) 0.18 (0.27)	0.287
AAA	115	(0.11) 0.18 (0.28)	5748	(0.11) 0.18 (0.27)	0.357
Atrial fibrillation	782	(0.11) 0.18 (0.27)	5081	(0.11) 0.18 (0.27)	0.301
Previous coronary intervention	439	(0.12) 0.20 (0.30)	5424	(0.11) 0.17 (0.27)	0.076
Heart valve replacement	71	(0.13) 0.20 (0.28)	5792	(0.11) 0.18 (0.27)	0.951
Chronic pulmonary obstructive disease	405	(0.13) 0.21 (0.34)	5438	(0.11) 0.17 (0.27)	<0.001
Medication					
Use of low dose aspirin	1262	(0.12) 0.19 (0.29)	4601	(0.11) 0.17 (0.26)	0.008
Use of warfarin	400	(0.10) 0.19 (0.28)	5563	(0.12) 0.17 (0.26)	0.875
Use of Statin	1508	(0.12) 0.19 (0.28)	4355	(0.11) 0.18 (0.28)	0.107
Use of ACE inh or AT2-antagonists	1906	(0.12) 0.19 (0.28)	3957	(0.11) 0.17 (0.26)	0.047
Use of calcium blocker	1098	(0.12) 0.18 (0.28)	4765	(0.11) 0.18 (0.27)	0.428
Use of beta-agonist	174	(0.16) 0.25 (0.36)	5689	(0.11) 0.17 (0.27)	<0.001
Use of inhalation glucocorticoid	308	(0.14) 0.22 (0.33)	5555	(0.11) 0.17 (0.27)	0.008
Use of oral glucocorticoid	104	(0.07) 0.15 (0.27)	5759	(0.11) 0.18 (0.27)	0.017
Use of NSAID	182	(0.12) 0.18 (0.27)	5681	(0.11) 0.18 (0.27)	0.436
Blood pressure (systolic and diastolic)					
<140 mmHg and <90 mmHg	1912	(0.11) 0.18 (0.28)			0.049
140-159 mmHg or 90-99 mmHg	2349	(0.12) 0.18 (0.27)			
>160 mmHg or >100 mmHg	1603	(0.11) 0.16 (0.25)			
Body mass index (kg/m^2)					
<18.5	18	(0.10) 0.13 (0.23)			
18.5-25	1201	(0.10) 0.16 (0.26)			
25-30	2648	(0.11) 0.18 (0.27)			
>30	1675	(0.12) 0.19 (0.28)			<0.001

Table S2. Partial correlation between logarithmized calcification scores with eosinophil counts and other potential confounders.*

Calcification Scores**		Eosinophil count**	Smoking	Diabetes mellitus	COPD	Anti-platelet	ACE inhibitor	Gluco-corticoid, oral	Blood pressure group	BMI group
**Coronary artery**	**Partial Rho**	0.032	0.069	0.068	0.088	0.058	0.026	-0.020	-0.061	0.049
	***P* value**	0.017	<0.001	<0.001	<0.001	<0.001	0.054	0.133	<0.001	<0.001
**Aortic arch**	**Partial Rho**	0.035	0.070	0.068	0.089	0.057	0.027	-0.020	-0.061	0.049
	***P* value**	0.010	<0.001	<0.001	<0.001	<0.001	0.046	0.131	<0.001	<0.001
**Aortic valve**	**Partial Rho**	0.025	0.069	0.066	0.091	0.056	0.025	-0.019	-0.060	0.051
	***P* value**	0.068	<0.001	<0.001	<0.001	<0.001	0.066	0.157	<0.001	<0.001
**Infrarenal aorta**	**Partial Rho**	0.038	0.062	0.061	0.080	0.062	0.024	-0.029	-0.065	0.052
	***P* value**	0.011	<0.001	<0.001	<0.001	<0.001	0.105	0.050	<0.001	<0.001
**Iliac artery**	**Partial Rho**	0.039	0.063	0.059	0.082	0.062	0.022	-0.031	-0.066	0.050
	***P* value**	0.009	<0.001	<0.001	<0.001	<0.001	0.144	0.039	<0.001	<0.001

*: Multivariate analysis after adjusting for confounders associated with a *P* value less than 0.10 in Table S1.

**: Data were logarithmized by Log10.

Table S3. Primer sequences for RT-PCR.

Human genes	Primer sequences (5'—3')	Mouse gene	Primer sequences (5'—3')
ALP	AACATCAGGGACATTGACGTG	ALP	CCAACTCTTTTGTGCCAGAGA
	GTATCTCGGTTTGAAGCTCTTCC		GGCTACATTGGTGTTGAGCTTTT
BMPR-1A	TGAAATCAGACTCCGACCAGA	BMPR-1A	AACAGCGATGAATGTCTTCGAG
	TGGCAAAGCAATGTCCATTAGTT		GTCTGGAGGCTGGATTATGGG
BMPR-1B	CTTTTGCGAAGTGCAGGAAAAT	BMPR-1B	CCCTCGGCCCAAGATCCTA
	TGTTGACTGAGTCTTCTGGACAA		CAACAGGCATTCCAGAGTCATC
BMPR-2	CACTCAGTCCACCTCATTCATTT	BMPR-2	TTGGGATAGGTGAGAGTCGAAT
	TTGTTTACGGTCTCCTGTCAAC		TGTTTCACAAGATTGATGTCCCC
Collagen I	GAGGGCCAAGACGAAGACATC	Collagen I	TGTCGCTATCCAGCTGACCTTCCTGCG
	CAGATCACGTCATCGCACAAC		CCTCTGAGCTCGATCTCGTTGGATCC
GAPDH	GGCCTCCAAGGAGTAAGACC	GAPDH	AACTTTGGCATTGTGGAAGG
	AGGGGAGATTCAGTGTGGTG		GGATGCAGGGATGATGTTCT
Osteocalcin	CACTCCTCGCCCTATTGGC	Osteocalcin	CTGACCTCACAGATCCCAAGC
	CCCTCCTGCTTGGACACAAAG		TGGTCTGATAGCTCGTCACAAG
Osteopontin	CTCCATTGACTCGAACGACTC	Osteopontin	AGCAAGAAACTCTTCCAAGCAA
	CAGGTCTGCGAAACTTCTTAGAT		GTGAGATTCGTCAGATTCATCCG
Runx2	CCGCCTCAGTGATTTAGGGC	Runx2	CCACCACTCACTACCACACGTACCTG
	GGGTCTGTAATCTGACTCTGTCC		TAGCGTGCTGCCATTCGAGGTGGTG
TGFBR1	ACGGCGTTACAGTGTTTCTG	TGFBR1	TCTGCATTGCACTTATGCTGA
	GCACATACAAACGGCCTATCTC		AAAGGGCGATCTAGTGATGGA
TGFBR2	GTAGCTCTGATGAGTGCAATGAC	TGFBR2	CCGCTGCATATCGTCCTGTG
	CAGATATGGCAACTCCCAGTG		AGTGGATGGATGGTCCTATTACA

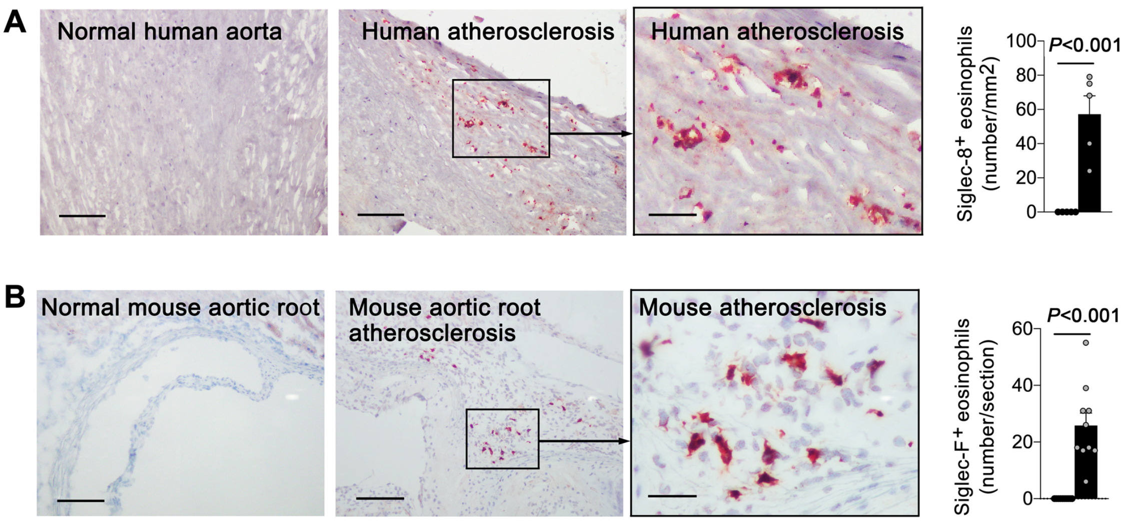

Figure S1. Eosinophils accumulate in human and mouse atherosclerotic lesions. **A.** Siglec-8 immunostaining of normal human aortas (n=5) and atherosclerotic lesions (n=5). Scale: 300 µm, inset: 100 µm. **B.** Siglec-F immunostaining of normal mouse aortic root (n=10) and atherosclerotic lesions in aortic root from *Apoe*$^{-/-}$ mice that were fed an atherogenic diet for 12 weeks (n=10). Scale: 300 µm, inset: 75 µm.

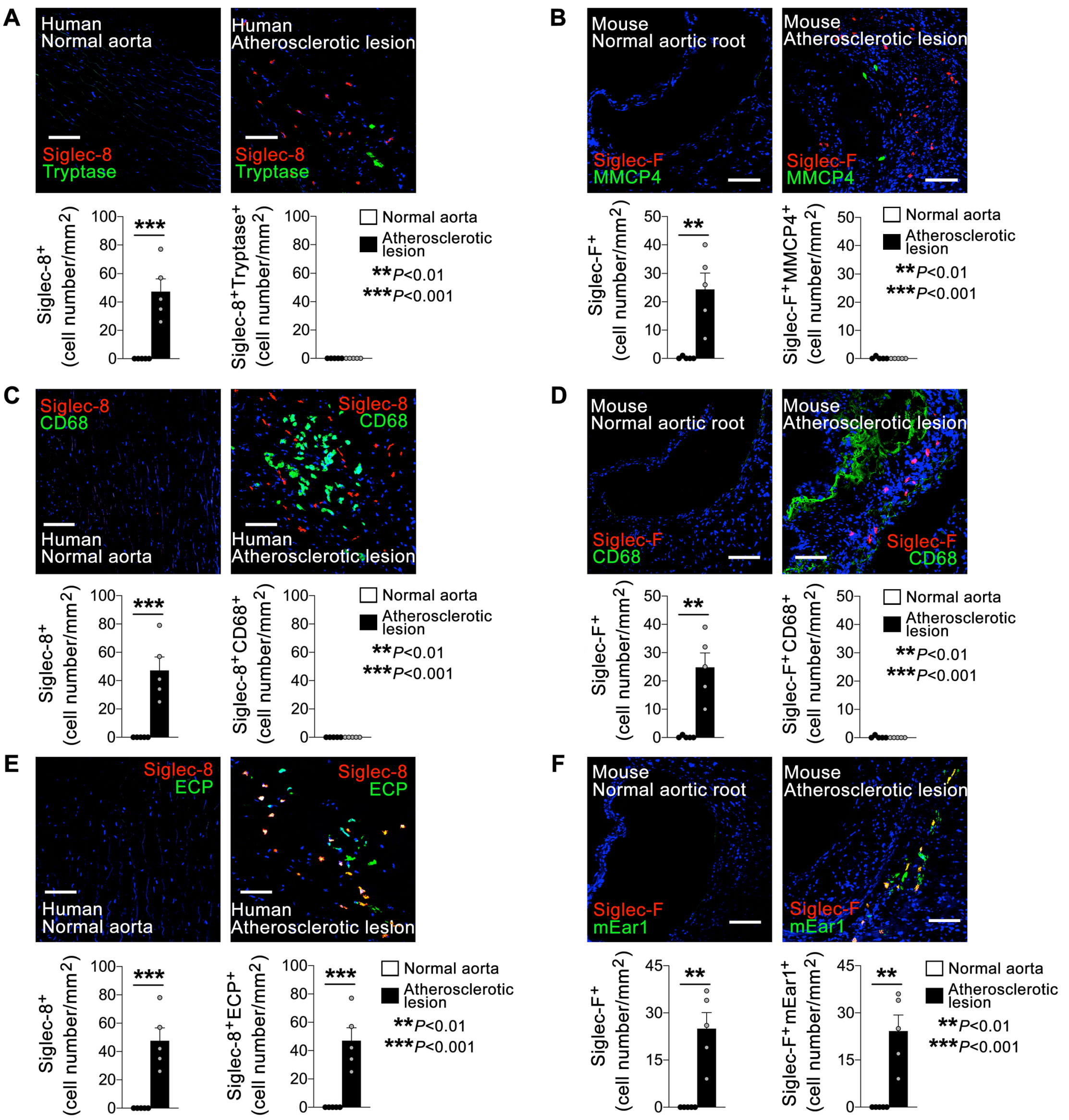

Figure S2. Immunofluorescence staining detects the expression of Siglec-8 and Siglec-F in human and mouse mast cells and macrophages and the expression of ECP or mEar1 in human and mouse eosinophils. **A.** Immunofluorescence double staining of mast cell tryptase and Siglec-8 in human normal aortas and atherosclerotic lesions. **B.** Immunofluorescence double staining of mouse mast cell chymase MMCP4 and Siglec-F in mouse normal aortic root and atherosclerotic lesions. **C.** Immunofluorescence double staining of macrophage CD68 and Siglec-8 in human normal aortas and atherosclerotic lesions. **D.** Immunofluorescence double staining of mouse macrophage CD68 and Siglec-F in mouse normal aortic root and atherosclerotic lesions. **E.** Immunofluorescence double staining of eosinophil Siglec-8 and ECP in human normal aortas and atherosclerotic lesions. **F.** Immunofluorescence double staining of eosinophil Siglec-F and mEar1 in mouse normal aortic root and atherosclerotic lesions. Representative images are shown to the left. Scale: 100 μm. Data are mean±SEM, n=5 per group.

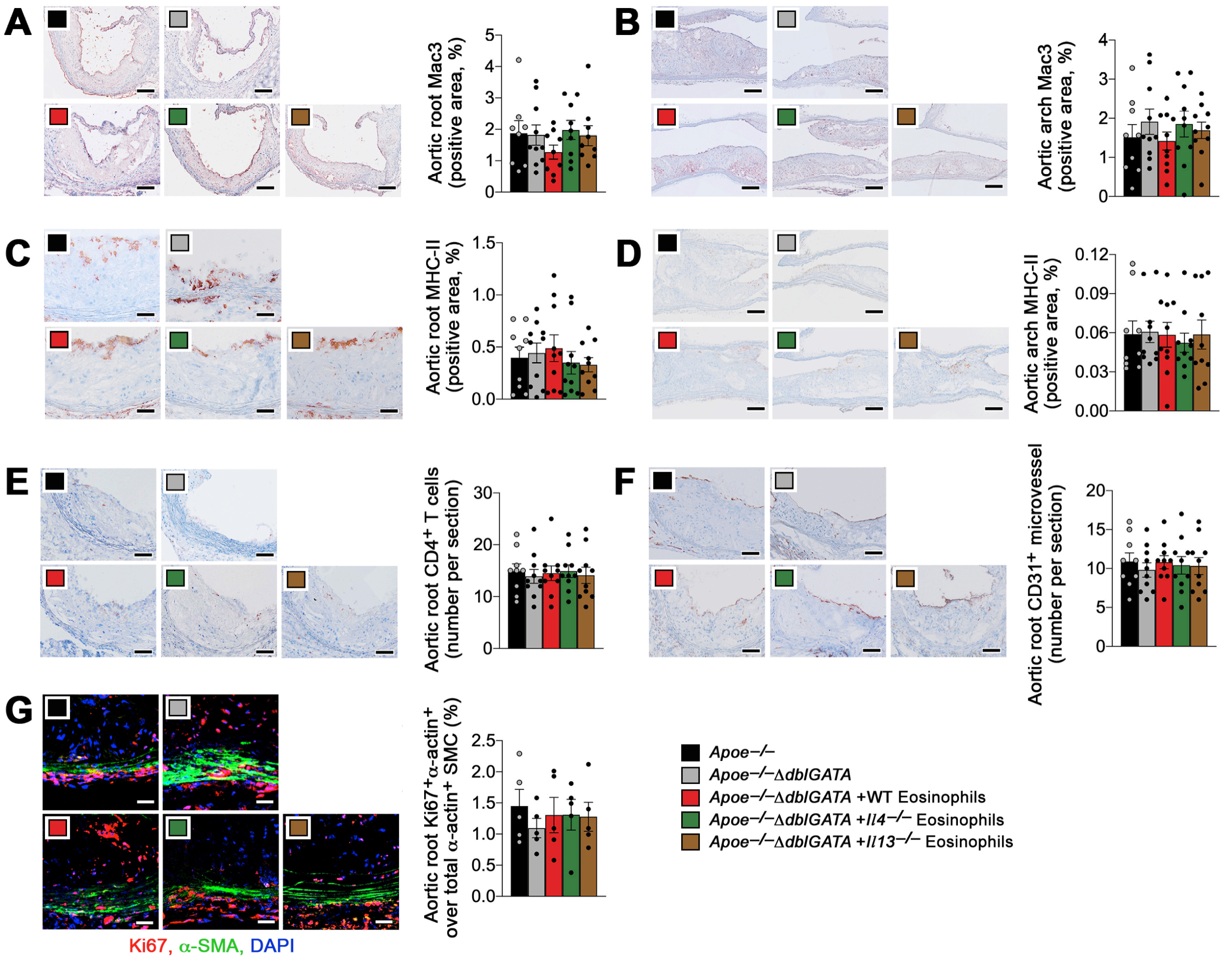

Figure S3. Eosinophils do not affect atherosclerotic lesion inflammation and SMC proliferation in *Apoe*$^{-/-}$ mice. *Apoe*$^{-/-}$ and *Apoe*$^{-/-}$*ΔdblGATA* mice were fed an atherosclerotic diet for 12 weeks. *Apoe*$^{-/-}$*ΔdblGATA* mice were also reconstituted with eosinophils from WT, *Il4*$^{-/-}$ or *Il13*$^{-/-}$ mice every two weeks. **A/B.** Aortic root and arch Mac-3^{+} macrophage-positive area. Scale: 200 μm. **C/D.** Root and arch MHC-II-positive area. Scale: 200 μm. **E.** Aortic root CD4^{+} T-cell number. Scale: 100 μm. **F.** Aortic root CD31^{+} microvessel number. Scale: 100 μm. **G.** Immunofluorescence double staining of α-actin and Ki67 to detect lesion SMC proliferation in aortic root. The results are expressed as mean±SEM of 9-10 (**A-F**) and 5 (**G**) mice per group.

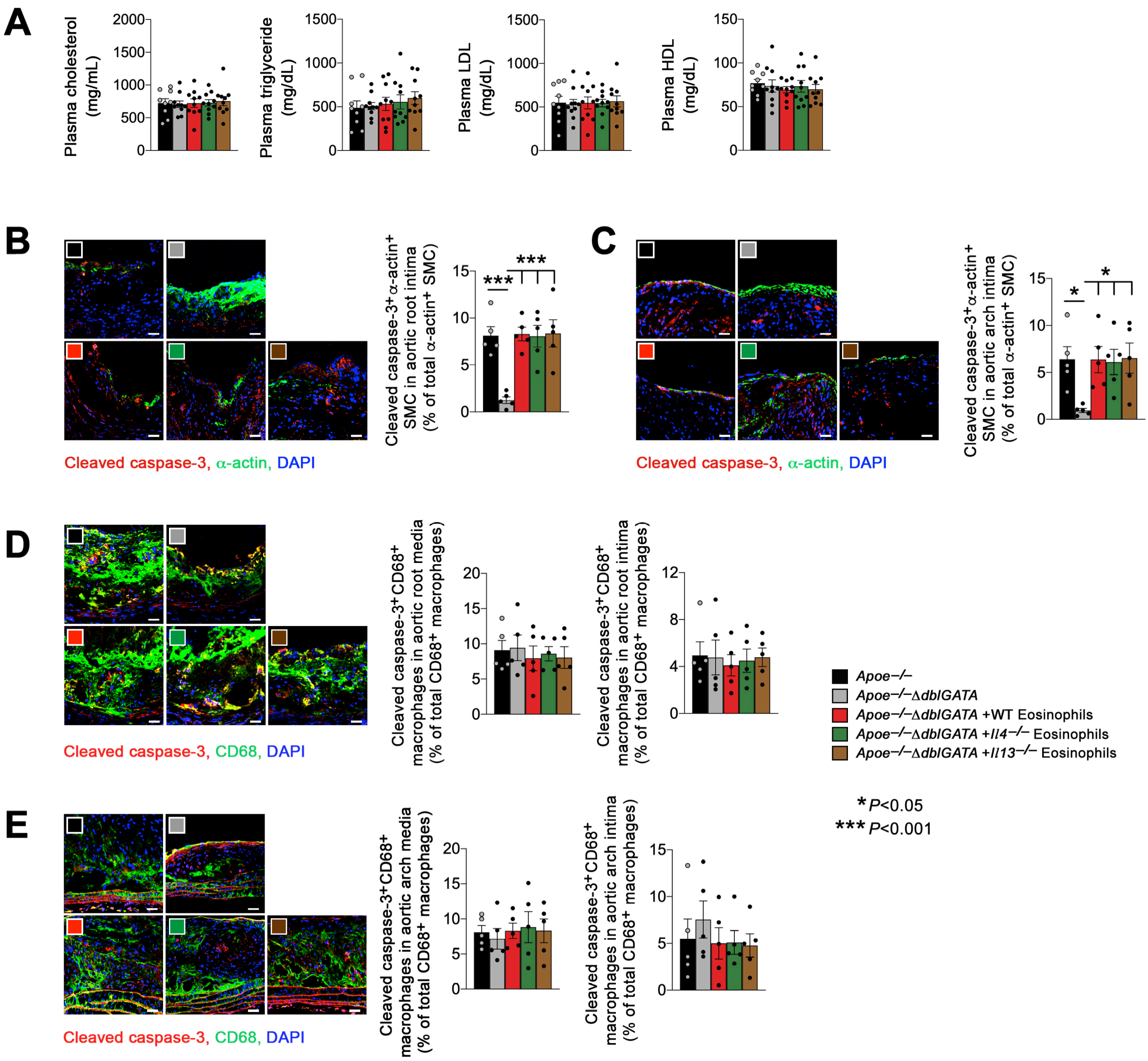

Figure S4. Eosinophil activities in plasma cholesterol levels in atherosclerotic mice and lesion SMC and macrophage apoptosis. **A.** Plasma total cholesterol, LDL, triglyceride, and HDL in *Apoe*$^{-/-}$, *Apoe*$^{-/-}$*ΔdblGATA*, and *Apoe*$^{-/-}$*ΔdblGATA* mice reconstituted with eosinophils from WT, *Il4*$^{-/-}$, or *Il13*$^{-/-}$ mice every two weeks and fed an atherosclerotic diet for 12 weeks. **B/C.** Immunofluorescence staining detected cleaved caspase-3 and α-actin double positive apoptotic SMC in atherosclerotic lesions in aortic root (**B**) and aortic arch (**C**) from the same groups of mice. **D/E.** Immunofluorescence staining detected cleaved caspase-3 and CD68 double positive apoptotic macrophages in atherosclerotic lesion media and intima in aortic root (**D**) and aortic arch (**E**) from these mice. Scale: 50 μm. The results are expressed as mean±SEM. n=9-10 per group in **A**. n=5 per group in **B-E**.

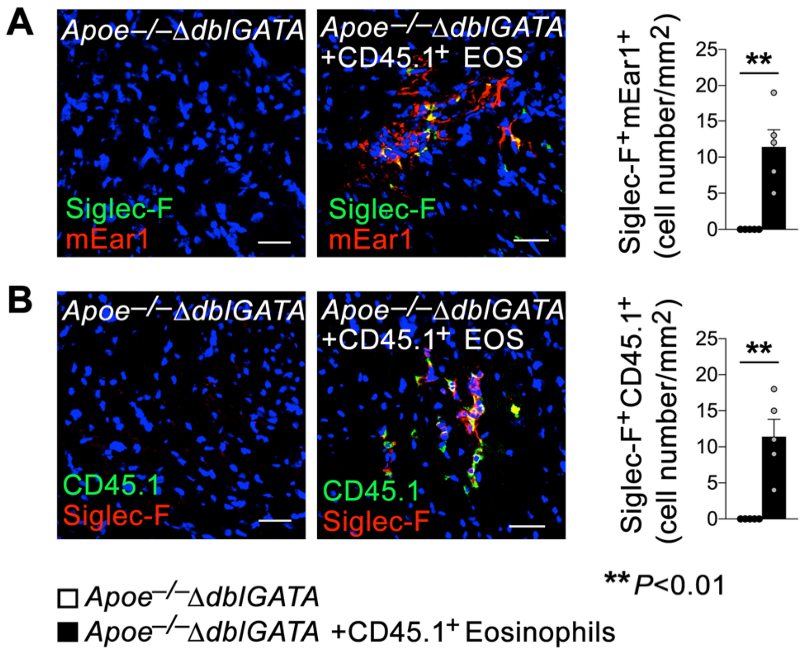

Figure S5. Donor eosinophil mEar1 expression in aortic root atherosclerotic lesions. **A-B.** Immunofluorescence double staining of Siglec-F with mEar1 (**A**) or with CD45.1 (**B**) of aortic root atherosclerotic lesions from *Apoe*$^{-/-}$*ΔdblGATA* mice and *Apoe*$^{-/-}$ *ΔdblGATA* mice reconstituted with eosinophils from CD45.1 transgenic mice. Representative images are shown to the left. Scale: 50 µm. Data are mean±SEM, n=5/each.

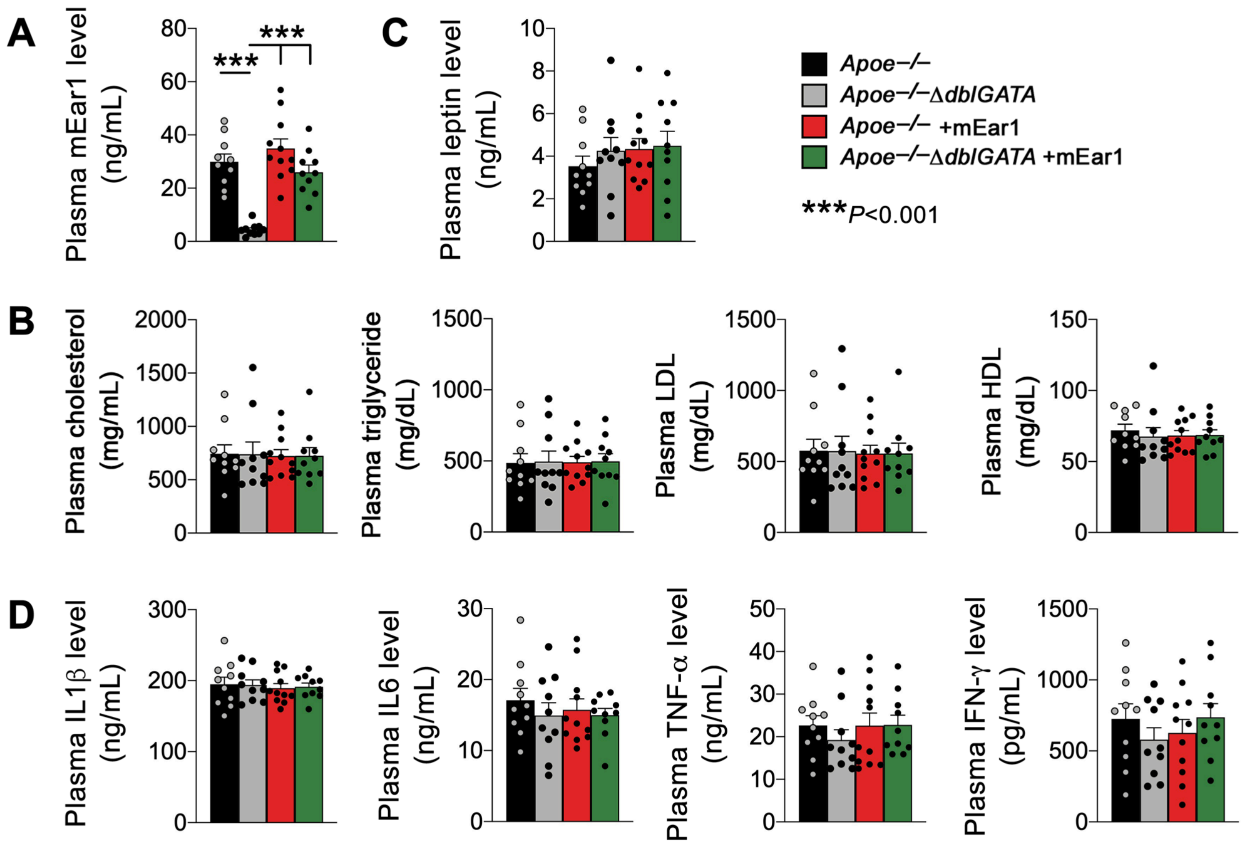

Figure S6. ELISA detection of plasma molecules in *Apoe*$^{-/-}$ and *Apoe*$^{-/-}$*ΔdblGATA* mice, and *Apoe*$^{-/-}$*ΔdblGATA* mice received mEar1 treatment. **A.** mEar1. **B.** Total cholesterol, LDL, triglyceride, and HDL. **C.** Leptin. **D.** Inflammatory cytokines, including IL1β, IL6, TNF-α, and IFN-γ. Mice were fed an atherosclerotic diet and administrated with recombinant mEar1 (5 μg/mouse/time) twice per week for 12 weeks. The results are expressed as mean±SEM of 9-10 mice per group.

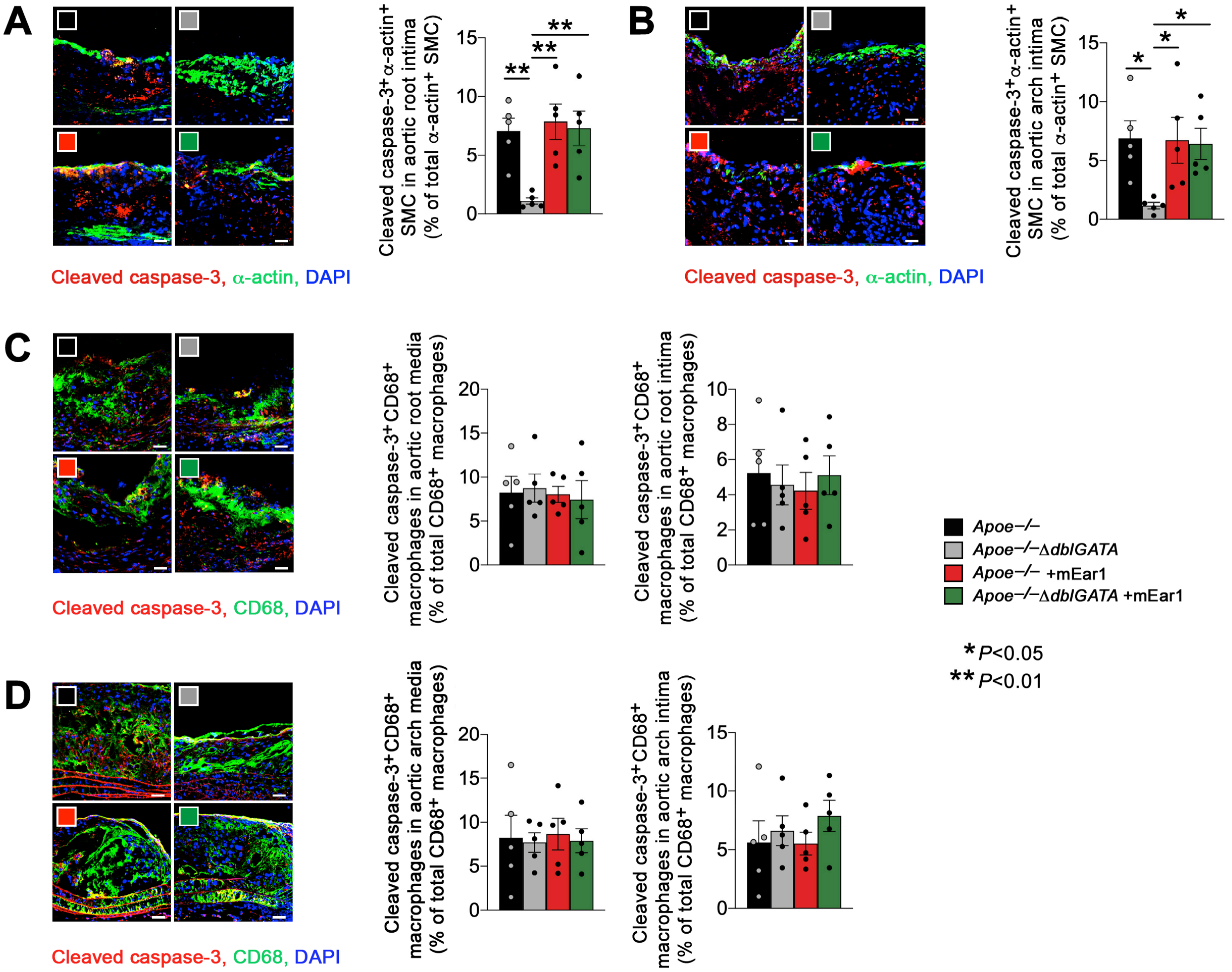

Figure S7. Eosinophil-derived mEar1 promote atherosclerotic lesion SMC apoptosis but do not affect macrophage apoptosis. **A/B.** Immunofluorescence staining detected cleaved caspase-3 and α-actin double positive apoptotic SMC in atherosclerotic lesions in aortic root (**A**) and arch (**B**) from *Apoe*$^{-/-}$ and *Apoe*$^{-/-}$*ΔdblGATA* mice, and those received mEar1 treatment. **C/D.** Immunofluorescence staining detected cleaved caspase-3 and CD68 double positive apoptotic macrophages in atherosclerotic lesion media and intima in aortic root (**C**) and arch (**D**) from the same groups of mice. Scale: 50 μm. The results are expressed as mean±SEM of 5 mice per group.

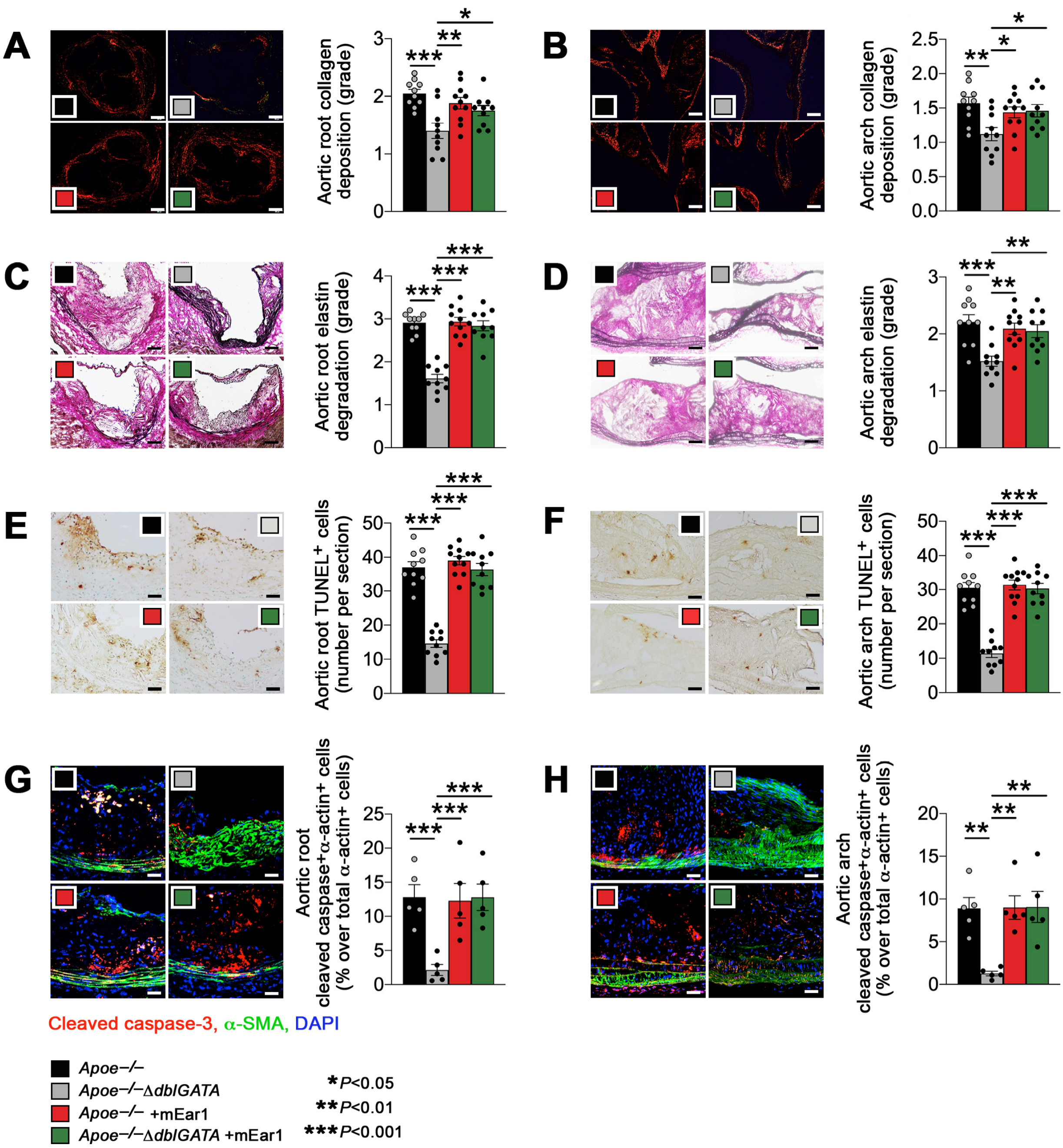

Figure S8. mEar1 enhances collagen deposition, elastin degradation, and cell apoptosis in *Apoe*$^{-/-}$ mice. *Apoe*$^{-/-}$ and *Apoe*$^{-/-}$*ΔdblGATA* mice were fed with an atherosclerotic diet for 12 weeks and administrated with or without recombinant mEar1 (5 μg/mouse/time) twice per week. **A/B.** Aortic root and arch Sirius red collagen staining in grade. Scale: 200 μm. **C/D.** Root and arch elastin fragmentation in grade. Scale: 200 μm. **E/F.** Root and arch TUNEL-positive cell contents. Scale: 100 μm. **G/H.** Immunofluorescence staining for root and arch cleaved caspase-3^{+}α-actin^{+} apoptotic SMC. Scale: 50 μm. The results are expressed as mean±SEM of 10-11 (**A-F**) and 5 (**G** and **H**) mice per group.

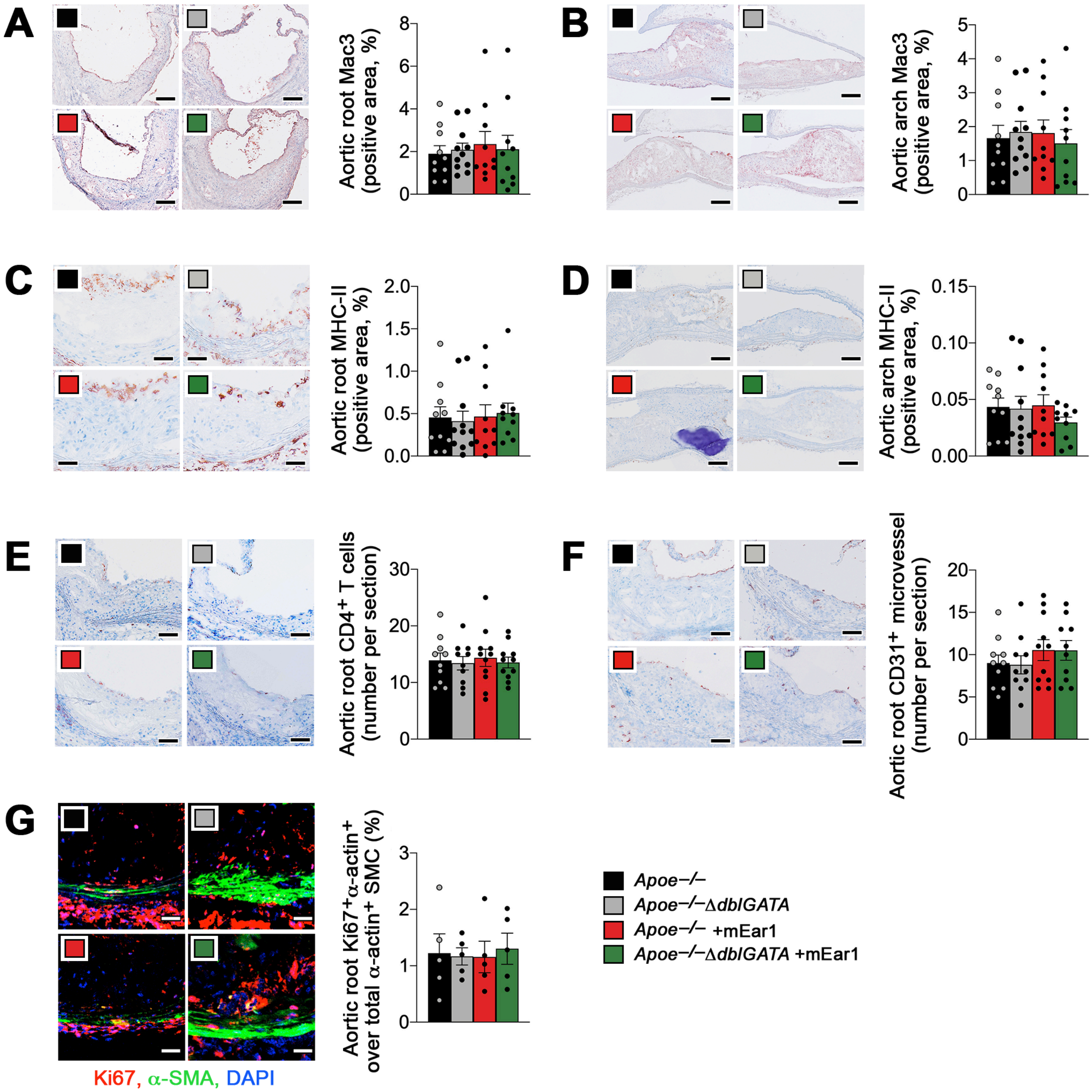

Figure S9. mEar1 does not affect atherosclerotic lesion inflammation and SMC proliferation in *Apoe*$^{-/-}$ or EOS-deficient *Apoe*$^{-/-}$ mice. *Apoe*$^{-/-}$ and *Apoe*$^{-/-}$*ΔdblGATA* mice were fed with an atherosclerotic diet for 12 weeks and administrated with or without recombinant mEar1 (5 μg/mouse/time) twice per week. **A/B.** Aortic root and arch Mac-3^{+} macrophage-positive area. Scale: 200 μm. **C/D.** Root and arch MHC-II-positive area. Scale: 200 μm. **E.** Aortic root CD4^{+} T-cell number. Scale: 100 μm. **F.** Aortic root CD31^{+} microvessel number. Scale: 100 μm. **G.** Immunofluorescence double staining of α-actin and Ki67 to detect lesion SMC proliferation in aortic root. The results are expressed as mean±SEM of 10-11 (**A-E**) and 5 (**G**) mice per group.

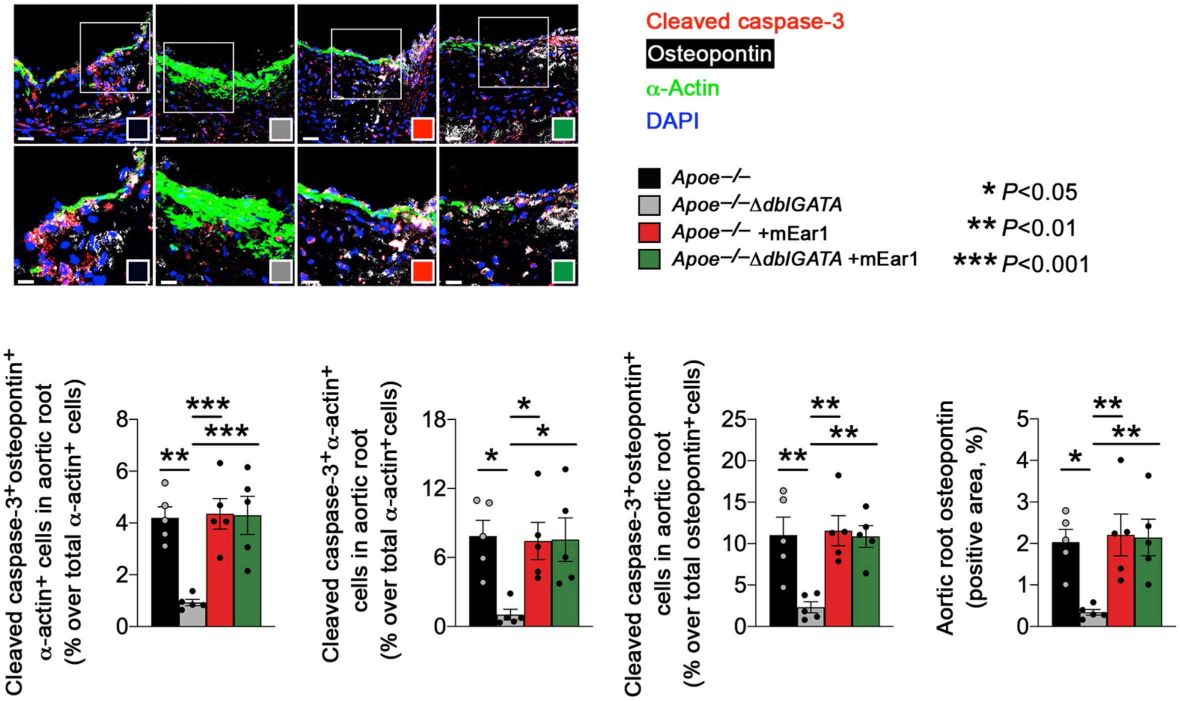

Figure S10. Immunofluorescence triple staining of aortic root atherosclerotic lesion apoptotic and calcified SMC in *Apoe*$^{-/-}$ and *Apoe*$^{-/-}$*ΔdblGATA* mice, and *Apoe*$^{-/-}$ *ΔdblGATA* mice received mEar1 treatment. Results include cleaved caspase-3^{+}osteopontin^{+}α-actin^{+} apoptotic and calcified SMC, cleaved caspase-3^{+}α-actin^{+} apoptotic SMC, cleaved caspase-3^{+}osteopontin^{+} apoptotic and calcified cell area, and osteopontin^{+} calcified cell area. Scale: 50 μm (top) and 25 μm (bottom). The results are expressed as mean±SEM of 5 mice per group.

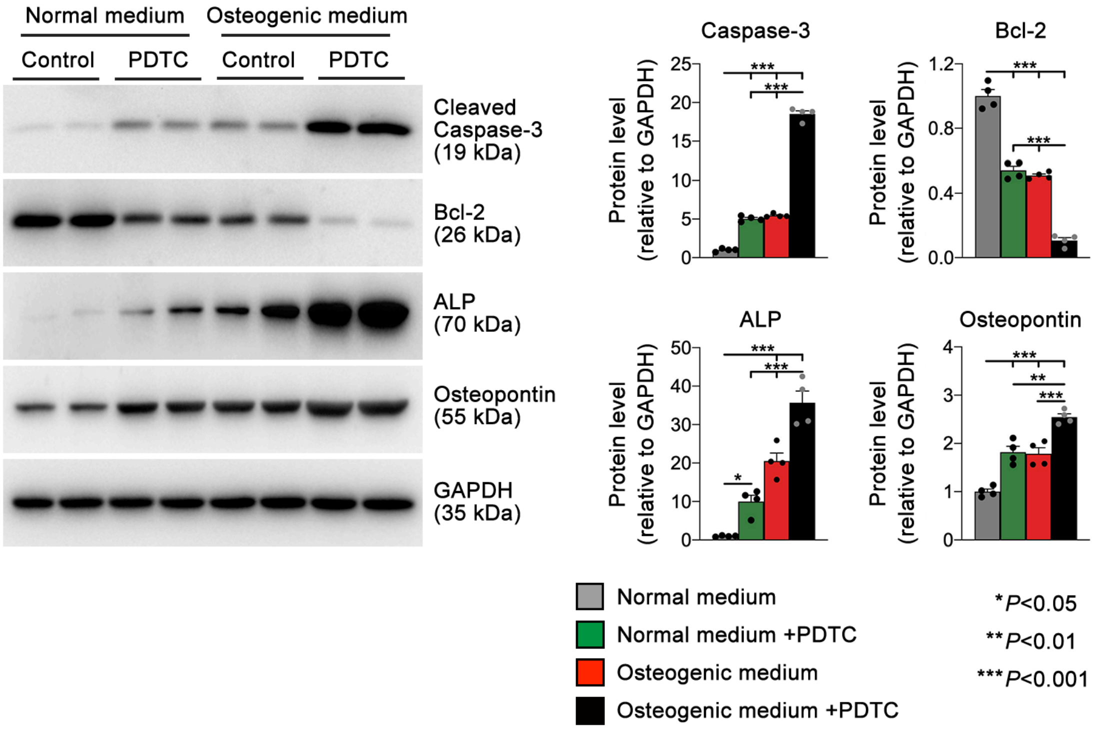

Figure S11. Interactions between calcification and apoptosis. WT mouse SMC were cultured in normal or osteogenic media and treated with or without 20 μM of PDTC to induce apoptosis. Use of immunoblots detected cleaved caspase-3, Bcl-2, alkaline phosphatase (ALP), and osteopontin. The results are expressed as mean±SEM of 5 mice per group.

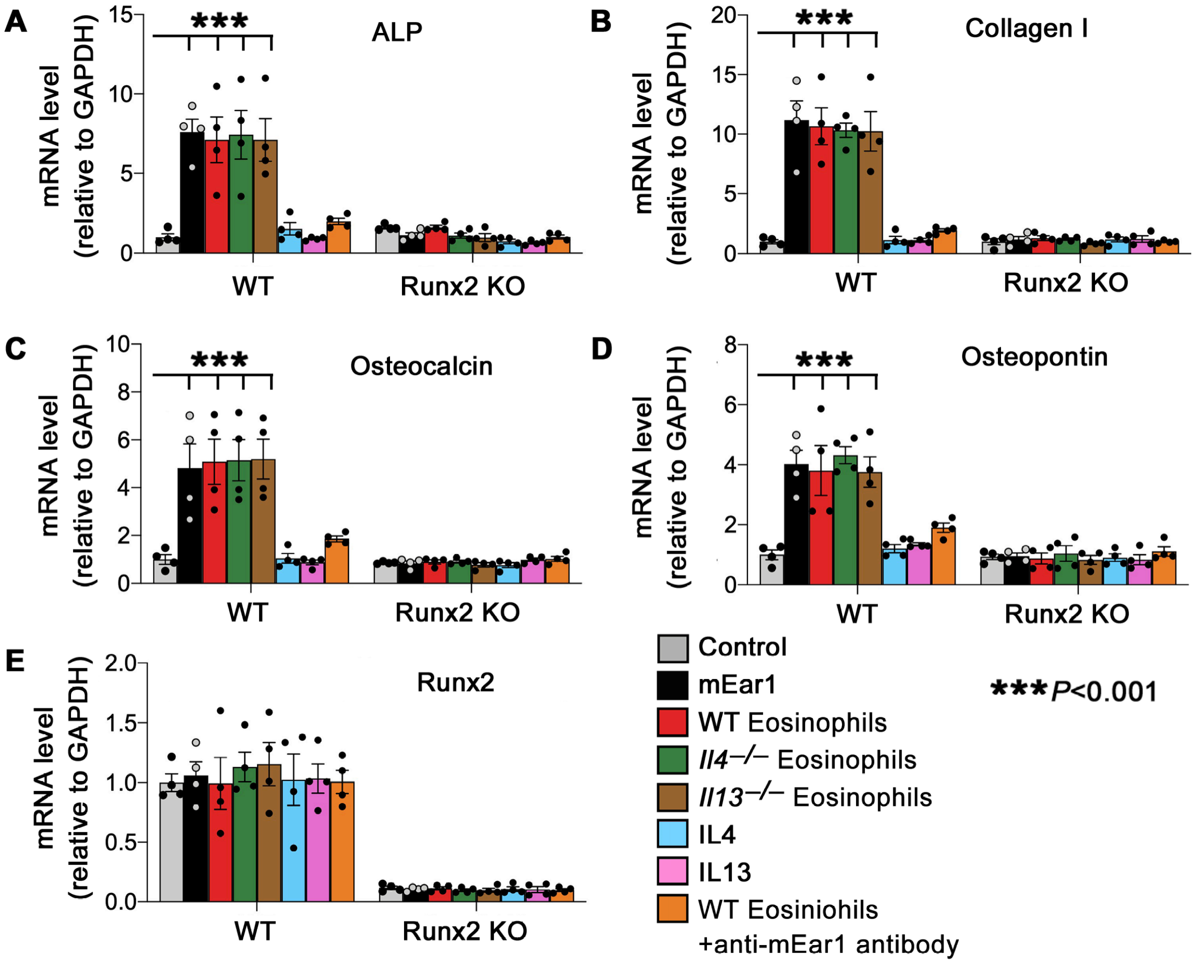

Figure S12. Eosinophil mEar1 but not IL4 or IL13 increases SMC osteogenic gene expression. Mouse aortic SMC from WT and Runx2 KO mice were exposed to osteogenic media with or without mEar1, IL4, IL13, eosinophil lysates from WT, *Il4*$^{-/-}$ or *Il13*$^{-/-}$ mice, or WT eosinophil lysate plus anti-mEar1 antibody. RT-PCR detected the expression of ALP (**A**), collagen I (**B**), osteocalcin (**C**), osteopontin (**D**), and Runx2 (**E**) genes. The results are expressed as mean±SEM of 4 independent experiments.

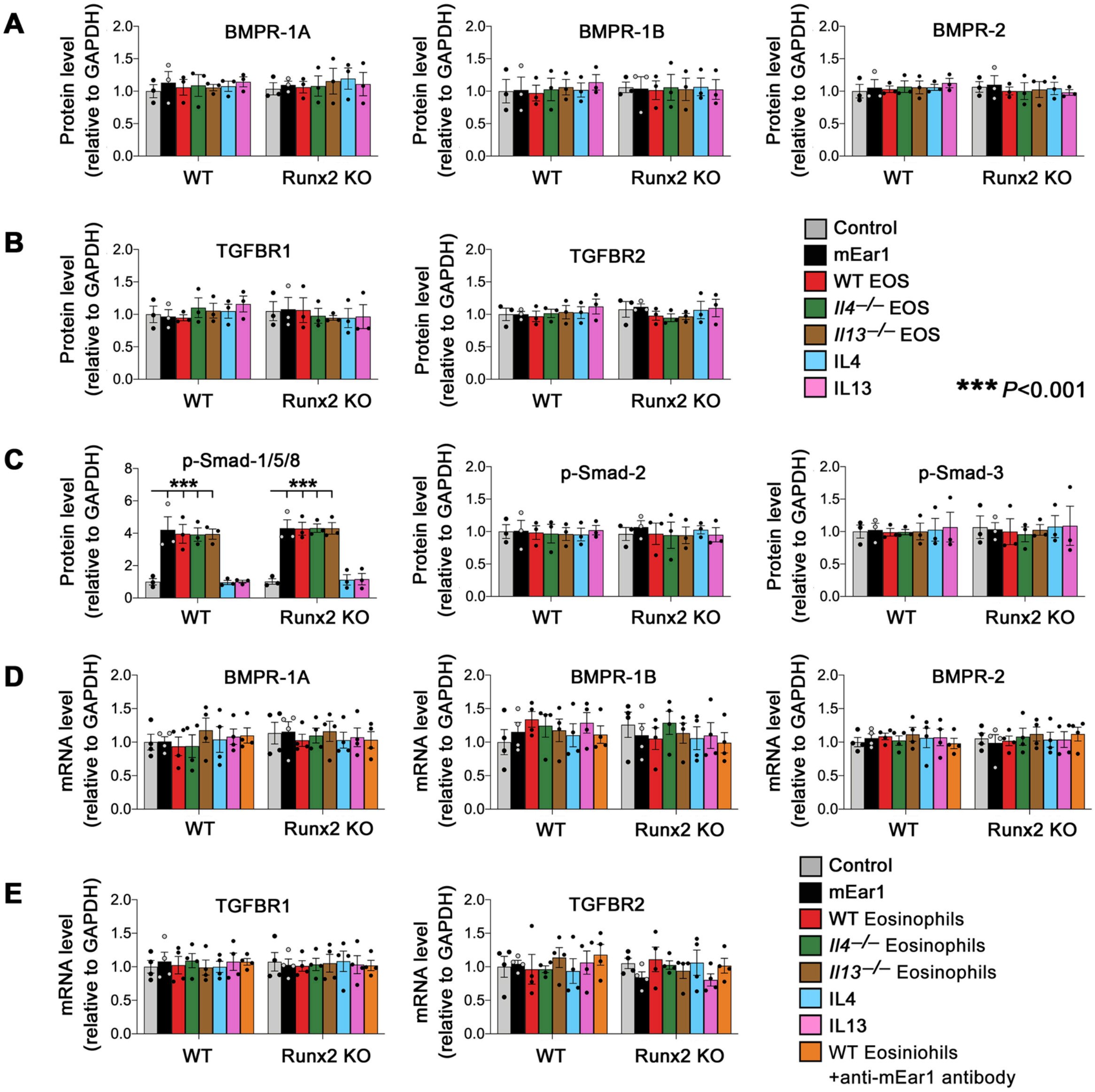

Figure S13. mEar1 increases the p-Smad-1/5/8 levels without affecting BMPR expression or TGFβR-Smad-2/3 signaling. Aortic SMC from WT and Runx2 KO mice were exposed to osteogenic media with or without mEar1, IL4, IL13, EOS lysates from WT, *Il4*$^{-/-}$, and *Il13*$^{-/-}$ mice, and WT eosinophil lysate plus anti-mEar1 antibody. Immunoblot detected the expression of BMPRs (**A**), TGFBRs (**B**), and phosphorylated Smads (**C**). RT-PCR detected the expression of BMPR (**D**) and TGFBR (**E**) genes. Data are mean±SEM from 3~4 independent experiments.

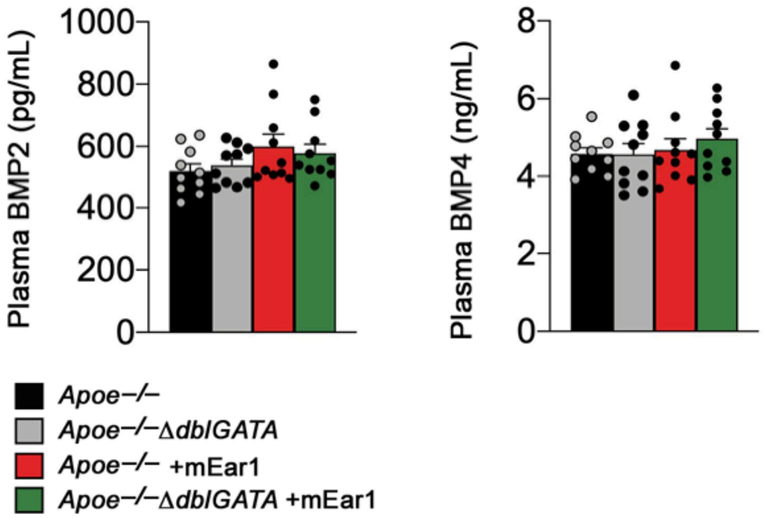

Figure S14. mEar1 does not affect plasma BMP2 or BMP4 in *Apoe*$^{-/-}$ or eosinophil-deficient *Apoe*$^{-/-}$ mice. *Apoe*$^{-/-}$ and *Apoe*$^{-/-}$*ΔdblGATA* mice were fed with an atherosclerotic diet for 12 weeks and administrated with or without recombinant mEar1 (5 µg/mouse/time) twice per week. ELISA detected plasma BMP2 and BMP4. Data are mean±SEM from 10 mice per group.

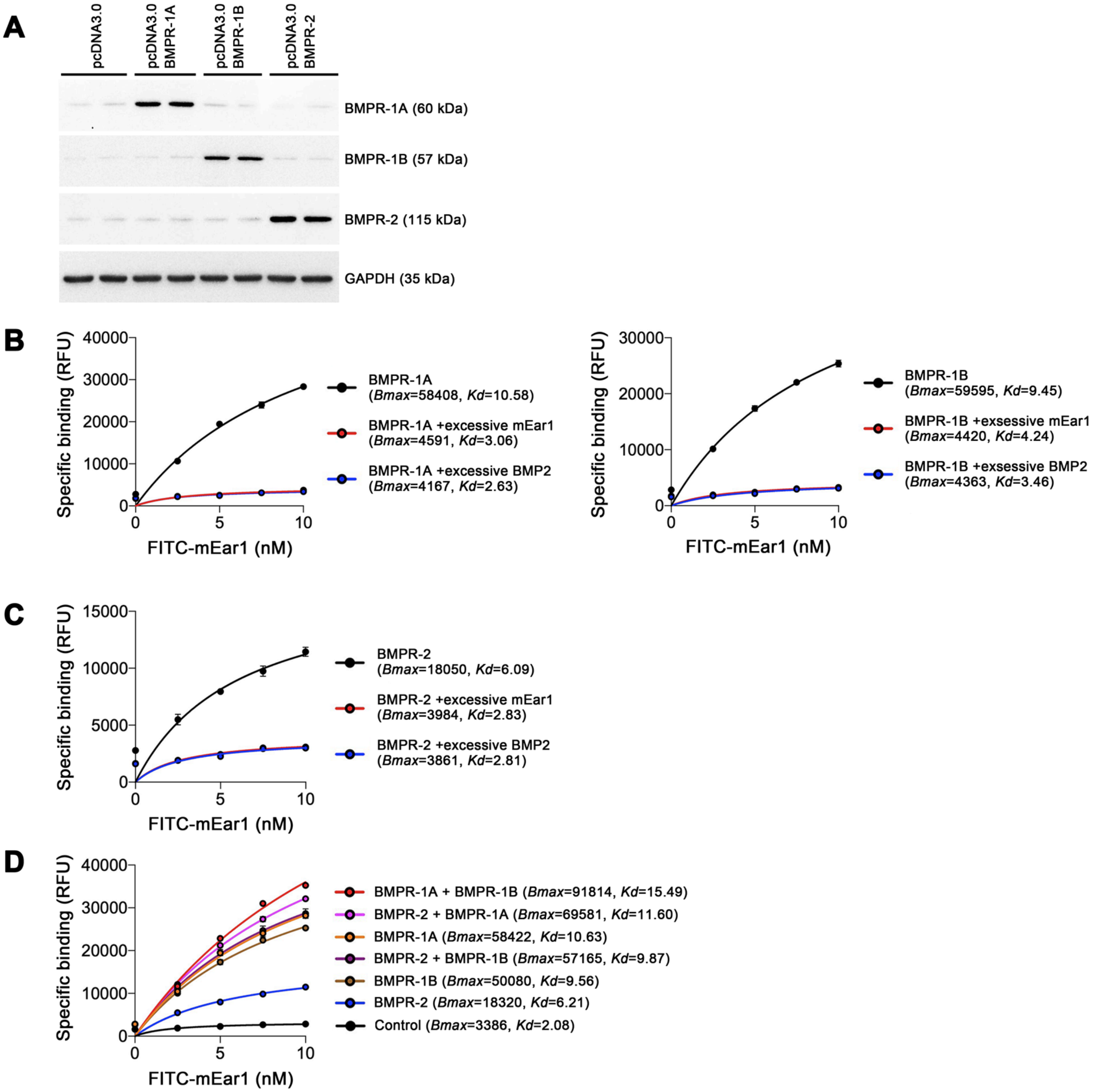

Figure S15. mEar1 binding assays on BMPR-transfected 293T cells. **A.** BMPR-1A, BMPR-1B, and BMPR-2 immunoblots validated the transfection efficacy of 293T cells. Empty vector was used as experimental control. **B/C.** Binding assays of FITC-mEar1 on 293T cells transfected with BMPR-1A or BMPR-1B (**B**), or on 293T cells transfected with BMPR-2 (**C**). **D.** Binding assays of FITC-mEar1 on 293T cells transfected with different BMPRs as indicated. FITC-mEar1 binding was also competed with excessive unlabeled mEar1 (5000 ng/mL) or BMP2 (1000 ng/mL) for 30 min.

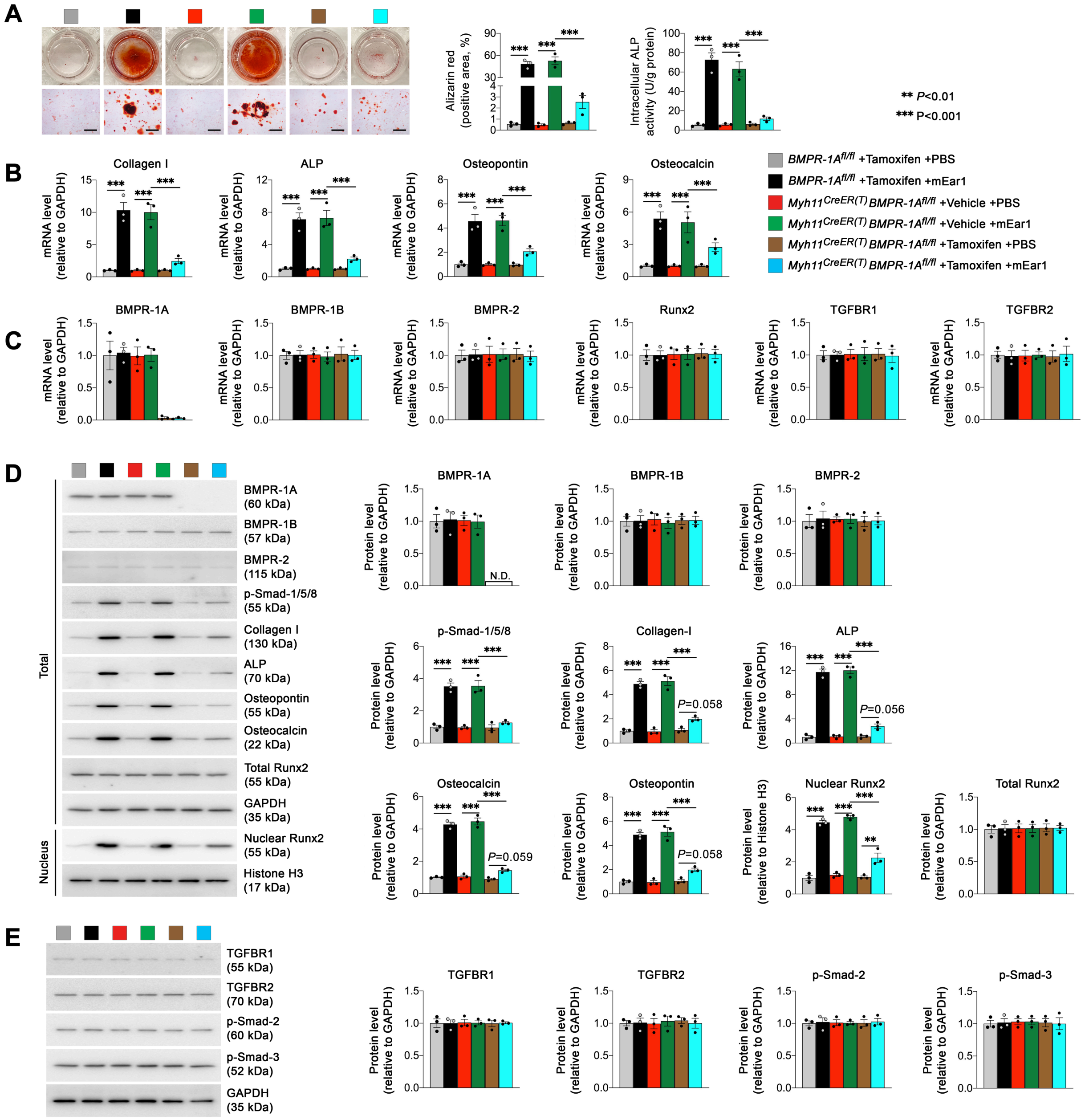

Figure S16. Tamoxifen-induced depletion of BMPR-1A in aortic SMCs blocks mEar1-induced calcification. Aortic SMCs were isolated from *BMPR-1A$^{fl/fl}$* and *Myh11$^{CreER(T)}$BMPR-1A$^{fl/fl}$* mice and treated with tamoxifen (0.5 µM) or vehicle (DMSO) for 24 hrs. Cells were then exposed to osteogenic media with or without mEar1 for 14 days. **A.** Representative images of Alizarin red-stained dishes (top) and photomicrographs (bottom, Scale: 200 um), and quantifications of Alizarin red staining for mineralized calcium and intracellular ALP activity. **B/C.** RT-PCR detected the expression osteogenetic genes (**B**), BMPRs, Runx2, and TGFBRs (**C**). **D/E.** Immunoblot analysis of BMPR signaling molecules and osteogenetic proteins (**D**) and TGF-β receptors and signaling molecules (**E**). Representative images are presented to the left (**A, D, E**). Data are mean±SEM from 3 independent experiments.

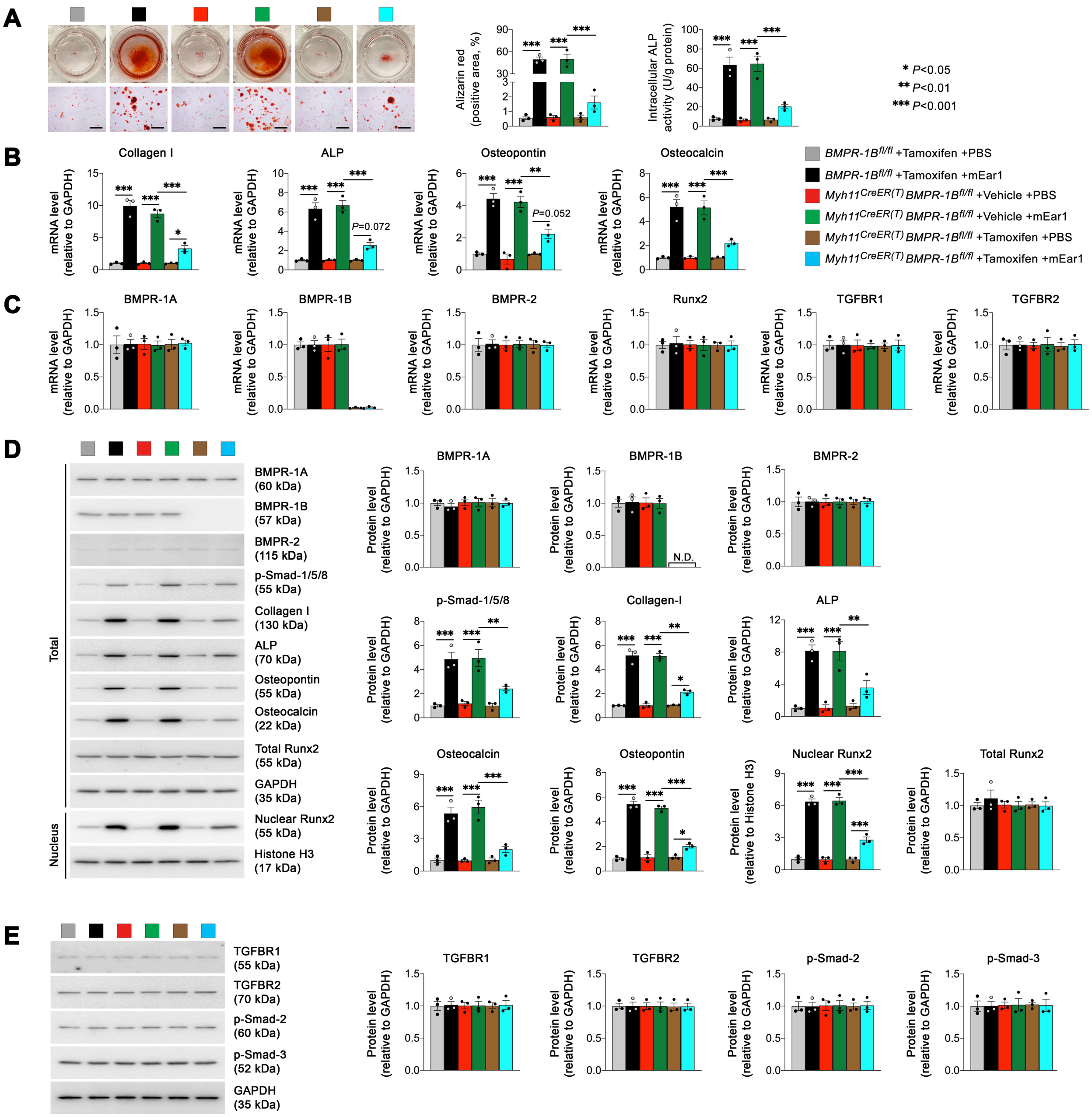

Figure S17. Tamoxifen-induced depletion of BMPR-1B in aortic SMCs blocks mEar1-induced calcification. Aortic SMCs were isolated from *BMPR-1B*$^{fl/fl}$ and *Myh11*$^{CreER(T)}$*BMPR-1B*$^{fl/fl}$ mice and treated with tamoxifen (0.5 µM) or vehicle (DMSO) for 24 hrs. Cells were then exposed to osteogenic media with or without mEar1 for 14 days. **A.** Representative images of Alizarin red-stained dishes (top) and photomicrographs (bottom, Scale: 200 um), and quantifications of Alizarin red staining for mineralized calcium and intracellular ALP activity. **B/C.** RT-PCR detected the expression osteogenetic genes (**B**), BMPRs, Runx2, and TGFBRs (**C**). **D/E.** Immunoblot analysis of BMPR signaling molecules and osteogenetic proteins (D) and TGF-β receptors and signaling molecules (**E**). Representative images are presented to the left (**A, D, E**). Data are mean±SEM from 3 independent experiments.

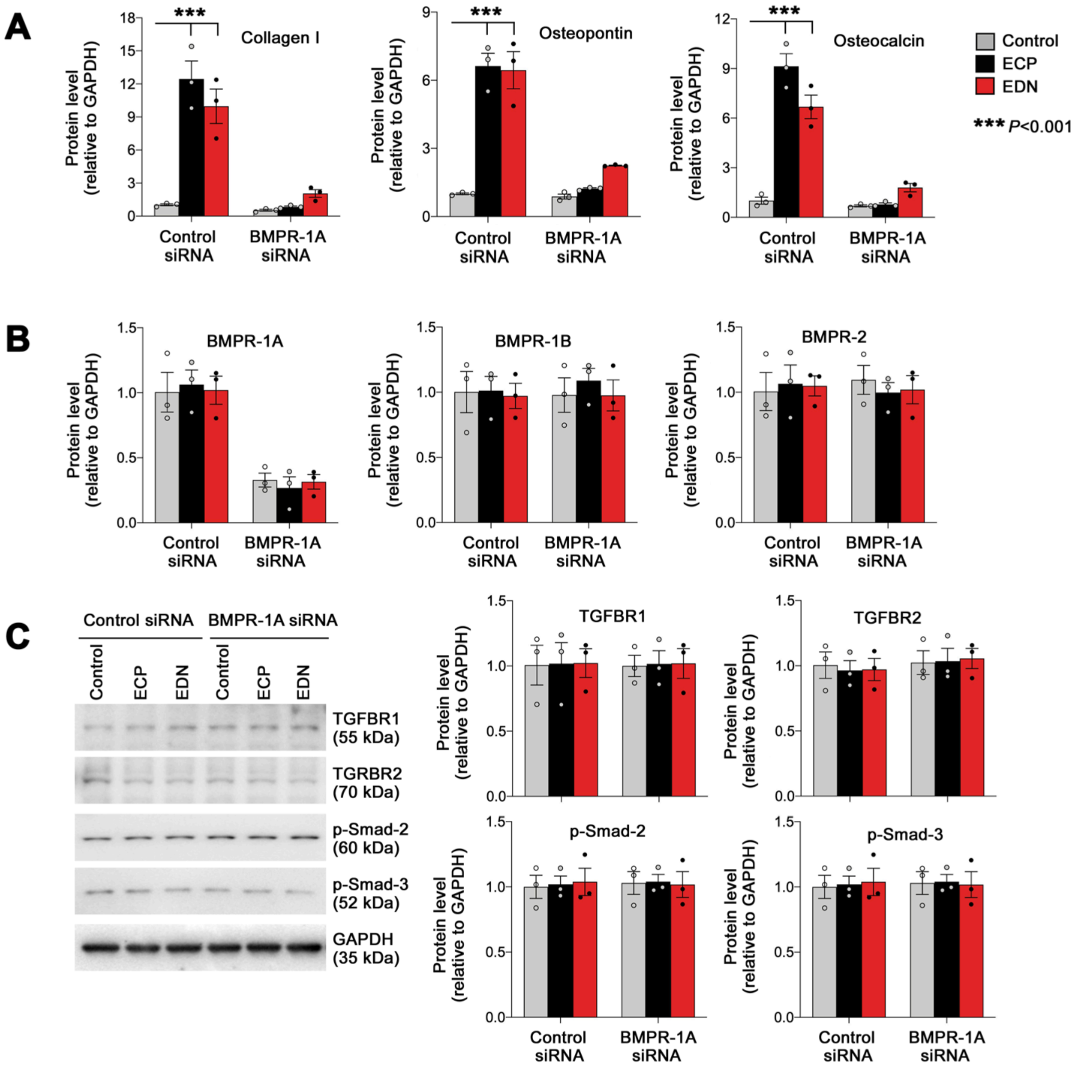

Figure S18. BMPR-1A mediates human ECP- and EDN-induced expression of osteogenic proteins in human SMC. Human vascular SMC were transfected with BMPR-1A or control siRNA, and then exposed to osteogenic media with or without ECP or EDN for 14 days. **A.** Immunoblot quantification of osteogenic proteins (collagen I, osteopontin, and osteocalin). **B.** Immunoblot quantification to ensure BMPR-1A knockdown without affecting BMPR-B or BMPR-2 in human vascular SMC. **C.** Immunoblot and quantification of TGFBR1/2 and phosphorylated Smad-2 and Smad-3. The results are expressed as mean±SEM of three independent experiments.

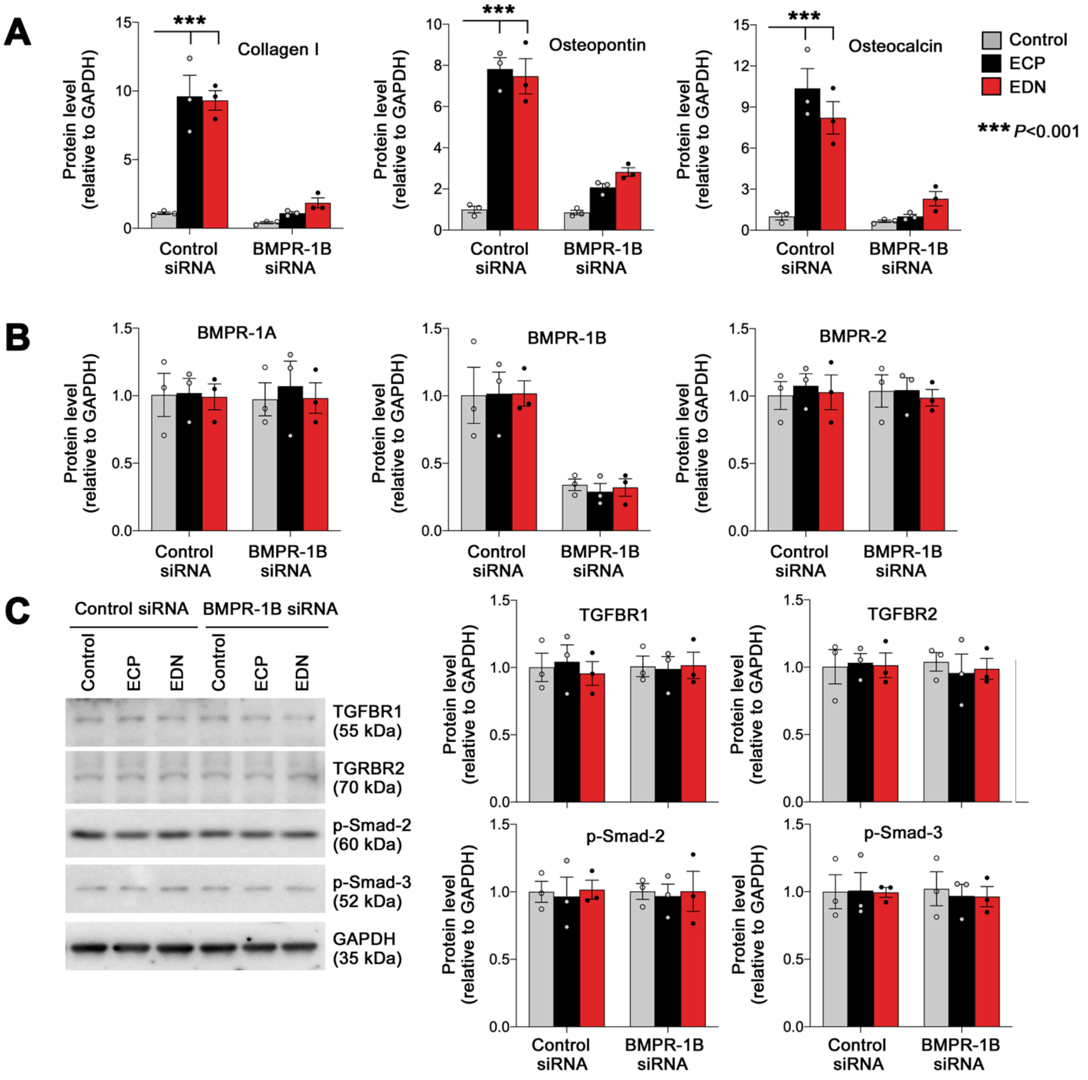

Figure S19. BMPR-1B mediates human ECP- and EDN-induced expression of osteogenic proteins in human SMC. Human vascular SMC were transfected with BMPR-1B or control siRNA, and then exposed to osteogenic media with or without ECP or EDN for 14 days. **A.** Immunoblot quantification of osteogenic proteins (collagen I, osteopontin, and osteocalin). **B.** Immunoblot quantification ensured the knockdown of BMPR-1B, but not BMPR-1A or BMPR-2 in human vascular SMC. **C.** Immunoblot and quantification of TGFBR1/2 and phosphorylated Smad-2 and Smad-3. The results are expressed as mean±SEM of three independent experiments.

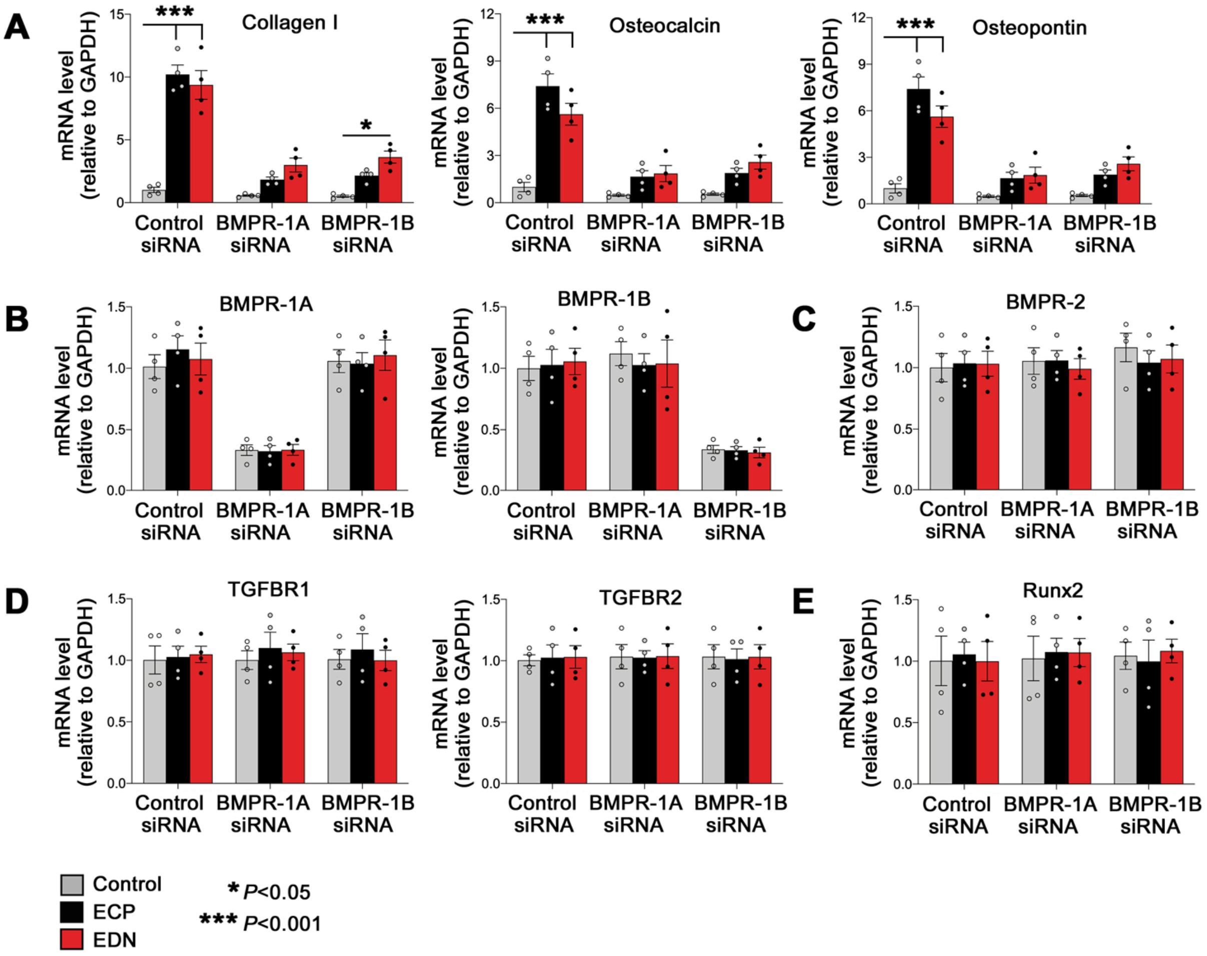

Figure S20. Human ECP- and EDN increase osteogenic gene expression *via* BMPR1A and BMPR-1B. Human SMC were transfected with BMPR-1A or BMPR-1B siRNA, and then exposed to osteogenic media with or without ECP or EDN for 14 days. RT-PCR detected the expression of BMPRs (**A** and **B**), TGFBRs (**C**), Runx2 (**D**) and osteogenic genes (**E**). The results are expressed as mean±SEM of 4 independent experiments.

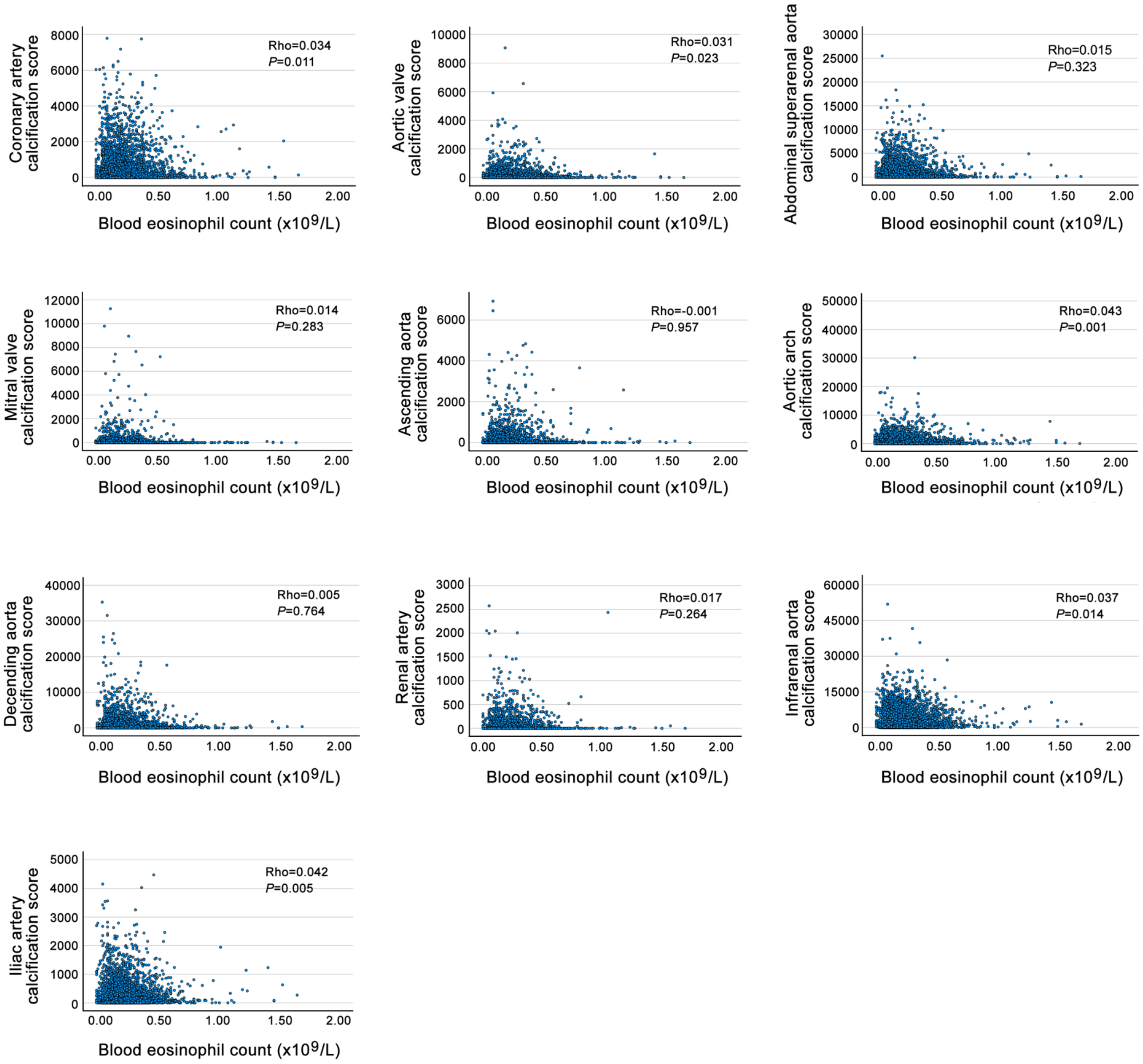

Figure S21. Scatter plots of non-parametric Spearman's correlations between blood eosinophil count (x10^9/L) and calcification scores of different part of aortas, arteries, and heart valves as indicated. The Rho and *P* values were adapted from the data in Table 1.

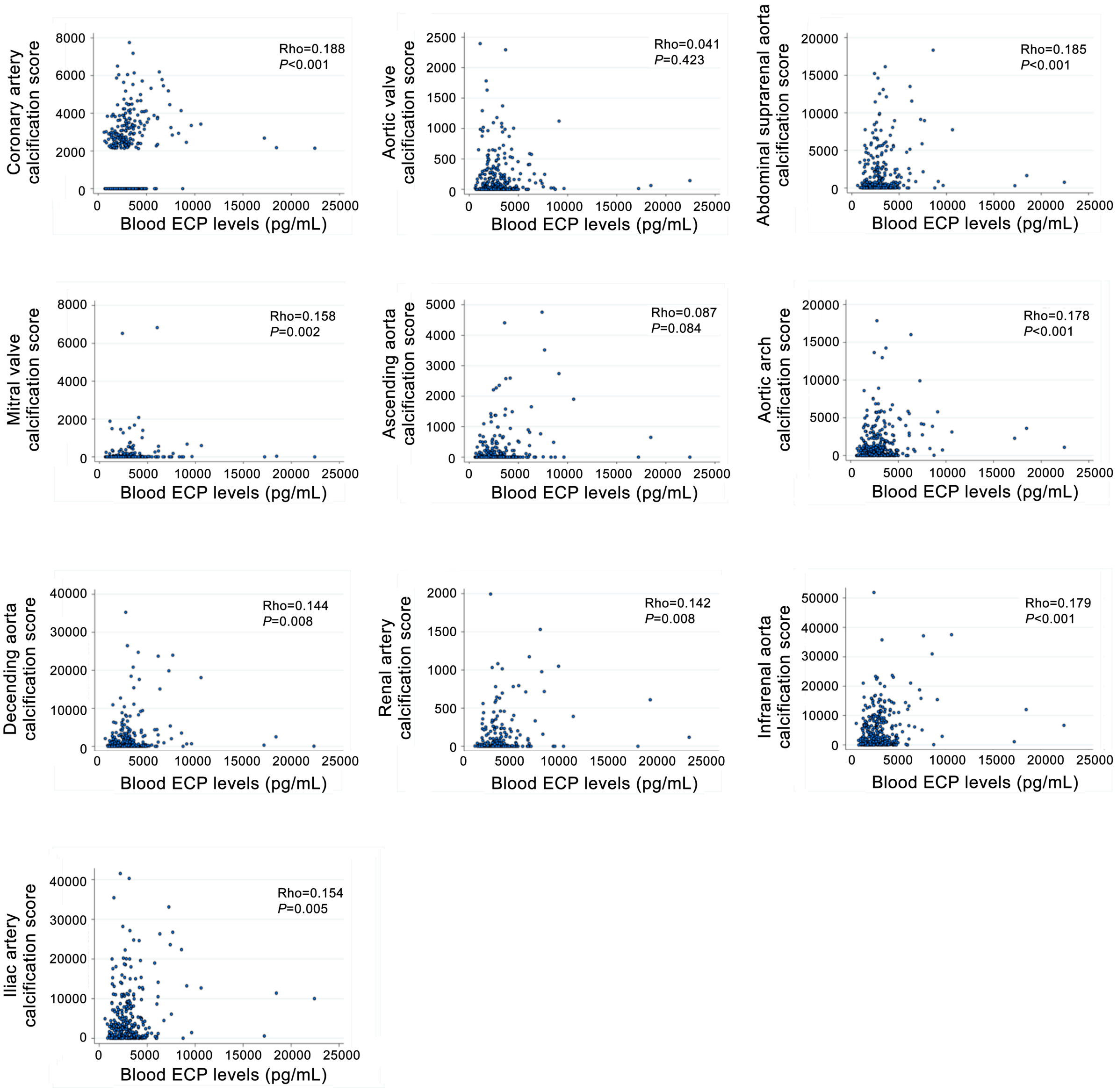

Figure S22. Scatter plots of non-parametric Spearman's correlations between blood ECP levels (pg/mL) and calcification scores of different part of aortas, arteries, and heart valves as indicated. The Rho and *P* values were adapted from the data in Table 1.
